# Interpreting *k*-mer–based signatures for antibiotic resistance prediction

**DOI:** 10.1093/gigascience/giaa110

**Published:** 2020-10-17

**Authors:** Magali Jaillard, Mattia Palmieri, Alex van Belkum, Pierre Mahé

**Affiliations:** bioMérieux, Chemin de l'Orme, 69280 Marcy l'Etoile, France; bioMérieux, Chemin de l'Orme, 69280 Marcy l'Etoile, France; bioMérieux, Chemin de l'Orme, 69280 Marcy l'Etoile, France; bioMérieux, Chemin de l'Orme, 69280 Marcy l'Etoile, France

**Keywords:** antibiotic resistance, supervised machine learning, *k*-mer, de Bruijn graph

## Abstract

**Background:**

Recent years have witnessed the development of several *k*-mer–based approaches aiming to predict phenotypic traits of bacteria on the basis of their whole-genome sequences. While often convincing in terms of predictive performance, the underlying models are in general not straightforward to interpret, the interplay between the actual genetic determinant and its translation as *k*-mers being generally hard to decipher.

**Results:**

We propose a simple and computationally efficient strategy allowing one to cope with the high correlation inherent to *k*-mer–based representations in supervised machine learning models, leading to concise and easily interpretable signatures. We demonstrate the benefit of this approach on the task of predicting the antibiotic resistance profile of a *Klebsiella pneumoniae* strain from its genome, where our method leads to signatures defined as weighted linear combinations of genetic elements that can easily be identified as genuine antibiotic resistance determinants, with state-of-the-art predictive performance.

**Conclusions:**

By enhancing the interpretability of genomic *k*-mer–based antibiotic resistance prediction models, our approach improves their clinical utility and hence will facilitate their adoption in routine diagnostics by clinicians and microbiologists. While antibiotic resistance was the motivating application, the method is generic and can be transposed to any other bacterial trait. An R package implementing our method is available at https://gitlab.com/biomerieux-data-science/clustlasso.

## Introduction

Antimicrobial resistance (AMR) is a global healthcare problem, and rapid diagnostics are needed to select the right treatment, to follow the route to cure, and to monitor and prevent community- and hospital-acquired outbreaks of infections. Next-generation sequencing is a disruptive technology that is, potentially, able to supplant or even replace the current plethora of diagnostic tests with a single, most probably well-affordable and faster solution. Inferring the antibiotic resistance profile from a bacterial genome is challenging. However, good results have been obtained for several species [1–7], including *Klebsiella pneumoniae* [8]. Su et al. [9] discussed the challenges of next-generation sequencing–based antibiotic susceptibility testing (AST) and provided a comprehensive review of the current state of the art in this field.

Early approaches relied on the detection of known resistance markers to claim resistance, a strategy sometimes referred to as “direct association analysis" [10]. While effective when the genetic bases of antibiotic resistance are well known, which is the case for instance for most antibiotic resistance mechanisms in the highly clonal species *Mycobacterium tuberculosis* [11, 12] and *Salmonella typhi* [13], this approach is hindered by several limitations. First and foremost, it intrinsically relies on prior knowledge of the precise nature of the resistance determinants, which may not be available for all species and drugs. Second, it is not able to account for the fact that these markers can have different levels of predictive power [14, 15], that they can act in a multi-factorial fashion through epistasis [16, 17], or that resistance can result from the accumulation of several different mutations [18, 19]. Last but not least, it is hazardous to predict susceptibility when no marker is detected because the resistance marker may be novel and databases incomplete.

Building AMR prediction models is now more and more addressed from the supervised machine learning (ML) standpoint: given a set of genomes with associated reference phenotypes (provided by phenotypic AST methods [20]), one seeks a prediction rule allowing inference of the resistance or susceptibility of a novel strain from genomic features.

While ML methods are also hindered by a completeness limitation because the set of genomes may not be representative of the genomic diversity of the whole species, they have the ability to identify novel markers or marker combinations in situations where no or limited prior knowledge is available and hence are becoming more popular in this context. Even for *M. tuberculosis*, where the antibiotic resistance knowledge is probably among the most thorough and complete, recent studies showed that performance of direct association strategies can still be significantly improved by ML models [10, 17].

A great variety of ML strategies have been explored, taking into account several parameters. First, regarding the nature of the genomic features considered: supervised ML models can indeed operate from known markers like the ones involved in direct association strategies, offering the possibility of discovering more complex and multivariate marker combinations better predicting resistance phenotypes [3, 10, 17], or directly using the raw sequences represented as *k*-mers [4, 8, 21–23]. The latter approach offers several advantages: it does not require prior knowledge about the underlying resistance mechanisms, allows the capture of various types of genomic determinants (including the acquisition of genes or point mutations), and does not require the genomes to be aligned to a common reference, which may be hard to define for some species, especially the less clonal ones [24, 25]. Second, regarding the type of ML algorithms, boosting algorithms [4, 8, 21], penalized regression models [10, 17, 23], decision trees [26], random forest [10, 27], neural networks [17], and set cover machines [22, 26] have already been successfully deployed in this context. While each algorithm has its own merits and shortcomings, several studies reported comparable global performance for various algorithms, with specific variations by drug and microbial species [10, 17, 28]. Finally, different kinds of antibiotic susceptibility information can be considered: either discrete when the objective is to distinguish susceptible from resistant (or non-susceptible) ones [10, 17, 21, 22], or continuous, where one seeks to predict the minimum inhibitory concentration (MIC) of the antimicrobial agent itself [3, 4, 8].

A critical challenge for the adoption of such predictive ML models by clinicians and microbiologists resides in their level of interpretability and, ultimately, clinical action-driving ability. While the notion of interpretability is ill defined, a natural requirement for the end-user would be to achieve the prediction from a limited number of genomic features that can be easily and unambiguously interpreted as actual genetic determinants [25, 26]. This challenge is particularly important in using *k*-mer–based representations, for several reasons.

First, *k*-mers covering conserved genomic regions are redundant, and while they can be easily detected and filtered [29], they define groups of equivalent *k*-mers, which are not always straightforward to interpret as genomic determinants [21–23, 26]. Second, *k*-mers may not be specific of a given genomic region and hence may be hard to annotate. This is especially the case for short *k*-mers, e.g., when *k* = 8 or *k* = 10 [4, 8]. Last but not least, the *k*-mer–based representation of genomes intrinsically leads to very high-dimensional feature spaces, with strongly correlated variables. Using *k* = 31 for instance, and depending on the bacterial species considered, it is common to end up working with 10^5^−10^6^ (non-redundant) *k*-mers, many of which are observed in almost the same sets of genomes, hence bringing almost the same information regarding the phenotype being studied.

We propose to rely on the adaptive cluster lasso (ACL) [30], an extension of Bühlmann et al. [31] tailored to the high-dimension setting by means of a prior screening of variables. We implemented in an R package a simple and efficient ACL-inspired strategy able to cope with the very high-dimensional and strong correlations of *k*-mer–based representation, leading to sparse and interpretable genomic signatures. This approach compared favorably to the standard lasso on a systematic validation study focusing on *K. pneumoniae*. It provided a comparable level of performance while offering better interpretability of the genomic determinants involved in the models. We could identify known and potentially novel resistance determinants from the corresponding *k*-mer signatures, which allowed the extraction of meaningful scientific insights.

## Methods

### Datasets

#### Training dataset

We gathered the assembled genomes, provided as contigs, of 1,665 strains to develop MIC prediction models for *K. pneumoniae* [8]. This set of genomes defines our training dataset. We focused on the 10 clinically most relevant antibiotics (listed in Table 1), which belong to 7 different antibiotic classes. The reference MICs were cast into resistant, susceptible, and intermediate according to the Clinical and Laboratory Standards Institute breakpoints. The intermediate and resistant strains were finally merged into a common category to define a binary classification problem aiming to distinguish susceptible (S) from non-susceptible (NS) strains. Table 1 provides the number of S/NS phenotypes available for each selected drug.

**Table 1:** Dataset constitution

Antibiotic	Training		Test	
	NS	S	NS	S
Amikacin	346	1,319	191	160
Aztreonam	1,426	216	250	10
Cefepime	961	608	235	53
Cefoxitin	976	667	319	138
Ceftazidime	1,529	136	457	125
Ciprofloxacin	1,461	201	471	137
Imipenem	504	1,160	259	301
Meropenem	524	1,134	297	86
Piperacillin/tazobactam	1,228	432	382	146
Tetracycline	928	737	273	155

This table provides the number of susceptible (S) and non-susceptible (NS) strains available in the training and test dataset for the various antibiotics considered. Note that a limited number of susceptible strains is available in the test dataset for aztreonam, and to a lesser extent cefepime and meropenem.

#### *k*-merization of the training dataset

**Figure 1:**
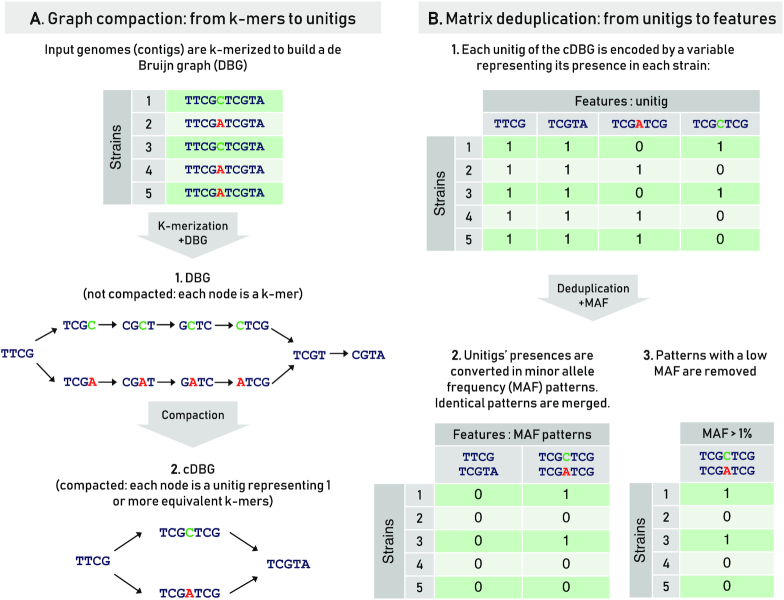
*k*-merization of the training genomes. Illustration of the DBGWAS process of *k*-merization and variant matrix construction. In this example, the 5 genomes differ only by one nucleotide (“C" in green for 2 genomes and “A" in red for 3 genomes). This SNP is then captured in the processing: each nucleotide variant corresponds to a distinct path in the cDBG (A2), however their corresponding complementary patterns are merged in the final matrix (B3). Refer to Jaillard et al. [25] for further details.

The *k*-merization was computed from the contigs of all training genomes, using the DBGWAS software [25], with a *k*-mer size of 31 and filtering patterns with a minor allele frequency (MAF) <1%. As discussed in previous studies [22, 25], *k*= 31 is a safe default choice, offering good predictive performance while preserving the specificity of the *k*-mers to particular genomic loci, which is key to annotating them properly. DBGWAS allows for the deduplication of the strictly equivalent *k*-mers by compacting overlapping non-branching paths of *k*-mers into unitigs, thanks to the use of a compacted de Bruijn graph (cDBG) (Fig. 1A). DBGWAS stores the profiles of presence/absence of each unitig in the training genomes in a matrix **V** such that **V**_*i, j*_ = 1 if the *j*th unitig is present in the *i*th input genome and **V**_*i, j*_ = 0 otherwise (Fig. 1B1). Each vector **V**_., *j*_is then transformed according to its allele frequency: if its allele frequency exceeds 0.5, meaning that it is observed in >$50\%$ of the panel genomes, it is inverted as **V**_*i, j*_ = |1 − **V**_*i, j*_| so that its MAF corresponds to its average value. This transformation renders identical 2 originally complementary vectors. Keeping only the unique patterns then leads to an optimal reduction of the number of features, without modifying the intrinsic statistical signal (Fig. 1B2). These unique, MAF-filtered patterns define the final variant matrix **X**, where **X**_*i, j*_ = 1 if the *j*th pattern is found in the *i*th genome, and 0 otherwise. This process is described in detail in Jaillard et al. [25]. The DBGWAS files describing the cDBG are kept for the further interpretation of the genomic signatures, allowing visualization of the unitigs of the selected patterns within their genomic environment.

In practice we carry out this *k*-merization process for each antibiotic separately, processing solely the strains that have been phenotypically tested. The output of this *k*-merization step is a sparse variant matrix **X** with, e.g., in the case of the cefoxitin antibiotic, *N* = 1,643 rows for the *N* cefoxitin-phenotyped strains of the training panel and *p* = 1,234,397 columns representing the *p* distinct patterns of presence/absence retained by DBGWAS. As discussed by Jaillard et al. [32], this offers a drastic reduction of the amount of information to store because the matrices based on *k*-mers and unitigs involve 85,623,165 and 3,396,675 columns, respectively. The matrix **X** is binary because DBGWAS only encodes the presence or absence in the genomes. It is sparse as only ~13% of the values are not null.

#### Test dataset

To validate the predictive performance of the models, we built an independent test dataset involving 634 strains, including 114 strains from our bioMérieux collection (NCBI Bioproject PRJNA449293 and PRJNA597427) and 520 strains from the PATRIC database (https://www.patricbrc.org/). Such strains were mostly from the USA, the UK, Serbia, Greece, and other European countries, and the MICs were obtained with either agar dilution, broth microdilution, or VITEK 2 (bioMérieux, Marcy l'Étoile, France) (see Supplementary Section S1). Table 1 provides the number of S/NS phenotypes available in the test dataset.

### Coping with highly correlated genomic features

Logistic regression is a widely used generalized linear model addressing binary classification problems. In our case, it consists of building a linear function defined for a strain represented by a vector **x** ∈ {0, 1}^*p*^ as:

(1) \begin{equation*}
\displaystyle f(\textbf{x}) = \beta_0 + \sum\nolimits_{j=1}^p \beta_j \textbf{x}_j, \end{equation*}

where *p* corresponds to the number of distinct patterns identified by DBGWAS and **x** encodes their presence/absence in the strain genome. To estimate the model coefficients and simultaneously select a limited number of patterns from a training panel of *n* strains, one can rely on the *L*_1_ or lasso penalty and consider the following optimization problem:

\begin{equation*} \hat{\beta } = \text{arg} \min _{\beta \in \mathbb {R}^{p+1}} \sum\nolimits _{i=1}^n \mathcal {L} \big ( y_i, f( {\bf X}_{i,.}) \big ) + \lambda \sum\nolimits _{j=1}^p | \beta _j|, \end{equation*}

where *y_i_* = 0 if the *i*th strain, stored in the *i*th row of the training matrix **X**, is susceptible and 1 otherwise. The function $\mathcal {L}$ is the logistic loss function, which quantifies the discrepancy between the true phenotypes *y_i_* of the strains and the predictions *f*(**X**_*i*, ._) obtained by the model. The λ parameter achieves a trade-off between this empirical error and the lasso regularization term and is usually optimized by cross-validation.

The feature selection ability of the lasso penalty is notoriously unstable in the presence of strong correlation between features. This is particularly the case using *k*-mer–based representations, making it difficult to derive meaningful interpretations from the features selected by the model, and their associated coefficients. We propose a simple and efficient 3-step strategy to identify sparse and interpretable genomic signatures.

#### Screening step

In this step, we “screen" features. For this purpose, we first fit a standard lasso-penalized regression model on the original feature matrix **X** for several values of the regularization parameter λ, and extract the set of features that are selected at some point on this regularization path. Formally, letting (λ_1_, ..., λ_*m*_) be the *m* values of the considered grid of λ, and **B** the *p* × *m* matrix containing the model coefficients obtained by Equation 1, we define a set **a** of “active features" as follows:

\begin{equation*} {\bf a} = \big \lbrace i \in [1,\dots ,p] \text{, such that} \,\, \max ( |{\bf B}_{i,.}| )\, \gt\, 0\big \rbrace , \end{equation*}

and let *p_a_* = |**a|** be their number. Because the lasso cannot select more features than there are observations, we typically end up with *p_a_* on the order of *N* (i.e., 10^3^ in our case). We then extract the features that are strongly correlated to the active ones from the entire feature matrix. For this purpose, we compute a *p_a_* × *p* matrix **G** containing the pairwise correlations between the *p_a_* active features identified beforehand and the *p* original ones. Formally, ${\bf G}_{i,j} = \tt {cor} ( {\bf X}_{.,{\bf a}_i} \, , \, {\bf X}_{.,j} )$, where “cor" is the standard Pearson correlation between vectors of MAF patterns across the genomes and is a classical criterion to quantify linkage disequilibrium (LD) between genomic features [33]. Because we rely on binary variables encoding the presence/absence of features in the genomes, **G**_*i, j*_ quantifies the extent to which features *i* and *j* co-occur in the genomes. Because *p_a_* is typically $\ll$*p* (on the orders of 10^3^ vs 10^6^ in our case), computing this matrix is much easier than computing the entire *p* × *p* correlation matrix. Finally, we extract the set **e** of features that are strongly correlated to ≥1 active feature as follows:

\begin{equation*} {\bf e} = \big \lbrace i \in [1,\dots ,p] \text{, such that} \,\, \max ( {\bf G}_{.,i})\, \gt\, s_1\big \rbrace , \end{equation*}

where the hyperparameter *s*_1_ controls the minimum level of correlation required and is referred to as the “screening threshold." This operation defines a set of *p_e_* = |**e**| features, called the set of “extended features." Obviously, we have *p_a_* ≤ *p_e_* ≤ *p*. In our context, we typically end up with a few thousand extended features, hence *p_a_* < *p_e_*$\ll$*p*.

#### Clustering step

While the screening step identifies a limited number of features deemed sufficiently correlated to the features identified by a standard lasso, the second step aims to explicitly define groups, or “clusters," of strongly correlated variables. We rely for this purpose on a bottom-up agglomerative clustering procedure, as suggested by Bühlmann et al. [31]. More precisely, we first define a *p_e_* × *p_e_* distance matrix **D** between extended features, defined as ${\bf D}_{i,j} = |1 - \tt {cor} ( {\bf X}_{.,{\bf e}_i} \, , \, {\bf X}_{.,{\bf e}_j})|$. This matrix is then used to carry out a hierarchical clustering, implemented in R by the hclust function, using a minimum linkage criterion. The resulting dendrogram is finally cut at a height of 1 − *s*_2_, the second hyperparameter *s*_2_, called the “clustering threshold," controlling the level of within-cluster correlation.

#### Learning step

**Figure 2:**
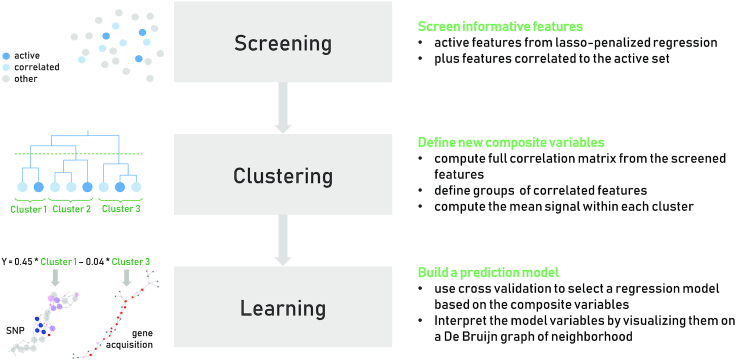
Three-step process. Illustration of the proposed 3-step procedure.

Finally, we summarize each identified cluster as a new composite variable, defined as the average of the original variables defining the cluster, and carry out a standard lasso at the cluster level. Because in our case the original variables encode the presence/absence of a given DBGWAS pattern in the genomes, these composite variables correspond to the proportion of patterns involved in a cluster that are present/absent in the genomes. Fig. 2 summarizes this 3-step method.

### Model selection

Our approach involves 3 hyperparameters that must be optimized for each antibiotic: the screening and clustering thresholds *s*_1_ and *s*_2_ used to build the clusters of correlated variables, and the regularization parameter λ involved in the final cluster-level lasso model. We relied on the glmnet software [34] to fit the lasso models involved in both the screening and learning steps. We used the default heuristic proposed by the software to define the grids of candidate values for the regularization parameters.

The screening and clustering thresholds were both systematically set to 0.95 on the basis of preliminary experiments (see Supplementary Section S2), and we relied on a 10-fold cross-validation procedure to optimize the regularization parameter involved in the final cluster-level lasso model. For this purpose, we first split the training dataset into 10 folds, stratified by sequence type and phenotype. This cross-validation strategy allows us to assess the impact of regularization on the predictive performance and feature selection ability of the model, in the absence of genetic drift between the training and test folds. We emphasize, however, that it may lead to an optimistic estimation of the predictive performance, which must not be considered as an estimate of the generalization of the model. The actual generalization of the model is subsequently estimated from the independent test set, using a model trained from the entire training set and the value of the regularization parameter optimized according to the following procedure.

For each of the 10 folds, nine-tenths of the training dataset were used to screen variables and identify clusters. The final cluster-level lasso model was then fit and applied to the held out strains (the test fold), for each candidate value of the regularization parameter. Our model selection strategy aimed to simultaneously maximize its sensitivity and specificity, respectively defined as the fractions of correctly classified non-susceptible and susceptible strains. For this purpose, a receiver operating characteristic (ROC) curve was built for each candidate regularization parameter after completion of the cross-validation procedure, and the point closest to the optimal one (defined by a true-positive rate of 1 and a false-positive rate of 0) was used to define the optimal sensitivity/specificity trade-off. Following Hicks et al. [28], we refer to the average of the (optimal) sensitivity and specificity as balanced accuracy (bACC). Finally, we selected the sparsest model that allowed maximization of the bACC up to 1 point, in order to reduce the risk of overfitting. In practice, this cross-validation procedure was repeated 3 times and the selection was based on average bACC values obtained across the 3 repetitions. Supplementary Fig. S5 illustrates this model selection strategy.

### Interpretation of the predictive signature

We use the DBGWAS software to interpret the genomic signatures, based on the cDBG built during the *k*-merization step. The unitigs defining the patterns involved in the final model are visualized within their neighborhood in the cDBG, which represents their genomic environment and hence provides insight on the type of variant involved, typically a plasmid-based acquired gene vs a local mutation (single-nucleotide polymorphism [SNP] or indel) in a chromosomal region.

### Evaluation of the computational requirements

We evaluate the computational requirements of the standard lasso and cluster-lasso procedures by measuring the time and memory required to compute a regularization path involving 100 values of the regularization parameter. For the standard lasso, this simply amounts to calling the glmnet function of the glmnetR package, using the variant matrix provided by DBGWAS. For the cluster-lasso procedure, this amounts to:

- making the same call to glmnet to identify the set of active variables,
- computing the *p_a_* × *p* correlation matrix **G** to identify the set of extended features,
- building the clusters of correlated variables, and
- making a second call to glmnet, using the variant matrix defined at the cluster level.

This procedure is repeated 5 times for each drug, using a single Xeon E5-2690-V3 CPU.

## Results

### Cross-validation results

Table 2 provides the results obtained in terms of cross-validation performance and support size of the models. The predictive performance is measured by the area under the ROC curve (AUC) and bACC. Additional performance indicators are provided in Supplementary Table S1. The support size of a model is defined as the number of features it involves, which, respectively, corresponds to individual or clusters of DBGWAS patterns, for the lasso and cluster-lasso strategies. We also report the overall number of unitigs involved, which is only slightly higher than the number of features for the lasso and corresponds to unitigs in total LD. In contrast, this overall number is markedly higher for the cluster-lasso strategy, because of the pattern clustering.

**Table 2:** Cross-validation results

Antibiotic	Lasso				Cluster-lasso			
	bACC	AUC	Support	Unitigs	bACC	AUC	Support	Unitigs
Amikacin	92.7	95.4	16	22 (4)	92.3	95.7	11	93 (36)
Aztreonam	76.7	81.9	31	45 (3)	76.9	82.3	28	425 (125)
Cefepime	74.0	80.4	53	65 (3)	73.6	79.8	34	385 (111)
Cefoxitin	82.4	88.7	134	155 (5)	82.2	88.6	171	1,052 (221)
Ceftazidime	91.6	95.8	51	69 (5)	90.7	95.3	43	863 (185)
Ciprofloxacin	95.6	98.6	25	27 (2)	95.5	98.6	35	422 (139)
Imipenem	93.1	93.6	10	10 (1)	92.7	93.4	7	241 (194)
Meropenem	91.7	94.0	8	8 (1)	91.4	93.5	3	164 (159)
Piperacillin/tazobactam	81.6	89.6	127	144 (4)	81.5	89.0	120	1,220 (226)
Tetracycline	83.0	88.5	181	198 (3)	82.9	87.7	109	640 (104)

This table summarizes the cross-validation results obtained by the lasso and cluster-lasso strategies for the 10 antibiotics, in terms of balanced accuracy (bACC), AUC, support size, overall number of unitigs involved, and maximal number of unitigs associated with a single pattern or cluster (in parentheses).

Both strategies show similar performance in terms of both bACC and AUC, confirming that taking into account, or not, the correlation between features has a limited impact in terms of predictive performance. We also note that the model support is often slightly smaller with cluster-lasso (for 8 of 10 drugs), suggesting that several features selected separately with the lasso ended up merged in a single cluster by the cluster-lasso. As expected, the overall number of unitigs involved in a cluster-lasso model is significantly larger. Interestingly, it is not evenly distributed across its features. In the meropenem model, for instance, 159 of the 164 unitigs defining the model features are associated with a single feature, suggesting that it corresponds to the presence of a gene, as confirmed in the interpretation analysis depicted in the next section.

**Figure 3:**
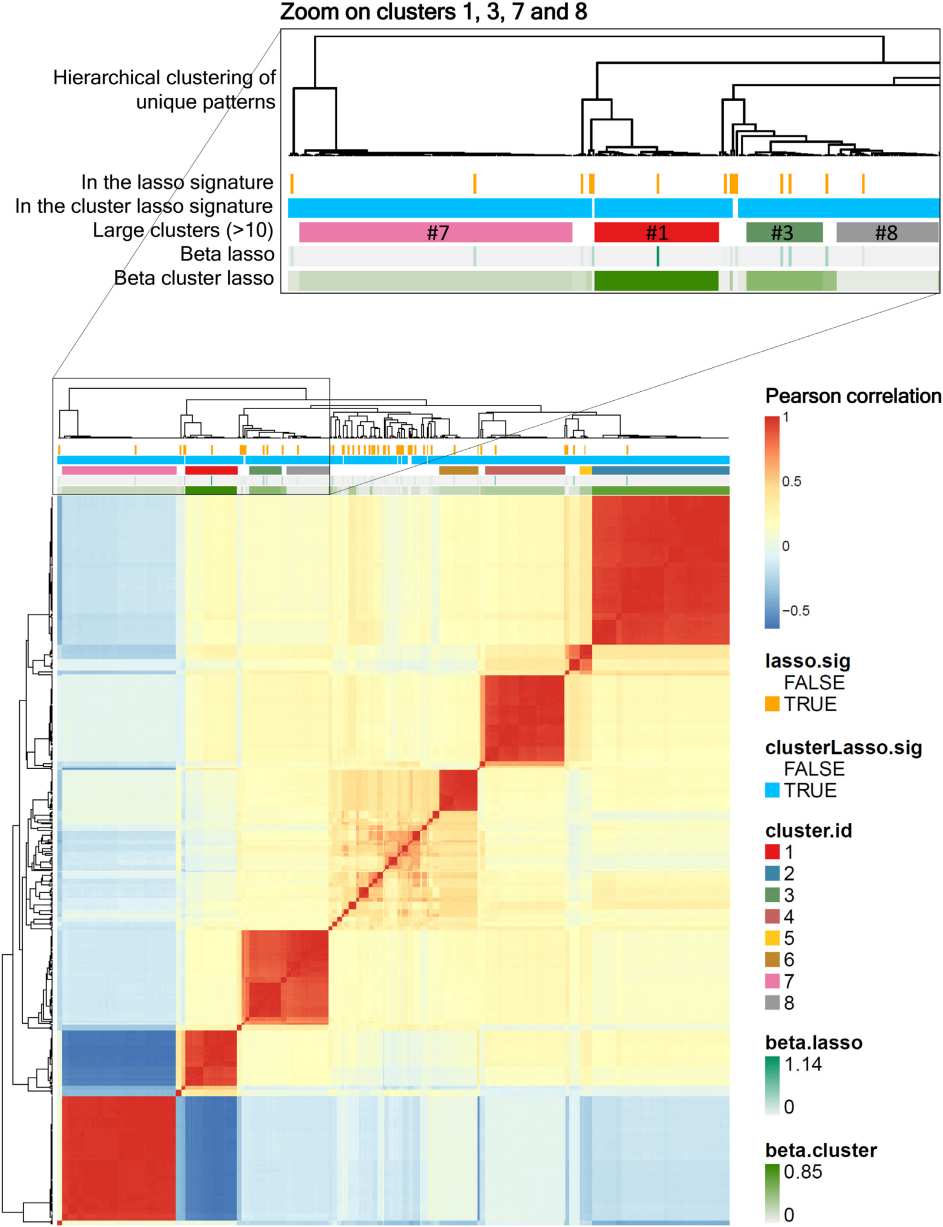
Correlation within features selected in the signatures. This heat map shows the correlation matrix built from the features selected by the lasso and the cluster-lasso (identified by the orange and blue bars shown above the heat map, respectively) for ceftazidime. The corresponding values of model coefficients are represented by green bars. The 8 major clusters (involving >10 patterns) of the cluster-lasso signatures are identified by a dedicated color ranging from red to grey. A zoom of the top left side of the figure allows a better reading of the colored bars for the major clusters 1, 3, 7, and 8.

Finally, Fig. 3 provides a graphical representation of the lasso and cluster-lasso signatures obtained for ceftazidime, which are of moderate complexity. The heat map shows the correlation between the patterns involved in one signature and/or the other, and highlights the 8 major clusters identified by the cluster-lasso strategy (clusters including >10 patterns). While all the patterns defining a cluster have by construction a similar level of predictive power, the lasso model usually selected a single one of them. There is an exception for the third cluster, shown in green in the zoomed area of Fig. 3, where 2 patterns were selected as distinct features of the lasso model.

By explicitly reconstructing and providing these clusters of correlated features to the learning algorithm, the cluster-lasso strategy leads to a more meaningful characterization of the genetic determinants involved, as we describe below.

### Model interpretation

We focus on 2 drugs to illustrate the improved interpretability offered by cluster-lasso signatures: meropenem, where the interpretation is straightforward; and cefoxitin, which is among the signatures of highest support. Additional results obtained for the remaining drugs are deferred to Supplementary Section S5.

**Figure 4:**
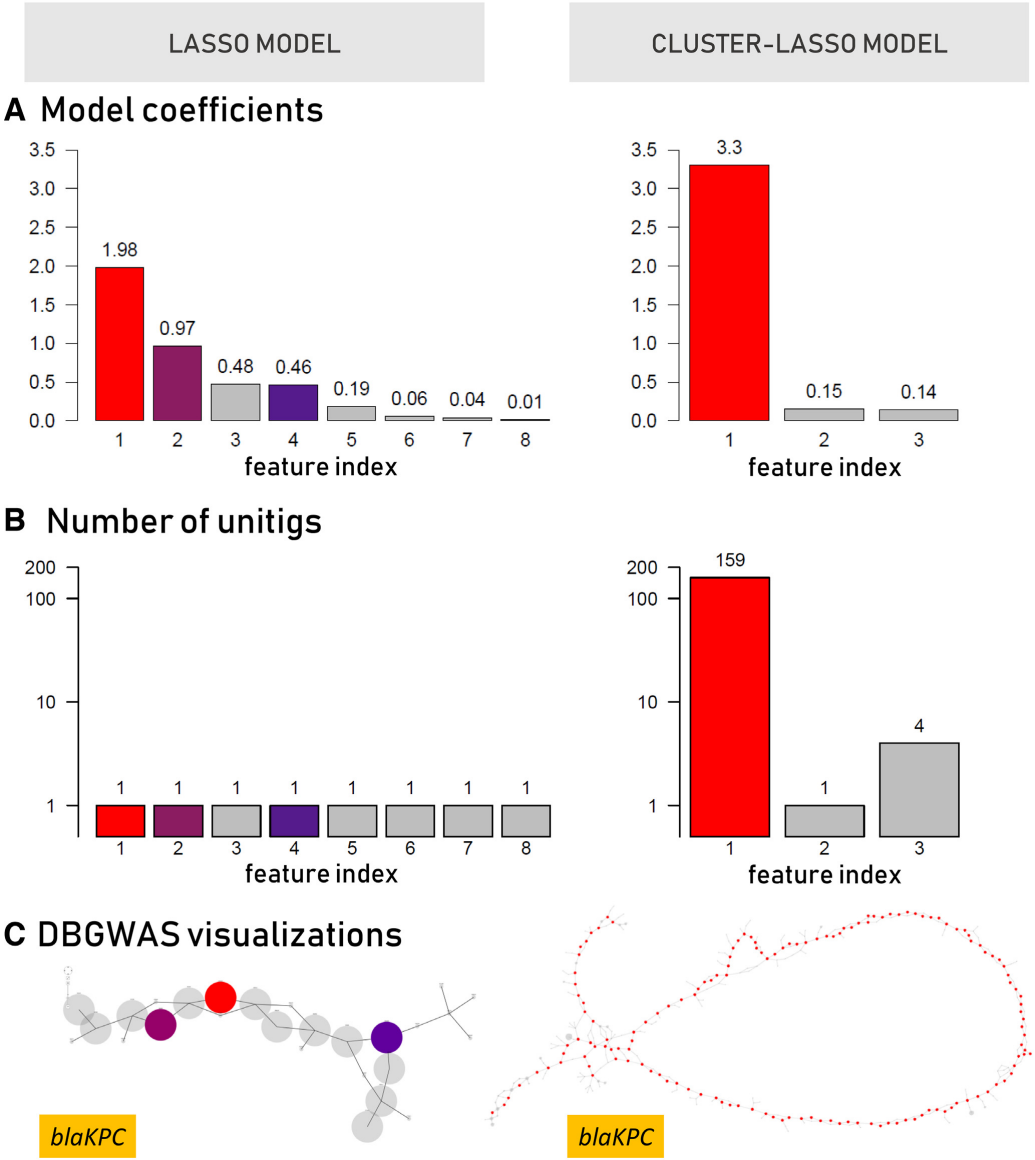
Interpretation of the meropenem signatures. This figure provides a detailed comparison of the lasso (left) and cluster-lasso (right) signatures. (A) Absolute value of the coefficients of the models. (B) Number of unitigs involved in the features of the models. (C) Visualization of the first subgraph obtained by DBGWAS for each signature. Nodes of the graphs correspond to unitigs of the cDBG built by DBGWAS from the training panel of genomes, as illustrated in Fig. 1 and detailed in [25]. Colors identify which unitigs of the graphs in (C) are related to which features of the models in (A) and (B).

As shown in Table 2, the lasso and cluster-lasso meropenem models involve 8 and 3 features, respectively. As shown in Fig. 4B, each lasso feature corresponds to a single unitig, while the cluster-lasso signature involves a large cluster of unitigs (159 of the 164 involved). Fig. 4A shows the magnitude of the model coefficients. It reveals that the cluster-lasso signature is essentially driven by a single prominent feature, while 4–5 features of the lasso signature have a non-negligible weight. The major feature of the cluster-lasso signature corresponds to the large cluster of correlated patterns, and the DBGWAS visualization (Fig. 4C) shows that the corresponding unitigs are organized as a long linear path in the cDBG. This suggests that this cluster corresponds to an entire gene. The annotation provided by DBGWAS shows the gene to be the Class A β-lactamase *bla*_KPC_. The DBGWAS visualization obtained for the lasso signature indicates that 3 of the 8 features—features 1, 2, and 4—are also co-located in a region of the cDBG annotated as *bla*_KPC_. The fact that the lasso selected these specific unitigs within the *bla*_KPC_ gene suggests that the resistance determinants involved are SNPs or indels. While the gene-level annotation is the same as that obtained with the cluster-lasso, the interpretation of the signature in terms of genetic variants is therefore radically different. A closer look at the lasso signature reveals that the 3 *bla*_KPC_ features are actually strongly correlated: they are often observed together. Unsurprisingly, they belong to the largest cluster involved in the cluster-lasso signature, and interestingly, their cumulative weight is approximately equal to that of the cluster-lasso feature (3.4 instead of 3.3). By explicitly detecting that these features are correlated, and merging them into a single feature, together with additional correlated features not even involved in the lasso signature, the cluster-lasso leads to a more meaningful interpretation of the underlying prediction model, in 2 aspects. First, it captures the true nature of the genomic determinant involved: the presence of the *bla*_KPC_ gene, as opposed to mutations within the gene. Second, it assesses the overall contribution of the gene presence in the decision rule, while, in the lasso signature, this contribution is shared by several distinct yet correlated features.

**Figure 5:**
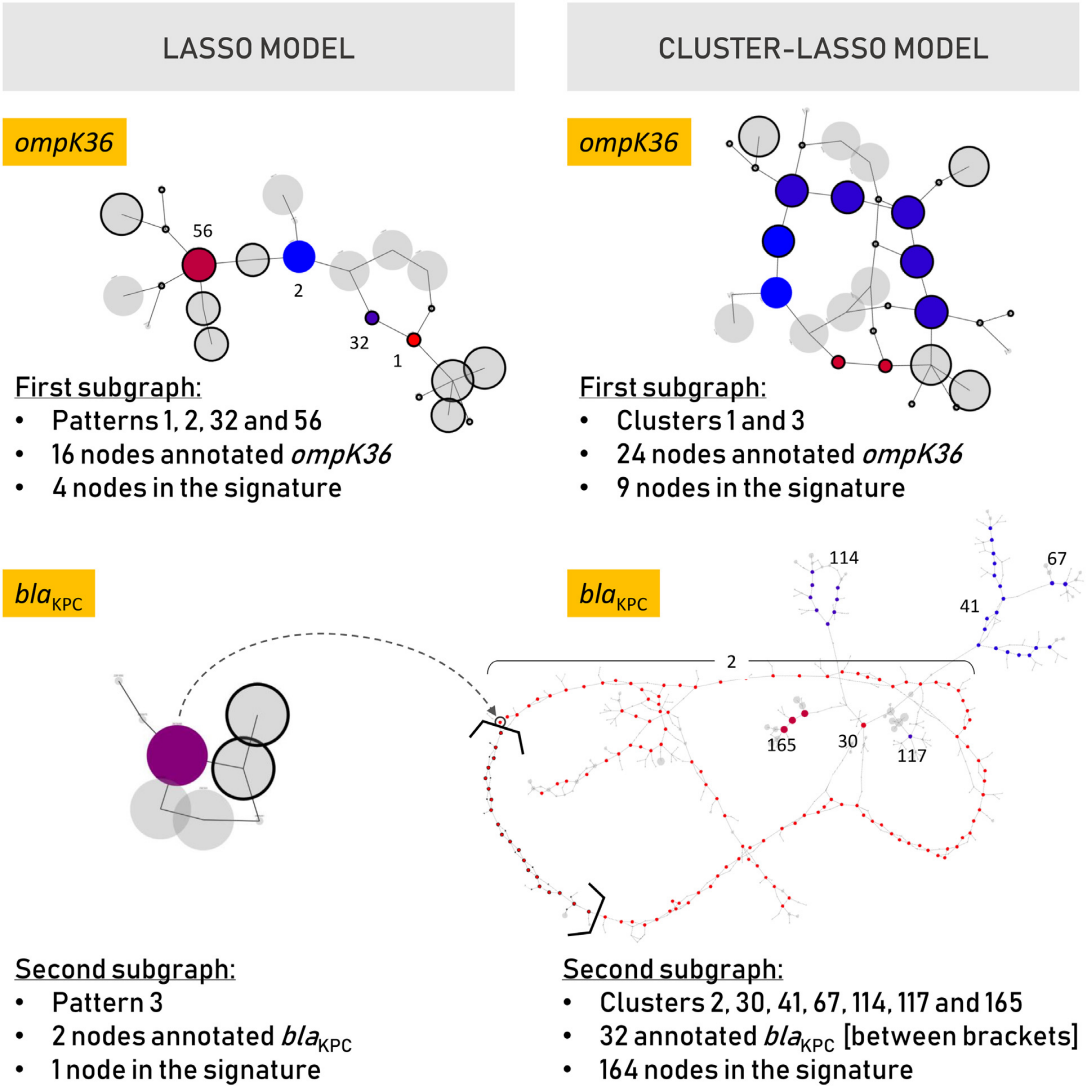
DBGWAS visualizations for the interpretation of the cefoxitin signatures. This figure presents the 2 first subgraphs obtained by DBGWAS for the lasso and cluster-lasso signatures. The DBGWAS subgraphs are ordered by decreasing maximal absolute value of model coefficient among all patterns/clusters involved in the subgraph. Likewise, pattern and cluster identifiers are ordered by decreasing absolute value of model coefficient, meaning for instance that Pattern/cluster 1 has a greater weight in the model than Pattern/cluster 2. The nodes (unitigs) belonging to patterns/clusters of the signatures are colored by the value of their model coefficients (from blue to red, indicating negative and positive values, respectively). The grey nodes/unitigs, not involved in the models, represent their genomic neighborhood. The nodes for which an annotation related to antibiotic resistance was found are surrounded by a black circle. Bold brackets are used in the bottom right subgraph to highlight these black-circled nodes. This particular subgraph gathers 7 clusters, whose identifiers are reported in the picture. Cluster 2 is the largest one and includes the *bla*_KPC_-annotated nodes. The dashed arrow shows which node of the cluster-lasso *bla*_KPC_ subgraph corresponds to the one selected by the lasso.

Likewise, Fig. 5 presents the DBGWAS analysis of the lasso and cluster-lasso signatures obtained for cefoxitin. We focused on the 2 first subgraphs provided by the software, which represent the 2 genomic neighborhoods of the most important patterns or clusters of patterns involved in the models. The subgraphs are indeed ordered according to the maximal absolute value of model coefficients among all patterns or clusters involved in the subgraph. While DBGWAS identifies the same resistance genes in both methods (the efflux pump *ompK36* and *bla*_KPC_), the nature of the underlying resistance determinants cannot be deduced from the lasso signature. The *ompK36*-annotated subgraph obtained for the cluster-lasso signature (top right panel of Fig. 5) involves 2 clusters gathering 9 unitigs (clusters 1 and 3) and presents a topology attributable to a local polymorphism: a complex bubble, with a fork separating susceptible (blue) and resistant (red) strains, as described in [25]. The corresponding lasso subgraph, shown in the top left panel, includes 4 patterns (Patterns 1, 2, 32, and 56) each having its proper value of model coefficient, represented by 4 shades of colors ranging from blue to red. These distinct model coefficient values can lead to wrong conclusions regarding the individual importance of the corresponding unitig sequences. Indeed, aligning these unitigs with annotated *ompK36* sequences reveals that Features 2 and 56 both represent the wild type, while Features 1 and 32 align to the insertion of 2 amino acids in the L3 loop, as described in Novais et al. [35] (Supplementary Fig. S7). The second lasso subgraph (bottom left panel of Fig. 5) includes a single feature of the signature (shown in purple), surrounded by 7 nodes (shown in grey), among which 2 are annotated as *bla*_KPC_. The node of the signature is however not annotated itself; hence the subgraph could be interpreted as a local polymorphism in the promoter region of the *bla*_KPC_ gene. The cluster-lasso subgraph shown in the bottom right panel reveals however that this unitig was selected by the lasso among hundreds of highly correlated unitigs. They all belong to Cluster 2, which includes the complete *bla*_KPC_ gene (shown in brackets) and plasmid sequences in strong LD.

By its ability to leverage correlations between patterns, the cluster-lasso approach allowed us to identify that the second causal determinant involved in the cefoxitin model is the *bla*_KPC_ gene, which was brought by a plasmid. As was the case for meropenem, it offers a far better interpretability than the lasso, which did not even explicitly identify *bla*_KPC_ in its features, but only a specific sequence of its direct plasmidic environment. We emphasize however that this improved interpretability may have a price in terms of predictive performance. Indeed, correlations between genomic features may be overestimated if the training dataset is not diverse enough, which may lead to the reconstruction of oversized clusters. This may for instance be the case here of the second cluster-lasso cluster, which identified *bla*_KPC_ within a specific plasmid, while *bla*_KPC_ is known to jump frequently between plasmids [36], many of which may not have been observed in the training set. Applying this model to a strain harboring *bla*_KPC_ in a different plasmidic environment may therefore fail to activate a sufficient number of patterns of this cluster, which may prevent recognizing the strain as resistant.

### Performance on the test set

Table 3 shows the predictive performance obtained on the test set by the lasso and cluster-lasso signatures, in terms of sensitivity, specificity, bACC, and AUC.

**Table 3:** Test set results

Antibiotic	Lasso				Cluster-lasso			
	Sensitivity	Specificity	bACC	AUC	Sensitivity	Specificity	bACC	AUC
Amikacin	84.3	74.4	79.3	86.0	77.0	80.0	78.5	86.4
Aztreonam	69.6	80.0	74.8	83.3	67.2	80.0	73.6	82.0
Cefepime	77.0	60.4	68.7	69.1	78.3	54.7	66.5	69.6
Cefoxitin	51.7	92.8	72.2	74.6	55.2	94.2	74.7	76.0
Ceftazidime	77.7	98.4	88.1	94.3	60.8	96.0	78.4	92.8
Ciprofloxacin	91.3	91.2	91.2	96.6	92.1	89.8	90.9	96.7
Imipenem	65.3	99.0	82.2	87.2	65.6	98.3	81.9	85.4
Meropenem	66.0	97.7	81.8	81.1	66.3	97.7	82.0	78.8
Piperacillin/tazobactam	63.1	82.9	73.0	82.7	58.9	87.7	73.3	81.6
Tetracycline	64.8	93.5	79.2	82.4	64.5	94.8	79.7	82.8

This table summarizes the results obtained on the test dataset by the lasso and cluster-lasso models for the 10 antibiotics, in terms of sensitivity, specificity, balanced accuracy (bACC), and AUC.

**Figure 6:**
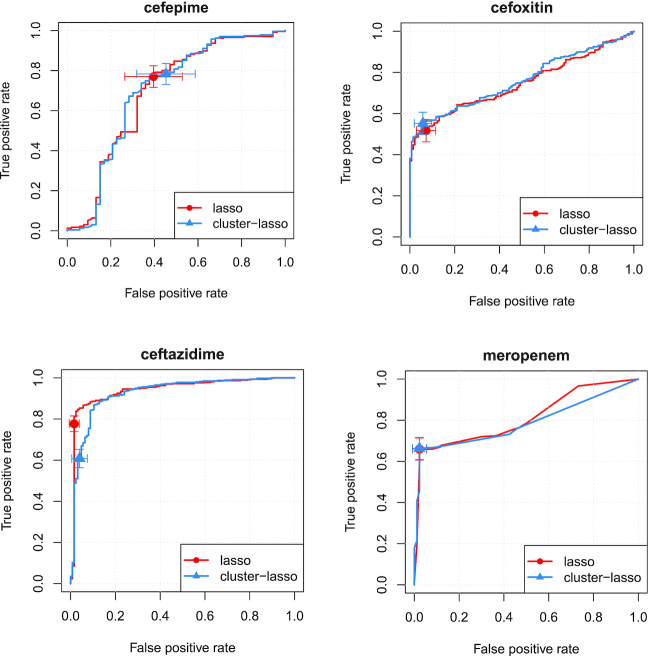
Test set results. This figure represents the ROC curves obtained for cefepime, cefoxitin, ceftazidime, and meropenem by the lasso (red) and cluster-lasso (blue) signatures, as well as their associated sensitivities and specificities, with their 95% confidence intervals.

We first noted that the lasso and cluster-lasso strategies reached a similar level of bACC for most drugs, although they did not always achieve the same trade-off in terms of sensitivity and specificity. We noted however that the confidence intervals of the corresponding sensitivities and specificities largely overlapped for all drugs but ceftazidime (Fig. 6 and Supplementary Fig. S8), indicating that they were not significantly different between lasso and cluster-lasso, except for 1 drug.

**Figure 7:**
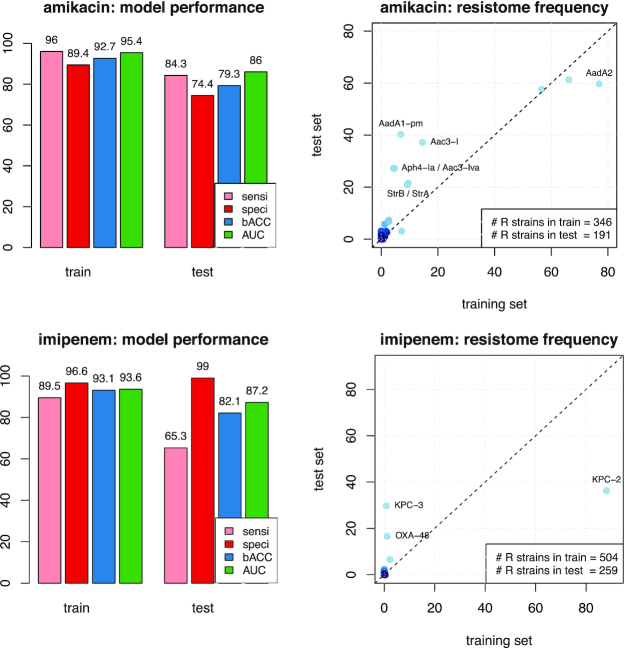
Resistome analysis. This figure compares the training and test panels of genomes in terms of predictive performance and resistome constitution for the drugs amikacin (top) and imipenem (bottom). Left: Predictive performance in terms of sensitivity (sensi), specificity (speci), bACC, and AUC estimated by cross-validation on the training set and measured on the test set, using the lasso signatures. Right: Comparison of the resistome constitutions. Each kleborate resistance marker is represented by its prevalence in the resistant strains of the training (*x*-axis) and test (*y*-axis) panels.

We often observed a serious drop between the predictive performance estimated by cross-validation and that observed for the test set: >5 points of bACC for 6 of 10 drugs, and up to 10 points or more for amikacin, cefoxitin, imipenem, and meropenem (13.4, 10.2, 10.9, and 9.9 points, respectively). This suggested that the training dataset taken from Nguyen et al. [8] could not account for the entire diversity displayed by *K. pneumoniae*. A simple analysis of the strain's resistomes and sequence types (ST) using the kleborate software [37] revealed that the prevalence of several STs and well-known resistance genes was sometimes very different in the 2 panels. This latter point is illustrated in Fig. 7 for amikacin and imipenem, which had the largest decrease in performance. Supplementary Section S1 (Fig. S3) shows the difference in the ST prevalence, highlighting that the training set involves 2 main STs (ST307 and ST258), which have a much lesser prevalence in the test dataset. Redesigning the training and test datasets by shuffling the original ones to obtain a homogeneous split fixed this generalization issue (Supplementary Section S7). This illustrates that while ML models can indeed succeed in learning accurate prediction rules, they fail to generalize when the dataset on which they are trained does not account for the overall diversity of the bacterial species.

Finally, Table 3 and Supplementary Fig. S9 show an uneven level of prediction performance among the 10 antibiotics considered. The best performances were obtained for ciprofloxacin and ceftazidime, with an AUC ~95% using either the original or the redesigned datasets (Supplementary Fig. S9). The poorest performances were obtained for 2 β-lactams: cefepime, a fourth-generation cephalosporin; and the monobactam aztreonam. This may be due to a reduced penetrance of their genetic determinants, as described in human genetics [38], because more complex resistance mechanisms are involved, including efflux pumps, gene regulation, or plasmid copy number [39–41].

### Computational requirements

**Figure 8:**
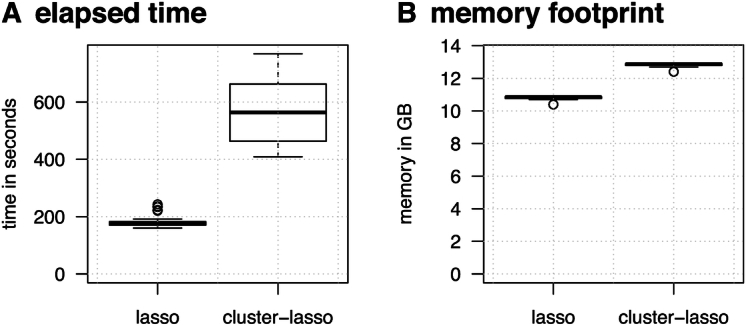
Time and memory requirements. The box plots represent the variability of the time in seconds (A) and maximum memory in gigabytes (GB) (B) required to generate a lasso or cluster-lasso regularization path for the 10 antibiotics.

Fig. 8 indicates that while the duration of the cluster-lasso was on average ~3 times longer than the lasso (571 vs 180 seconds), it took only ~10 minutes to obtain an entire regularization path defined at the cluster level. Optimizing the regularization parameter using our cross-validation process therefore took ~5 hours on a single CPU. We noted that while the time required by the lasso was relatively homogeneous across drugs, it was more variable for the cluster-lasso. This variability was due to the fact that the lasso used in the first step identified a variable number of active features, which directly affected the time required to screen the remaining ones. This is illustrated in Supplementary Fig. S10.

In terms of memory, we noted that the cluster-lasso procedure led to an overhead of ~2 GB with respect to the lasso, which was related to the computation of the correlation matrix **G**. In practice, we limited this overhead by computing this matrix by slices, considering subsets of *p*′ = 10,000 features and computing *p_a_* × *p*′ matrices instead of the entire *p_a_* × *p* matrix at once. Altogether, this led to a computationally efficient procedure, allowing cluster-level signatures to be identified in a few hours, for a limited memory footprint. We note that it could be straightforwardly parallelized, using several CPUs to compute the various slices of the correlation matrix **G**.

## Discussion

Representing bacterial genomes using *k*-mers leads to very high-dimensional representations with strong correlation structures. This may hinder a meaningful interpretation of predictive models built by sparse ML strategies like lasso-penalized regressions [42] or algorithms based on decision trees [43], which are known to be unstable in this case: when some features are strongly correlated, they tend to select 1, a or few of them, arbitrarily [44]. This instability may not be an issue in terms of predictive performance: as long as 1 feature among a group of correlated ones appears in the model, the prediction may be unchanged. It may however have a severe impact in terms of interpretability because the features selected by the model may provide an incomplete or erroneous characterization of the causal resistance determinant.

We propose a simple and computationally efficient strategy to cope with the strong correlation structures inherent to *k*-mer–based representations, and build sparse and meaningful genomic signatures. While performing a systematic study on thousands of strains of *K. pneumoniae*, our approach compared favorably to the state of the art, providing indeed a comparable level of performance, while offering a greater interpretability of the genomic features involved in the models. On this challenging genetically flexible bacterial species with significant accessory genome components, this new approach allowed meaningful scientific insights to be extracted from the identified signatures, as further detailed in Section S5 of the Supplementary Materials.

Central to our approach is a 3-step strategy, where a sparse ML algorithm is first used to screen features in a generic manner, which are then extended to clusters of strongly correlated features, ultimately considered as candidate features to be included in the final antibiotic resistance prediction model.

In this work both the screening and final learning stages were based on the lasso-penalized logistic regression model, which is appealing in this context for several reasons. First and foremost, it has been shown to be competitive with alternative ML algorithms in several articles (e.g., [10, 17, 26, 45]). The underlying model is moreover easy to interpret because it combines several genomic determinants in a global probabilistic model with weights modulating their respective effects, hence reflecting the fact that they can be associated with different levels of resistance. Last but not least, the R package glmnet offers a very efficient implementation, scaling gracefully to large datasets like the one involved in this study, as shown in Fig. 8. The principle of our method is nevertheless generic and could readily be transposed to other sparse ML algorithms, such as xgboost [4, 8] or set cover machines [26]. Likewise, it could straightforwardly be extended to handle MICs or other phenotypic traits, as well as other types of genomic features (e.g., relying on SNPs instead of *k*-mers).

Several alternative strategies could be considered to handle correlations between *k*-mers. Most related to our approach are the elastic-net and the group-lasso strategies, which also rely on logistic regression—and more generally on generalized linear models—but with alternative regularization penalties. The elastic-net penalty combines the lasso and the ridge penalties, which leads to sparse models with a grouping mechanism: correlated features tend to be selected together [46]. This approach was recently shown to be efficient in the context of bacterial genome-wide association studies (GWAS), providing increased statistical power for the identification of genotype-phenotype associations and accurate prediction rules [47]. As we demonstrate in Supplementary Section S9, however, it remains limited in its ability to provide interpretable predictive signatures, for several reasons. First, while it has the effect of stabilizing the lasso solution and of simultaneously activating groups of correlated features, these groups are not defined explicitly, which intrinsically makes the interpretation of the model difficult. Moreover, while the parameter controlling the trade-off between the lasso and ridge penalties had a direct effect on the number of selected features, it had little effect on the predictive performance of the model, thereby making it difficult to optimize objectively. Finally, we empirically observed that it led to a partial and heterogeneous reconstruction of the genomic features obtained by the cluster-lasso: a significant fraction of the cluster members were not selected by the elastic-net, and the individual weights associated to the selected ones greatly varied, although their level of predictive power was comparable.

The group-lasso penalty leverages pre-defined groups of features, ensuring that all features of a given group are either active or inactive simultaneously [48]. This strategy was for instance considered in human GWAS, using groups of SNPs defined spatially to account for their LD [49]. Transposing this idea to bacterial genomes is challenging because no such prior information is available to define groups, as LD can be genome-wide [29]. A solution could be to identify clusters of correlated *k*-mers using agglomerating strategies [31] but is hard to carry out in practice from the high-dimensional datasets involving 10^5^−10^6^ features encountered in our application.

Our approach can therefore be seen as a simple and efficient strategy to approximate such a group-lasso process in very high-dimensional settings. Instead of collapsing groups of correlated features into composite variables, a natural extension of our method would however be to rely on a group-lasso penalized regression defined at the cluster level. Each feature would then be granted its own weight, which could allow their individual predictive power to be better reflected. We empirically observed that the weight variability within a cluster was very small, as shown in Supplementary Fig. S14, which therefore indicated that keeping the features separated or averaging them is essentially equivalent. In practice, we find it easier to explicitly collapse each cluster to a single composite variable to interpret the model parameters.

On the practical side, our method involves 2 hyper-parameters, besides the regularization parameter, to identify active variables and to build the final model. Although these so-called screening and clustering thresholds did not have a strong influence in this study (Supplementary Section S2), they may be cumbersome to optimize in practice for other applications. A natural extension to our method would be to consider re-sampling strategies in the clustering step, in order to identify stable clusters, whose constitution would be robust to the precise definition of the clustering threshold [50]. Alternatively, one could rely on tree-guided lasso penalization to leverage the entire dendrogram during the final learning step, which would then simultaneously identify clusters and learn the prediction model [51].

Regarding AMR prediction, our study performed on *K. pneumoniae* confirms several observations made recently, namely, that *k*-mer–based approaches can learn sparse prediction rules without any prior information and that the level of predictive performance can vary by antibiotic [26, 28]. Importantly, our study involved a novel panel of 634 *K. pneumoniae* strains for the validation of the prediction models and suggested that the problem is more challenging than reported in Nguyen et al. [8]. The results they reported were indeed probably optimistic because the genome panel they considered did not account for the overall genomic diversity of *K. pneumoniae* as a species because it involved 2 over-represented STs (ST307 and ST258) representing 60% of the isolates (Supplementary Fig. S3). The 634 additional strains with genomes and phenotypes considered in this study will help in learning more accurate and generalizable prediction models, as suggested by the preliminary experiments described in Supplementary Section S7. Another limitation of the present study lies in the fact that the phenotypic AST methods used to define the reference MICs differed between the training set (which involved the Phoenix technology [Becton Dickinson, Franklin Lakes, NJ, USA] only) and the test set (which was based on agar dilution, broth microdilution, or VITEK 2). Indeed, AST is notoriously subject to a high level of technical variability [52], which intrinsically brings noise to the reference labels used to train and validate supervised ML models. A natural question therefore arises whether an ML model learned from data provided by a given AST method will generalize to data provided by a different AST method. A dedicated study described in Supplementary Section S11 suggests that this issue was not critical on this dataset, which therefore suggests that the lack of generalization observed on the test set is mainly driven by its genomic heterogeneity with respect to the training set.

Finally, the ML methods developed in this study are available in a generic R package that can be easily transposed to other bacterial species, as shown in Supplementary Section S12, and even other applications, not necessarily involving *k*-mers or AMR phenotypes. On the challenging dataset considered in this study, involving >1,000 strains for >1,000,000 genomic features, the computational requirements remained limited and the signatures could be identified in a few hours on a standard workstation. Coupled with the enriched level of intepretability they offer, we believe that our approach will help define prediction models amenable to routine diagnostics.

## Availability of Source Code and Requirements

- Project name: Cluster Lasso
- Project home page: https://gitlab.com/biomerieux-data-science/clustlasso
- Operating system: Unix
- License: GNU GPL-v2
- RRID:SCR_018820
- bioTools ID: clustlasso
- Step-by-step procedure for using clustlasso package available at: https://gitlab.com/biomerieux-data-science/clustlasso-dbgwas-integration

## Availability of Supporting Data and Materials

All the genomes and associated phenotypes involved in this study are publicly available (data provided in the Supplementary Material, in genomes_info.csv). An archival copy of the code and supporting data is available via the *GigaScience* repository, GigaDB [53].

## Additional Files

Supplementary Section S1. Dataset constitution.

Supplementary Section S2. Impact of screening and clustering thresholds.

Supplementary Section S3. Cross-validation process of model selection.

Supplementary Section S4. Detailed predictive performance.

Supplementary Section S5. Interpretation of the models.

Supplementary Section S6. ROC curves.

Supplementary Section S7. Re-designing the dataset to evaluate the generalization ability of the models.

Supplementary Section S8. Time and memory evaluation.

Supplementary Section S9. Evaluation of an elastic-net stategy.

Supplementary Section S10. Evaluation of a cluster-level group-lasso stategy.

Supplementary Section S11. Impact of AST method on generalization.

Supplementary Section S12. Results obtained on other species.

Supplementary Figure S1. Number of genomes available per country.

Supplementary Figure S2. Number of genomes available per source, vs country and antibiotic susceptibility method.

Supplementary Figure S3. Number of genomes available per ST.

Supplementary Figure S4. Cross-validation results - impact of considering different screening and clustering thresholds.

Supplementary Figure S5. Illustration of the cross-validation process.

Supplementary Figure S6. Cluster-lasso signatures: annotation and interpretation.

Supplementary Figure S7. Multiple alignment of the unitigs annotated as ompK36 in the cefoxitin signature.

Supplementary Figure S8. Test set results - ROC curves obtained for amikacin, aztreonam, ciprofloxacin, imipenem, piperacillin-tazobactam and tetracycline.

Supplementary Figure S9. Test versus cross-validation performance using the original and the re-designed datasets.

Supplementary Figure S10. Time and memory requirements for the lasso and cluster-lasso procedures.

Supplementary Figure S11. Cross-validation results based on the elastic-net penalty.

Supplementary Figure S12. DBGWAS visualization of the main genomic feature identified in the meropenem signatures obtained using the lasso, elastic-net, and cluster-lasso strategies.

Supplementary Figure S13. Correlation of selected features in the meropenem and ceftazidime signatures obtained using the elastic-net penalty.

Supplementary Figure S14. Cluster-level group-lasso models obtained for the drugs amikacin, cefepime, ceftazidime and meropenem.

Supplementary Figure S15. Impact of AST method on prediction performance - distribution of closest distance to the training distance.

Supplementary Figure S16 Impact of AST method on prediction performance - detailed results for cefoxitin and meropenem.

Supplementary Table S1. Cross-validation results - summary of performance.

Supplementary Table S2. Impact of AST method on prediction performance - summary table.

Supplementary Table S3. Cross-validation results on other species.

supplementary-data.pdf: pdf file gathering supplementary analyses, figures and tables described above.

genomes_info.csv: csv file containing genome accessions and phenotypes.

KPN_SNS-cl_lasso-annot_signatures.xlsx: xls file describing the annotations of the cluster-lasso signatures.

giaa110_GIGA-D-20-00079_Original_Submission

giaa110_GIGA-D-20-00079_Revision_1

giaa110_GIGA-D-20-00079_Revision_2

giaa110_Response_to_Reviewer_Comments_Original_Submission

giaa110_Response_to_Reviewer_Comments_Revision_1

giaa110_Reviewer_1_Report_Original_Submission Nicole Wheeler -- 4/14/2020 Reviewed

giaa110_Reviewer_1_Report_Revision_1 Nicole Wheeler -- 8/3/2020 Reviewed

giaa110_Reviewer_1_Report_Revision_2 Nicole Wheeler -- 9/14/2020 Reviewed

giaa110_Reviewer_2_Report_Original_Submission James J. Davis -- 5/2/2020 Reviewed

giaa110_Reviewer_2_Report_Revision_1 James J. Davis -- 8/3/2020 Reviewed

giaa110_Supplemental_Files

## Abbreviations

ACL: adaptive cluster lasso; AMR: antimicrobial resistance; AST: antibiotic susceptibility testing; AUC: area under the (ROC) curve; bACC: balanced accuracy; cDBG: compacted de Bruijn graph; CPU: central processing unit; GWAS: genome-wide association study; LD: linkage disequilibrium; MAF: minor allele frequency; MIC: minimum inhibitory concentration; ML: machine learning; NCBI: National Center for Biotechnology Information; NS: non-susceptible; PATRIC: Pathosystems Resource Integration Center; ROC: receiver operating characteristic; S: susceptible; SNP: single-nucleotide polymorphism; ST: sequence type.

## Competing Interests

All authors are employees of bioMérieux, a company creating and developing infectious disease diagnostics. The authors declare that they have no other competing interests.

## Funding

The work performed by M.P. was funded by the European Union's Horizon 2020 research and innovation program under the Marie Skłodowska-Curie Grant Agreement No. 675412 (New Diagnostics for Infectious Diseases [ND4ID]).

## Authors' Contributions

P.M. and M.J. conceived and designed the experiments, performed the experiments, analyzed the data, authored or reviewed drafts of the manuscript, and approved the final draft. M.P. collected and prepared the test dataset, carried out the resistome analysis, authored or reviewed drafts of the manuscript, and approved the final draft. A.v.B. authored or reviewed drafts of the manuscript and approved the final draft.

## Acknowledgements

We thank Professor Herman Goossens for supporting M.P.
