# Supplementary material for: Interpreting k-mer–based signatures for antibiotic resistance prediction
Source: Gigascience. 2020 Oct 17;9(10):giaa110. doi: 10.1093/gigascience/giaa110 (PMC7568433; doi:10.1093/gigascience/giaa110)
Supplement: giaa110_GIGA-D-20-00079_Revision_1 [file giaa110_giga-d-20-00079_revision_1.pdf]

GigaScience

Interpreting k-mer-based signatures for antibiotic resistance prediction

--Manuscript Draft--

**Manuscript Number:**	GIGA-D-20-00079R1
**Full Title:**	Interpreting k-mer-based signatures for antibiotic resistance prediction
**Article Type:**	Research
**Funding Information:**	Horizon 2020 () (675412) — MSc. Mattia Palmieri
**Abstract:**	Background . Recent years witnessed the development of several k -mer-based approaches aiming to predict phenotypic traits of bacteria based on their whole-genome sequences. While often convincing in terms of predictive performance, the underlying models are in general not straightforward to interpret, the interplay between the actual genetic determinant and its translation as k -mers being generally hard to decipher.<br>Results . We propose a simple and computationally efficient strategy allowing one to cope with the high correlation inherent to k -mer-based representations in supervised machine learning models, leading to concise and easily interpretable signatures. We demonstrate the benefit of this approach on the task of predicting the antibiotic resistance profile of a Klebsiella pneumoniae strain from its genome, where our method leads to signatures defined as weighted linear combinations of genetic elements that can easily be identified as genuine antibiotic resistance determinants, with state of the art predictive performance.<br>Conclusions . By enhancing the interpretability of genomic k -mer-based antibiotic resistance prediction models, our approach improves their clinical utility, hence will facilitate their adoption in routine diagnostics by clinicians and microbiologists. While antibiotic resistance was the motivating application, the method is generic and can be transposed to any other bacterial trait. A R package implementing our method is available at https://gitlab.com/biomerieux-data-science/clustlasso.
**Corresponding Author:**	Magali Jaillard Dancette<br>bioMerieux SA<br>Marcy l'Etoile, FRANCE
**Corresponding Author Secondary Information:**	
**Corresponding Author's Institution:**	bioMerieux SA
**Corresponding Author's Secondary Institution:**	
**First Author:**	Magali Jaillard Dancette
**First Author Secondary Information:**	
**Order of Authors:**	Magali Jaillard Dancette<br>Mattia Palmieri<br>Alex van Belkum<br>Pierre Mahé
**Order of Authors Secondary Information:**	
**Response to Reviewers:**	We thank the reviewers for their thoughtful and positive comments, and for their suggestions regarding our manuscript entitled “Interpreting k-mer-based signatures for antibiotic resistance prediction”.<br>You will find attached a file called "answers-to-reviewers.pdf" providing a point-by-point response to these comments, which we believe greatly improve the quality of the manuscript.<br><br>The most important modifications made to the manuscript are the following ones :

Powered by Editorial Manager® and ProduXion Manager® from Aries Systems Corporation

	1. The accompanying R package is now hosted on a dedicated GitLab repository, and we have included additional code to illustrate how to interface this package with DBGWAS, as suggested by Reviewer 1.<br>2. We have removed the comparison with the models of Nguyen et al. (2018). Further discussing this issue with Reviewer 2 led us to realize that the comparison we made was not as fair as we intended, hence we followed the suggestion of the reviewer to discard it.<br>3. We have added a dedicated study in the supplementary materials (Supplementary Section S11) to assess the impact of the phenotypic AST method on the performance of the model, a point that was raised by both reviewers.<br><br>We also attached as supplementary material a second version of the modified manuscript for the reviewers in which the modifications applied to the original manuscript are highlighted in red.<br>We hope that based on these changes our manuscript will now be acceptable for publication in GigaScience.<br><br>Magali Jaillard, on behalf of co-authors
Additional Information:	
**Question**	**Response**
Are you submitting this manuscript to a special series or article collection?	No
**Experimental design and statistics**<br><br>Full details of the experimental design and statistical methods used should be given in the Methods section, as detailed in our Minimum Standards Reporting Checklist. Information essential to interpreting the data presented should be made available in the figure legends.<br><br>Have you included all the information requested in your manuscript?	Yes
**Resources**<br><br>A description of all resources used, including antibodies, cell lines, animals and software tools, with enough information to allow them to be uniquely identified, should be included in the Methods section. Authors are strongly encouraged to cite Research Resource Identifiers (RRIDs) for antibodies, model organisms and tools, where possible.<br><br>Have you included the information requested as detailed in our Minimum	Yes

Standards Reporting Checklist?	
**Availability of data and materials**<br><br>All datasets and code on which the conclusions of the paper rely must be either included in your submission or deposited in publicly available repositories (where available and ethically appropriate), referencing such data using a unique identifier in the references and in the "Availability of Data and Materials" section of your manuscript.<br><br>Have you have met the above requirement as detailed in our Minimum Standards Reporting Checklist?	Yes

Powered by Editorial Manager® and ProduXion Manager® from Aries Systems Corporation

Manuscript main file
Click here to access/download;Manuscript;main.pdf

OXFORD
(GIGA)^n SCIENCE

GigaScience, 2019, 1–12

doi: xx.xxxx/xxxx
Manuscript in Preparation
Paper

PAPER

Interpreting *k*-mer based signatures for antibiotic resistance prediction

Magali Jaillard[1,*], Mattia Palmieri[1], Alex van Belkum[1] and Pierre Mahé[1,*]

[1]bioMérieux, Marcy l'Etoile, France

[*]magali.jaillard-dancette@biomerieux.com,pierre.mahe@biomerieux.com

Abstract

Background Recent years witnessed the development of several *k*-mer-based approaches aiming to predict phenotypic traits of bacteria based on their whole-genome sequences. While often convincing in terms of predictive performance, the underlying models are in general not straightforward to interpret, the interplay between the actual genetic determinant and its translation as *k*-mers being generally hard to decipher.
Results We propose a simple and computationally efficient strategy allowing one to cope with the high correlation inherent to *k*-mer-based representations in supervised machine learning models, leading to concise and easily interpretable signatures. We demonstrate the benefit of this approach on the task of predicting the antibiotic resistance profile of a *Klebsiella pneumoniae* strain from its genome, where our method leads to signatures defined as weighted linear combinations of genetic elements that can easily be identified as genuine antibiotic resistance determinants, with state of the art predictive performance.
Conclusions By enhancing the interpretability of genomic *k*-mer-based antibiotic resistance prediction models, our approach improves their clinical utility, hence will facilitate their adoption in routine diagnostics by clinicians and microbiologists. While antibiotic resistance was the motivating application, the method is generic and can be transposed to any other bacterial trait. A R package implementing our method is available at
https://gitlab.com/biomerieux-data-science/clustlasso.

Key words: Antibiotic resistance; supervised machine learning; *k*-mer; De Bruijn graph

Introduction

Antimicrobial resistance (AMR) is a global healthcare problem and rapid diagnostics are needed to select the right treatment, to follow the route to cure and to monitor and prevent community- and hospital-acquired outbreaks of infections. Next-Generation Sequencing (NGS) is a disruptive technology which is, potentially, able to supplant or even replace the current plethora of diagnostic tests with a single, most probably well-affordable and faster solution. Inferring the antibiotic resistance profile from a bacterial genome is challenging. However, good results have been obtained for several species [1, 2, 3, 4, 5, 6, 7], including *Klebsiella pneumoniae* [8]. Su et al. [9] discussed the challenges of NGS-based antibiotic susceptibility testing (AST) and provided a comprehensive review of the current state of the art in this field.

Early approaches relied on the detection of known resistance markers to claim resistance, a strategy sometimes referred to as *direct association analysis* [10]. While effective when the genetic bases of antibiotic resistance are well known, which is the case for instance for most antibiotic resistance mechanisms in the highly clonal species *M. tuberculosis* [11, 12] and *Salmonella typhi* [13], this approach suffers from several limitations. First and foremost, it intrinsically relies on prior knowledge of the precise nature of the resistance determinants, which may not be available for all species and drugs. Secondly, it is not able to account for the fact that these markers can have different levels of predictive power [14, 15], that they can act in a multi-factorial fashion through epistasis [16, 17], or that resistance can result from the accumulation of several different

Compiled on: July 23, 2020.
Draft manuscript prepared by the author.

mutations [18, 19]. Last but not least, it is hazardous to predict susceptibility when no marker is detected, since the resistance marker may be novel and databases incomplete.

Building AMR prediction models is now more and more addressed from the supervised machine learning (ML) standpoint: given a set of genomes with associated reference phenotypes (provided by phenotypic AST methods [20]), one seeks a prediction rule allowing to infer the resistance or susceptibility of a novel strain from genomic features. While ML methods also suffer from a completeness limitation, as the set of genomes may not be representative of the genomic diversity of the whole species, they have the ability to identify novel markers or markers combinations in situations where no or limited prior knowledge is available, hence are gaining an increasing popularity in this context. Even for *M. tuberculosis*, where the antibiotic resistance knowledge is probably among the most thorough and complete, recent studies showed that performance of direct association strategies can still be significantly improved by ML models [10, 17].

A great variety of ML strategies have been explored, taking into account several parameters. First, regarding the nature of the genomic features considered: supervised ML models can indeed operate from known markers like the ones involved in direct association strategies, offering the possibility to discover more complex and multivariate marker combinations better predicting resistance phenotypes [3, 10, 17], or directly using the raw sequences represented as k-mers [8, 4, 21, 22, 23]. The latter approach offers several advantages: it does not require prior knowledge about the underlying resistance mechanisms, allows to capture various types of genomic determinants (including the acquisition of genes or point mutations), and does not require to align the genomes to a common reference which may be hard to define for some species, especially the less clonal ones [24, 25]. Second, regarding the type of ML algorithms. Boosting algorithms [8, 4, 21], penalized regression models [10, 17, 23], decision trees [26], random forest [10, 27], neural networks [17] or set cover machines [22, 26] have already been successfully deployed in this context. While each algorithm has its own merits and shortcomings, several studies reported comparable global performance for various algorithms, with specific variations by drug and microbial species [10, 17, 28]. Finally, different kinds of antibiotic susceptibility information can be considered: either discrete when the objective is to distinguish susceptible from resistant (or non-susceptible) ones [10, 17, 21, 22], or continuous, where one seeks to predict the minimum inhibitory concentration (MIC) of the antimicrobial agent itself [3, 8, 4].

A critical challenge for the adoption of such predictive ML models by clinicians and microbiologists resides in their level of interpretability and, ultimately, clinical action-driving ability. While the notion of interpretability is somehow ill-defined, a natural requirement for the end-user would be to achieve the prediction from a limited number of genomic features, that can be easily and unambiguously interpreted as actual genetic determinants [26, 25]. This challenge is particularly important using k-mer-based representations, for several reasons.

Firstly, k-mers covering conserved genomic regions are redundant, and while they can be easily detected and filtered [29], they define groups of equivalent k-mers which are not always straightforward to interpret as genomic determinants [21, 23, 22, 26]. Secondly, k-mers may not be specific of a given genomic region, hence may be hard to annotate. This is especially the case for short k-mers, e.g., when $k = 8$ or $k = 10$ [8, 4]. Last but not least, the k-mer-based representation of genomes intrinsically leads to very high-dimensional feature spaces, with strongly correlated variables. Using $k = 31$ for instance, and depending on the bacterial species considered, it is common to end up working with $10^5 - 10^6$ (non-redundant) k-mers, many of which are observed in *almost* the same sets of genomes, hence bringing almost the same information regarding the studied phenotype.

We propose to rely on the adaptive cluster lasso (ACL) [30], an extension of Bühlmann et al. [31] tailored to the high-dimension setting by means of a prior screening of variables. We implemented in a R package a simple and efficient ACL-inspired strategy able to cope with the very high-dimension and strong correlations of k-mer-based representation, leading to sparse and interpretable genomic signatures. This approach compared favorably to the standard lasso on a systematic validation study focusing on *K. pneumoniae*. It provided a comparable level of performance while offering better interpretability of the genomic determinants involved in the models. We could identify known and potentially novel resistance determinants from the corresponding k-mer signatures, which allowed to extract meaningful scientific insights.

Methods

Datasets

Training dataset We gathered the assembled genomes, provided as contigs, of 1665 strains to develop MIC prediction models for *K. pneumoniae* [8]. This set of genomes defines our training dataset. We focused on the 10 clinically most relevant antibiotics listed in Table 1 which belong to seven different antibiotic classes. The reference MICs were cast into resistant, susceptible and intermediate according to the Clinical and Laboratory Standards Institute (CLSI) breakpoints. The intermediate and resistant strains were finally merged into a common category, to define a binary classification problem aiming to distinguish susceptible (S) from non-susceptible (NS) strains. Table 1 provides the number of S/NS phenotypes available for each selected drug.

k-merization of the training dataset The k-merization was computed from the contigs of all training genomes, using the DBGWAS software [25], with a k-mer size of 31 and filtering patterns with a minor allele frequency (MAF) below 1%. As discussed in previous studies [22, 25], k=31 is a safe default choice, offering good predictive performance while preserving the specificity of the k-mers to particular genomic loci, which is key to annotate them properly. DBGWAS allows for the deduplication of the strictly equivalent k-mers by compacting overlapping non-branching paths of k-mers into unitigs, thanks to the use of a compacted De Bruijn Graph (cDBG) (Figure 1 A). DBGWAS stores the profiles of presence/absence of each unitig in the training genomes in a matrix $\mathbf{V}$ such as $\mathbf{V}_{i,j} = 1$ if the j-th unitig is present in the i-th input genome and $\mathbf{V}_{i,j} = 0$ otherwise (Figure 1, B1). Each vector $\mathbf{V}_{,j}$ is then transformed according to its allele frequency: if its allele frequency exceeds 0.5, meaning that it is observed in more than 50% of the panel genomes, it is inverted as $\mathbf{V}_{i,j} = |1 - \mathbf{V}_{i,j}|$ so that its MAF corresponds to its average value. This transformation renders identical two originally complementary vectors. Keeping only the unique patterns then leads to an optimal reduction of the number of features, without modifying the intrinsic statistical signal (Figure 1 B2). These unique, MAF-filtered, patterns define the final variant matrix $\mathbf{X}$, where $\mathbf{X}_{i,j} = 1$ if the jth pattern is found in the ith genome, and 0 otherwise. This process is described in details in Jaillard et al. [25]. The DBGWAS files describing the cDBG are kept for the further interpretation of the genomic signatures, allowing to visualize the unitigs of the selected patterns within their genomic environment.

In practice we carry out this k-merization process for each antibiotic separately, processing solely the strains that have been phenotypically tested. The output of this k-merization

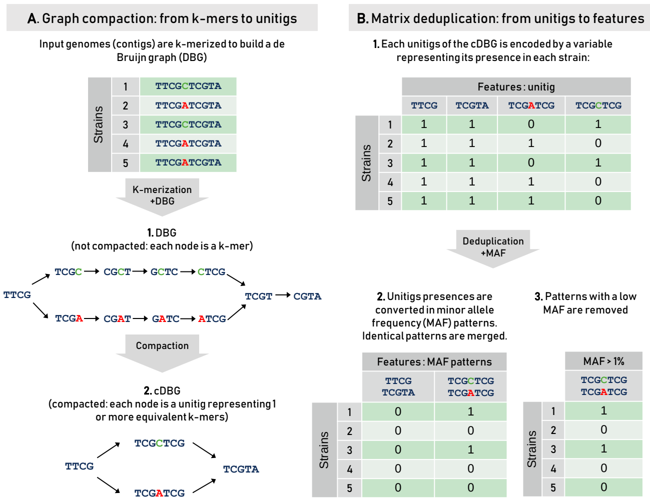

Figure 1. ***K*-merization of the training genomes.** Illustration of the DBGWAS process of k-merization and variant matrix construction. Refer to Jaillard et al. [25] for further details.

	training		test	
	NS	S	NS	S
amikacin	346	1319	191	160
aztreonam	1426	216	250	10
cefepime	961	608	235	53
cefoxitin	976	667	319	138
ceftazidime	1529	136	457	125
ciprofloxacin	1461	201	471	137
imipenem	504	1160	259	301
meropenem	524	1134	297	86
piper.tazo	1228	432	382	146
tetracycline	928	737	273	155

Table 1. Dataset constitution. This table provides the number of susceptible (S) and non-susceptible (NS) strains available in the training and test dataset for the various antibiotics considered. piper.tazo stands for piperacillin/tazobactam. Note that a limited number of susceptible strains is available in the test dataset for aztreonam, and to a lesser extent cefepime and meropenem.

step is a sparse variant matrix **X** with, for instance in the case of the cefoxitin antibiotic, $N = 1643$ rows for the N cefoxitin-phenotyped strains of the training panel and $p = 1,234,397$ columns representing the p distinct patterns of presence/absence retained by DBGWAS. As discussed in [32], this offers a drastic reduction of the amount of information to store, as the k-mers and unitigs-based matrices involve 85,623,165 and 3,396,675 columns respectively. The matrix **X** is binary as DBGWAS only encodes the presence or absence in the genomes. It is sparse as only around 13% of the values are not null.

Test dataset To validate the predictive performance of the models, we built an independent test dataset involving 634 strains, including 114 strains from our bioMérieux collection (NCBI Bioproject PRJNA449293 and PRJNA597427) and 520 strains from the PATRIC database (https://www.patricbrc.org/). Such strains were mostly from the USA, the UK, Serbia, Greece and other European countries and the MICs were obtained with either agar dilution, broth microdilution or VITEK 2 (bioMérieux, Marcy l'Étoile, France) (see Supplementary Section S1). Table 1 provides the number of S/NS phenotypes available in the test dataset.

Coping with highly correlated genomic features

Logistic regression is a widely used generalized linear model addressing binary classification problems. In our case, it consists of building a linear function defined for a strain represented by a vector $\mathbf{x} \in \{0,1\}^p$ as:

$$f(\mathbf{x}) = \beta_0 + \sum_{j=1}^{p} \beta_j \mathbf{x}_j, \quad (1)$$

where p corresponds to the number of distinct patterns identified by DBGWAS, and $\mathbf{x}$ encodes their presence/absence in the strain genome. To estimate the model coefficients and simultaneously select a limited number of patterns from a training panel of n strains, one can rely on the L_1 or lasso penalty and consider the following optimization problem:

$$\hat{\beta} = \arg\min_{\beta \in \mathbb{R}^{p+1}} \sum_{i=1}^{n} \mathcal{L}(y_i, f(\mathbf{X}_{i,.})) + \lambda \sum_{j=1}^{p} |\beta_j|,$$

where $y_i = 0$ if the ith strain, stored in the ith row of the training matrix $\mathbf{X}$, is susceptible and 1 otherwise. The function $\mathcal{L}$ is the logistic loss function, which quantifies the discrepancy between the true phenotypes y_i of the strains and the predictions $f(\mathbf{X}_{i,.})$ obtained by the model. The λ parameter achieves a trade-off between this empirical error and the lasso regularization term, and is usually optimized by cross-validation.

The feature selection ability of the lasso penalty is notoriously unstable in the presence of strong correlation between features. This is particularly the case using k-mer based representations, making it difficult to derive meaningful interpretations from the features selected by the model, and their associated coefficients. We propose a simple and efficient three-step strategy to identify sparse and interpretable genomic signatures.

Screening step In this step, we *screen* features. For this purpose, we first fit a standard lasso-penalized regression model on the original feature matrix $\mathbf{X}$ for several values of the regularization parameter λ, and extract the set of features that are selected at some point on this regularization path. Formally, letting $(\lambda_1, ..., \lambda_m)$ be the m values of the considered grid of λ, and $\mathbf{B}$ the $p \times m$ matrix containing the model coefficients obtained by Equation 1. We define a set $\mathbf{a}$ of *active features* as :

$$\mathbf{a} = \{i \in [1, \dots, p], \text{ such that } \max(|\mathbf{B}_{i,.}|) > 0\},$$

and let $p_a = |\mathbf{a}|$ be their number. Since the lasso cannot select more features than observations, we typically end up with p_a in the order of N (i.e., 10^3 in our case). We then extract the features which are strongly correlated to the active ones from the entire feature matrix. For this purpose, we compute a $p_a \times p$ matrix $\mathbf{G}$ containing the pairwise correlations between the p_a active features identified beforehand and the p original ones. Formally, $\mathbf{G}_{i,j} = \mathrm{cor}(\mathbf{X}_{.,\mathbf{a}_i}, \mathbf{X}_{.,j})$, where cor is the standard Pearson correlation between vectors of MAF patterns across the genomes, and is a classical criterion to quantify linkage disequilibrium (LD) between genomic features [33]. Since we rely on binary variables encoding the presence/absence of features in the genomes, $\mathbf{G}_{i,j}$ quantifies the extent to which features i and j co-occur in the genomes. As p_a is typically much smaller than p (in the orders of 10^3 *versus* 10^6 in our case), computing this matrix is much easier than computing the entire $p \times p$ correlation matrix. Finally, we extract the set $\mathbf{e}$ of features that are strongly correlated to at least one active feature as:

$$\mathbf{e} = \{i \in [1, \dots, p], \text{ such that } \max(\mathbf{G}_{.,i}) > s_1\},$$

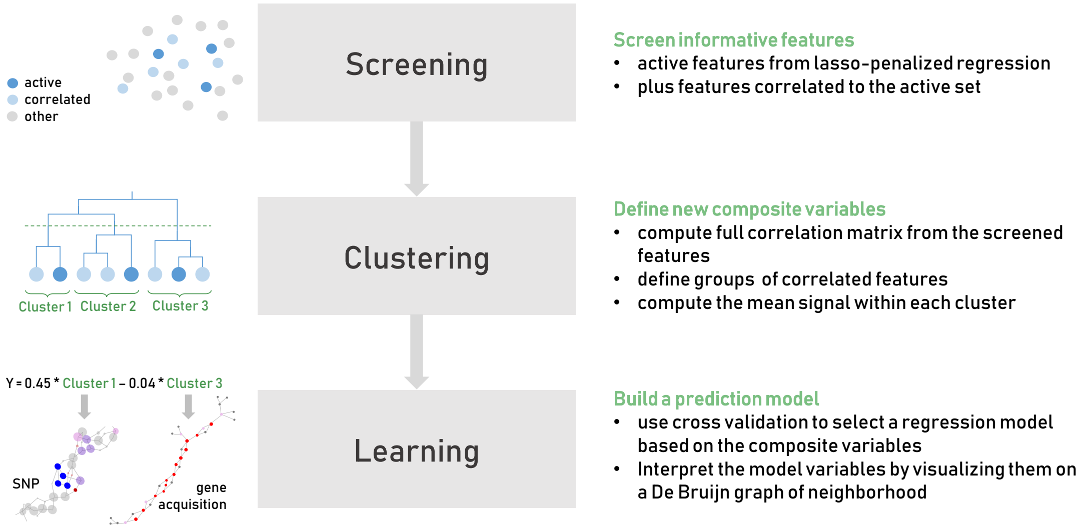

Figure 2. Three-step process. Illustration of the proposed three-step procedure.

where the hyperparameter s_1 controls the minimum level of correlation required, and is referred to as the *screening threshold*. This operation defines a set of $p_e = |\mathbf{e}|$ features, called the set of *extended features*. Obviously, we have $p_a \leq p_e \leq p$. In our context, we typically end up with a few thousand extended features, hence $p_a < p_e << p$.

Clustering step While the screening step identifies a limited number of features deemed sufficiently correlated to the features identified by a standard lasso, the second step aims to explicitly define groups, or *clusters*, of strongly correlated variables. We rely for this purpose on a bottom-up agglomerative clustering procedure, as suggested by Bühlmann et al. [31]. More precisely, we first define a $p_e \times p_e$ distance matrix $\mathbf{D}$ between extended features, defined as $\mathbf{D}_{i,j} = |1 - \texttt{cor}(\mathbf{X}_{\cdot,\mathbf{e}_i}, \mathbf{X}_{\cdot,\mathbf{e}_j})|$. This matrix is then used to carry out a hierarchical clustering, implemented in `R` by the `hclust` function, using a minimum linkage criterion. The resulting dendrogram is finally cut at a height of $1-s_2$, the second hyperparameter s_2, called the *clustering threshold*, controlling the level of within-cluster correlation.

Learning step Finally, we summarize each identified cluster as a new composite variable, defined as the average of the original variables defining the cluster, and carry out a standard lasso at the cluster level. Since in our case the original variables encode the presence/absence of a given DBGWAS pattern in the genomes, these composite variables correspond to the proportion of patterns involved in a cluster that are present/absent in the genomes. Figure 2 summarizes this three-step method.

Model selection

Our approach involves three hyperparameters that must be optimized for each antibiotic: the screening and clustering thresholds s_1 and s_2 used to build the clusters of correlated variables, and the regularization parameter λ involved in the final cluster-level lasso model. We relied on the `glmnet` software [34] to fit the lasso models involved in both the screening and learning steps. We used the default heuristic proposed by the software to define the grids of candidate values for the regularization parameters. The screening and clustering thresholds were both systematically set to 0.95 based on preliminary experiments (see Supplementary Section S2), and we relied on a 10-fold cross-validation procedure to optimize the regularization parameter involved in the final cluster-level lasso model, as we now describe.

We first split the training dataset into ten folds, stratified by sequence type and phenotype. While this cross-validation strategy may lead to an optimistic estimation of the predictive performance, it allows to measure the impact of regularization on generalization in a controlled setting where the model has a chance to generalize. For each of the ten folds, 9 tenth of the dataset were used to screen variables and identify clusters. The final cluster-level lasso model was then fit and applied to the held out strains, for each candidate value of the regularization parameter. Our model selection strategy aimed to simultaneously maximize its sensitivity and specificity, respectively defined as the fractions of correctly classified non-susceptible and susceptible strains. For this purpose, a Receiver Operating Characteristic (ROC) curve was built for each candidate regularization parameter after completion of the cross-validation procedure, and the point closest to the optimal one (defined by a true positive rate of 1 and a false positive rate of 0) was used to define the optimal sensitivity / specificity trade-off. Following Hicks et al. [28], we refer to the average of the (optimal) sensitivity and specificity as balanced accuracy (bACC). Finally, we selected the sparsest model that allowed to maximize the balanced accuracy up to one point, in order to reduce the risk of overfitting. In practice, this cross-validation procedure was repeated three times and the selection was based on average balanced accuracy values obtained across the three repetitions. Supplementary Figure S5 illustrates this model selection strategy.

Interpretation of the predictive signature

We use the DBGWAS software to interpret the genomic signatures, based on the cDBG built during the k-merization step. The unitigs defining the patterns involved in the final model are visualized within their neighborhood in the cDBG, which represents their genomic environment hence provides insight on the type of variant involved, typically a plasmid-based acquired gene *versus* a local mutation (single nucleotide polymorphism (SNP) or indel) in a chromosomal region.

Evaluation of the computational requirements

We evaluate the computational requirements of the standard lasso and cluster-lasso procedures by measuring the time and

	lasso				cluster-lasso			
	bACC	AUC	support	unitigs	bACC	AUC	support	unitigs
amikacin	92.7	95.4	16	22 (4)	92.3	95.7	11	93 (36)
aztreonam	76.7	81.9	31	45 (3)	76.9	82.3	28	425 (125)
cefepime	74	80.4	53	65 (3)	73.6	79.8	34	385 (111)
cefoxitin	82.4	88.7	134	155 (5)	82.2	88.6	171	1052 (221)
ceftazidime	91.6	95.8	51	69 (5)	90.7	95.3	43	863 (185)
ciprofloxacin	95.6	98.6	25	27 (2)	95.5	98.6	35	422 (139)
imipenem	93.1	93.6	10	10 (1)	92.7	93.4	7	241 (194)
meropenem	91.7	94	8	8 (1)	91.4	93.5	3	164 (159)
piper.tazo	81.6	89.6	127	144 (4)	81.5	89	120	1220 (226)
tetracycline	83	88.5	181	198 (3)	82.9	87.7	109	640 (104)

Table 2. Cross-validation results. This table summarizes the cross-validation results obtained by the lasso and cluster-lasso strategies for the 10 antibiotics, in terms of balanced accuracy (bACC), AUC, support size, overall number of unitigs involved and maximal number of unitigs associated to a single pattern or cluster (between brackets).

memory required to compute a regularization path involving 100 values of the regularization parameter. For the standard lasso, this simply amounts to calling the `glmnet` function of the `glmnet` R package, using the variant matrix provided by DBGWAS. For the cluster-lasso procedure, this amounts to:

i. making the same call to `glmnet` to identify the set of active variables,
ii. computing the $p_a \times p$ correlation matrix $\mathbf{G}$ in order to identify the set of extended features,
iii. building the clusters of correlated variables
iv. making a second call to `glmnet`, using the variant matrix defined at the cluster-level.

This procedure is repeated five times for each drug, using a single Xeon E5-2690-V3 CPU.

Results

Cross-validation results

Table 2 provides the results obtained in terms of cross-validation performance and support size of the models. The predictive performance is measured by the area under the ROC curve (AUC) and balanced accuracy. Additional performance indicators are provided in Supplementary Table S1. The support size of a model is defined as the number of features it involves, which respectively corresponds to individual or clusters of DBGWAS patterns, for the lasso and cluster-lasso strategies. We also report the overall number of unitigs involved, which is only slightly higher than the number of features for the lasso and corresponds to unitigs in total LD. In contrast, this overall number is markedly higher for the cluster-lasso strategy, because of the pattern clustering.

Both strategies show similar performance in terms of both balanced accuracy and AUC, confirming that taking into account, or not, the correlation between features has a limited impact in terms of predictive performance. We also note that the model support is often slightly smaller with cluster-lasso (for 8 drugs out of 10), suggesting that several features selected separately with the lasso ended up merged in a single cluster by the cluster-lasso. As expected, the overall number of unitigs involved in a cluster-lasso model is significantly larger. Interestingly, it is not evenly distributed across its features. In the meropenem model, for instance, 159 out of the 164 unitigs defining the model features are associated to a single feature, suggesting that it corresponds to the presence of a gene, as confirmed in the interpretation analysis depicted in the next section.

Finally, Figure 3 provides a graphical representation of the lasso and cluster-lasso signatures obtained for ceftazidime, which are of moderate complexity. The heatmap shows the correlation between the patterns involved in one signature and/or the other, and highlights the 8 major clusters identified by the cluster-lasso strategy (clusters including more than 10 patterns). While all the patterns defining a cluster have by construction a similar level of predictive power, the lasso model usually selected a single one of them. There is an exception for the 3rd cluster, shown in green in the zoomed area of Figure 3, where two patterns were selected as distinct features of the lasso model.

By explicitly reconstructing and providing these clusters of correlated features to the learning algorithm, the cluster-lasso strategy leads to a more meaningful characterization of the genetic determinants involved, as we describe below.

Model interpretation

We focus on two drugs to illustrate the improved interpretability offered by cluster-lasso signatures: meropenem, where the interpretation is straightforward, and cefoxitin, which is among the signatures of highest support. Additional results obtained for the remaining drugs are deferred to Supplementary Materials, Section S5.

As shown in Table 2, the lasso and cluster-lasso meropenem models involve 8 and 3 features, respectively. As shown in Figure 4(B), each lasso feature corresponds to a single unitig, while the cluster-lasso signature involves a large cluster of unitigs (159 out of the 164 involved). Figure 4(A) shows the magnitude of the model coefficients. It reveals that the cluster-lasso signature is essentially driven by a single prominent feature, while 4 to 5 features of the lasso signature have a non-negligible weight. The major feature of the cluster-lasso signature corresponds to the large cluster of correlated patterns, and the DBGWAS visualization (Figure 4(C)) shows that the corresponding unitigs are organized as a long linear path in the cDBG. This suggests that this cluster corresponds to an entire gene. The annotation provided by DBGWAS shows the gene to be the Class A beta-lactamase bla_{KPC}. The DBGWAS visualization obtained for the lasso signature indicates that 3 of the 8 features – features 1, 2 and 4 – are also co-located in a region of the cDBG annotated as bla_{KPC}. The fact that the lasso selected these specific unitigs within the bla_{KPC} gene suggests that the resistance determinants involved are SNPs or indels. While the gene-level annotation is the same as that obtained with the cluster-lasso, the interpretation of the signature in terms of genetic variants is therefore radically different. A closer look at the lasso signature reveals that the 3 bla_{KPC} features are actually strongly correlated: they are often observed together. Unsurprisingly, they belong to the largest cluster involved in the cluster-lasso signature, and interestingly, their cumulative weight is approximately equal to that of the cluster-lasso feature (3.4 instead

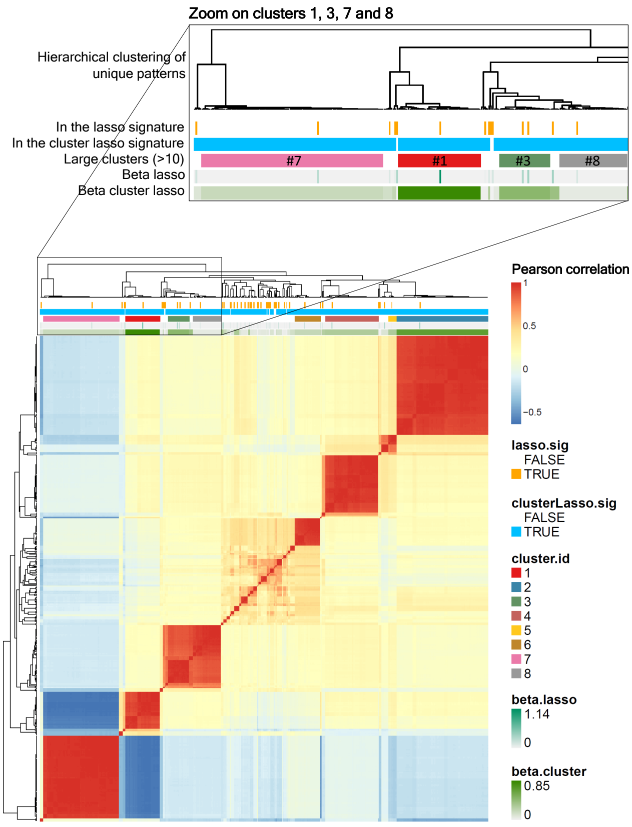

Figure 3. Correlation within features selected in the signatures. This heatmap shows the correlation matrix built from the features selected by the lasso and the cluster-lasso (identified by the orange and blue bars shown above the heatmap, respectively), for ceftazidime. The corresponding values of model coefficients are represented by green bars. The 8 major clusters (involving more than 10 patterns) of the cluster-lasso signatures are identified by a dedicated color ranging from red to grey. A zoom of the top left side of the figure allows a better reading of the colored bars for the major clusters 1, 3, 7 and 8.

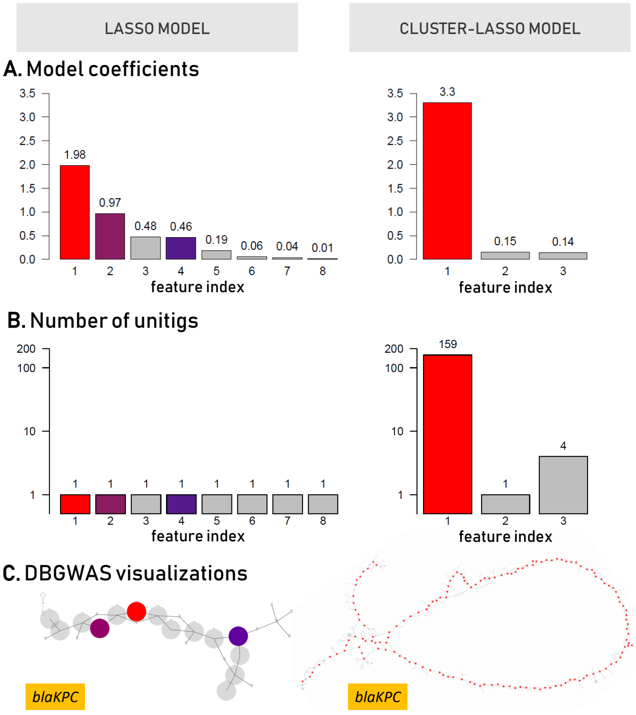

Figure 4. Interpretation of the meropenem signatures. This figure provides a detailed comparison of the lasso (left) and cluster-lasso (right) signatures. A) Absolute value of the coefficients of the models. B) Number of unitigs involved in the features of the models. C) Visualization of the first subgraph obtained by DBGWAS for each signature. Nodes of the graphs correspond to unitigs of the cDBG built by DBGWAS from the training panel of genomes, as illustrated in Figure 1 and detailed in [25]. Colors allow to identify which unitigs of the graphs in panel C are related to which features of the models in panels A and B.

of 3.3). By explicitly detecting that these features are correlated, and merging them into a single feature, together with additional correlated features not even involved in the lasso signature, the cluster-lasso leads to a more meaningful interpretation of the underlying prediction model, in two aspects. Firstly, it captures the true nature of the genomic determinant involved: the presence of the *bla*$_{KPC}$ gene, as opposed to mutations within the gene. Secondly, it assesses the overall contribution of the gene presence in the decision rule, while, in the lasso signature, this contribution is shared by several distinct yet correlated features.

Likewise, Figure 5 presents the DBGWAS analysis of the lasso and cluster-lasso signatures obtained for cefoxitin. We focused on the two first subgraphs provided by the software, which represent the two genomic neighborhoods of the most important patterns, or clusters of patterns, involved in the models. The subgraphs are indeed ordered according to the maximal absolute value of model coefficients among all patterns or clusters involved in the subgraph. While DBGWAS identifies the same resistance genes in both methods (the efflux pump *ompK36* and *bla*$_{KPC}$), the nature of the underlying resistance determinants cannot be deduced from the lasso signature. The *ompK36*-annotated subgraph obtained for the cluster-lasso signature (top-right panel of Figure 5) involves 2 clusters gathering 9 unitigs (clusters 1 and 3), and presents a topology attributable to a local polymorphism: a complex bubble, with a fork separating susceptible (blue) and resistant (red) strains, as described in [25]. The corresponding lasso subgraph, shown on the top-left panel, includes 4 patterns (patterns 1, 2, 32 and 56) each having its proper value of model coefficient, represented by 4 shades of colors ranging from blue to red. These distinct model coefficient values can lead to

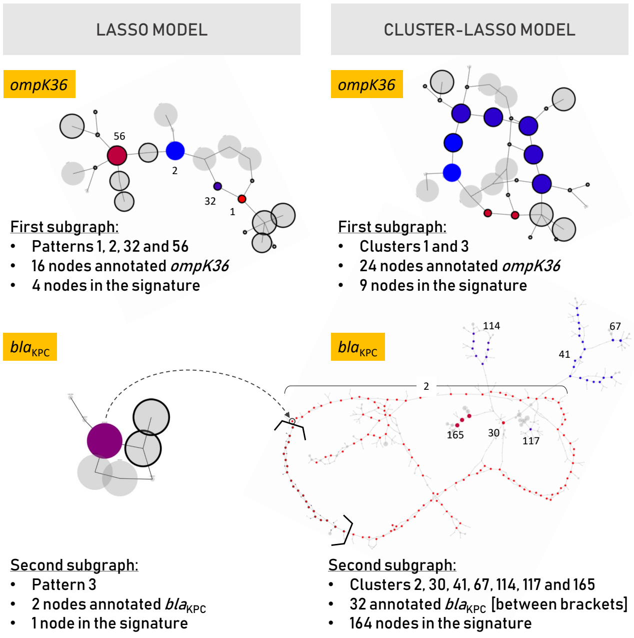

Figure 5. DBGWAS visualizations for the interpretation of the cefoxitin signatures. This figure presents the two first subgraphs obtained by DBGWAS for the lasso and cluster-lasso signatures. The DBGWAS subgraphs are ordered by decreasing maximal absolute value of model coefficient among all patterns/clusters involved in the subgraph. Likewise, pattern and cluster identifiers are ordered by decreasing absolute value of model coefficient, meaning for instance that pattern/cluster #1 has a greater weight in the model that pattern/cluster #2. The nodes (unitigs) belonging to patterns/clusters of the signatures are colored by the value of their model coefficients (from blue to red, indicating negative and positive values, respectively). The grey nodes/unitigs, not involved in the models, represent their genomic neighborhood. The nodes for which an annotation related to antibiotic resistance was found are surrounded by a black circle. Bold brackets are used on the bottom right subgraph to highlight these black-circled nodes. This particular subgraph gathers 7 clusters, whose identifiers are reported on the picture. Cluster 2 is the largest one, and includes the *bla*$_{\mathrm{KPC}}$-annotated nodes. The dashed arrow shows which node of the cluster-lasso *bla*$_{\mathrm{KPC}}$ subgraph corresponds to the one selected by the lasso.

wrong conclusions regarding the individual importance of the corresponding unitig sequences. Indeed, aligning these unitigs with annotated *ompK36* sequences reveals that features 2 and 56 both represent the wild type, while features 1 and 32 align to the insertion of two amino acids in the L3 loop, as described in Novais et al. [35] (Supplementary Figure S7). The second lasso subgraph (bottom-left panel of Figure 5) includes a single feature of the signature (shown in purple), surrounded by seven nodes (shown in grey), among which two are annotated as *bla*$_{\mathrm{KPC}}$. The node of the signature is however not annotated itself, hence the subgraph could be interpreted as a local polymorphism in the promoter region of the *bla*$_{\mathrm{KPC}}$ gene. The cluster-lasso subgraph shown on the bottom-right panel reveals however that this unitig was selected by the lasso among hundreds of highly correlated unitigs. They all belong to cluster 2, which includes the complete *bla*$_{\mathrm{KPC}}$ gene (shown between brackets) and plasmid sequences in strong LD.

By its ability to leverage correlations between patterns, the cluster-lasso approach allowed to identify that the second causal determinant involved in the cefoxitin model is the *bla*$_{\mathrm{KPC}}$ gene, which was brought by a plasmid. As it was the case for meropenem, it offers a far better interpretability than the lasso, which didn't even explicitly identified *bla*$_{\mathrm{KPC}}$ in its features, but only a specific sequence of its direct plasmidic environment. We emphasize however that this improved interpretability may have a price in terms of predictive performance. Indeed, correlations between genomic features may be overestimated if the training dataset is not diverse enough, which may lead to the reconstruction of oversized clusters. This may for instance be the case here of the second cluster-lasso cluster which identified *bla*$_{\mathrm{KPC}}$ within a specific plasmid, while *bla*$_{\mathrm{KPC}}$ is known to jump frequently between plasmids [36], many of which may not have been observed in the training set. Applying this model to a strain harboring *bla*$_{\mathrm{KPC}}$ in a different plasmidic environment may therefore fail to activate a sufficient number of patterns of this cluster, which may prevent recognizing the strain as resistant.

Performance on the test set

Table 3 shows the predictive performance obtained on the test set by the lasso and cluster-lasso signatures, in terms of sensitivity, specificity, balanced accuracy and AUC.

We first noted that the lasso and cluster-lasso strategies reached a similar level of balanced accuracy for most drugs, although they did not always achieve the same trade-off in terms of sensitivity and specificity. We noted however that the confidence intervals of the corresponding sensitivities and specificities largely overlapped for all drugs but ceftazidime (Figure 6 and Supplementary Figure S8), indicating that they were not significantly different between lasso and cluster-lasso, except for one drug.

We often observed a serious drop between the predictive performance estimated by cross-validation and that observed for the test set: more than 5 points of balanced accuracy for 6 drugs out of 10, and up to 10 points or more for amikacin, cefoxitin, imipenem and meropenem (13.4, 10.2, 10.9 and 9.9 points, respectively). This suggested that the training dataset taken from Nguyen et al. [8] could not account for the entire diversity displayed by *K. pneumoniae*. A simple analysis of the strain's resistomes and sequence types (ST) using the `kleborate` software[1] revealed that the prevalence of several STs and well known resistance genes was sometimes very different in the two panels. This latter point is illustrated in Figure 7 for amikacin and

1 https://github.com/katholt/Kleborate/

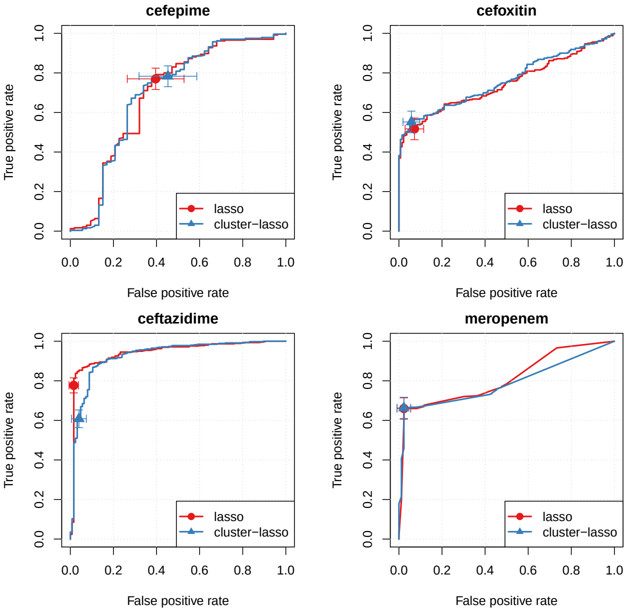

Figure 6. Test set results. This figure represents the ROC curves obtained for cefepime, cefoxitin, ceftazidime and meropenem by the lasso (red) and cluster-lasso (blue) signatures, as well as their associated sensitivities / specificities, with their 95% confidence intervals.

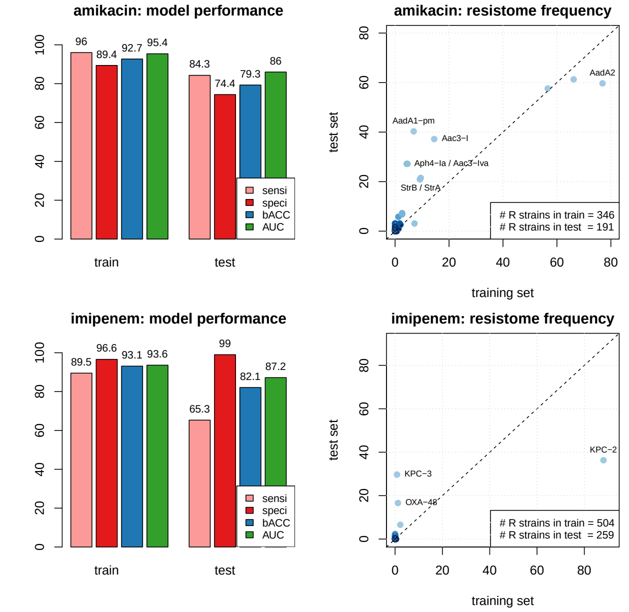

Figure 7. Resistome analysis. This figure compares the training and test panels of genomes in terms of predictive performance and resistome constitution for the drugs amikacin (top) and imipenem (bottom). Left: predictive performance in terms of sensibility, specificity, bACC and AUC estimated by cross-validation on the training set and measured on the test set, using the lasso signatures. Right: comparison of the resistome constitutions. Each `kleborate` resistance marker is represented by its prevalence in the resistant strains of the training (x-axis) and test (y-axis) panels.

imipenem, which suffered from the highest performance drop. Supplementary Figure S3 shows the difference in the ST prevalence, highlighting that the training set involves two main STs (ST307 and ST258), which have a much lesser prevalence in the test dataset. Redesigning the training and test datasets by shuffling the original ones in order to obtain an homogeneous split fixed this generalization issue (Supplementary Section S7). This illustrates that while machine learning models can indeed succeed in learning accurate prediction rules, they fail to generalize when the dataset they are trained on does not account for the overall diversity of the bacterial species.

Finally, Table 3 and Supplementary Figure S9 show an uneven level of prediction performance among the ten antibiotics considered. The best performances were obtained for ciprofloxacin and ceftazidime, with an AUC around 95% using either the original or the redesigned datasets (Supplementary Figure S9). The poorest performances were obtained for two beta-lactams: cefepime, a 4th-generation cephalosporin, and the monobactam aztreonam. This may be due to a reduced penetrance of their genetic determinants, as described in human genetics [37], because more complex resistance mechanisms are involved, including efflux pumps, gene regulation, or plasmid copy number [38, 39, 40].

Computational requirements

Figure 8 indicates that while the duration of the cluster-lasso was in average about three times longer than the lasso (571 vs 180 seconds), it took only about 10 minutes to obtain an entire regularization path defined at the cluster-level. Optimizing the regularization parameter using our cross-validation process therefore took approximately 5 hours on a single CPU. We noted that while the time required by the lasso was relatively homogeneous across drugs, it was more variable for the cluster-lasso. This variability was due to the fact that the lasso used in the first step identified a variable number of active features, which directly impacted the time required to screen the remaining ones. This is illustrated in Supplementary Figure S10.

In terms of memory, we noted that the cluster-lasso procedure led to an overhead of about 2 GB with respect to the lasso, which was related to the computation of the correlation matrix **G**. In practice, we limited this overhead by computing this matrix by slices, considering subsets of $p' = 10,000$ features and computing $p_a \times p'$ matrices instead of the entire $p_a \times p$ matrix at once. Altogether, this led to a computationally efficient procedure, allowing to identify cluster-level signatures in a few hours, for a limited memory footprint. We note that it could be straightforwardly parallelized, using several CPUs to compute the various slices of the correlation matrix **G**.

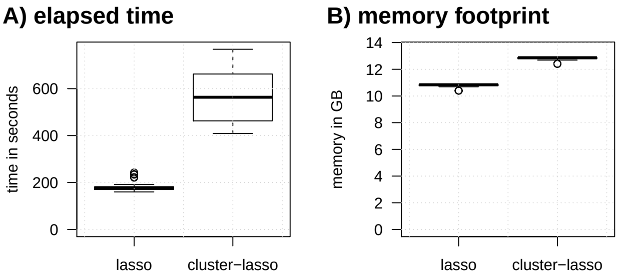

Figure 8. Time and memory requirements. The boxplots represent the variability of the time (panel A) and maximum memory (panel B) required to generate a lasso or cluster-lasso regularization path for the ten antibiotics.

Discussion

Representing bacterial genomes using k-mers leads to very high-dimensional representations with strong correlation structures. This may hinder a meaningful interpretation of predictive models built by sparse ML strategies like lasso-penalized regressions [41] or decision trees-based algorithms [42], which are known to be unstable in this case: when

	lasso				cluster-lasso			
	sensi	speci	bACC	AUC	sensi	speci	bACC	AUC
amikacin	84.3	74.4	79.3	86	77	80	78.5	86.4
aztreonam	69.6	80	74.8	83.3	67.2	80	73.6	82
cefepime	77	60.4	68.7	69.1	78.3	54.7	66.5	69.6
cefoxitin	51.7	92.8	72.2	74.6	55.2	94.2	74.7	76
ceftazidime	77.7	98.4	88.1	94.3	60.8	96	78.4	92.8
ciprofloxacin	91.3	91.2	91.2	96.6	92.1	89.8	90.9	96.7
imipenem	65.3	99	82.2	87.2	65.6	98.3	81.9	85.4
meropenem	66	97.7	81.8	81.1	66.3	97.7	82	78.8
piper.tazo	63.1	82.9	73	82.7	58.9	87.7	73.3	81.6
tetracycline	64.8	93.5	79.2	82.4	64.5	94.8	79.7	82.8

Table 3. Test set results. This table summarizes the results obtained on the test dataset by the lasso and cluster-lasso models for the 10 antibiotics, in terms of sensitivity, specificity, balanced accuracy (bACC) and AUC.

some features are strongly correlated, they tend to pick one, or few ones, out of them arbitrarily [43]. This instability may not be an issue in terms of predictive performance: as long as one feature among a group of correlated ones appears in the model, the prediction may be unchanged. It may however have a severe impact in terms of interpretability, as the features selected by the model may provide an incomplete or erroneous characterization of the causal resistance determinant.

We propose a simple and computationally efficient strategy to cope with the strong correlation structures inherent to k-mer-based representations, and build sparse and meaningful genomic signatures. While performing a systematic study on thousands of strains of *K. pneumoniae*, our approach compared favorably to the state of the art, providing indeed a comparable level of performance, while offering a greater interpretability of the genomic features involved in the models. On this challenging genetically flexible bacterial species with significant accessory genome components, this new approach allowed to extract meaningful scientific insights from the identified signatures, as further detailed in Section S5 of the Supplementary Materials.

Central to our approach is a three-step strategy, where a sparse ML algorithm is first used to screen features in a generic manner, which are then extended to clusters of strongly correlated features, ultimately considered as candidate features to be included in the final antibiotic resistance prediction model. Both the screening and final learning stages were based in this work on the lasso-penalized logistic regression model, which is appealing in this context for several reasons. First and foremost, it has been shown to be competitive to alternative ML algorithms in several papers (e.g., [26, 10, 17, 44]). The underlying model is moreover easy to interpret, as it combines several genomic determinants in a global probabilistic model with weights modulating their respective effects, hence reflecting the fact that they can be associated with different levels of resistance. Last but not least, the `R` package `glmnet` offers a very efficient implementation, scaling gracefully to large datasets like the one involved in this study, as shown in Figure 8. The principle of our method is nevertheless generic and could readily be transposed to other sparse ML algorithms, like xgboost [8, 4] or set cover machines [26]. Likewise, it could straightforwardly be extended to handle MICs or other phenotypic traits, as well as other types of genomic features (e.g., relying on SNPs instead of k-mers).

Several alternative strategies could be considered to handle correlations between k-mers. Most related to our approach are the elastic-net and the group-lasso strategies, which also rely on logistic regression – and more generally on generalized linear models – but with alternative regularization penalties. The elastic-net penalty combines the lasso and the ridge penalties, which leads to sparse models with a grouping mechanism: correlated features tend to be selected together [45]. This approach was recently shown to be efficient in the context of bacterial genome-wide association studies (GWAS), providing increased statistical power for the identification of genotype-phenotype associations and accurate prediction rules [46]. As we demonstrate in Supplementary Section S9, however, it remains limited in its ability to provide interpretable predictive signatures, for several reasons. First, while it has the effect of stabilizing the lasso solution and of simultaneously activating groups of correlated features, these groups are not defined explicitly, which intrinsically makes the interpretation of the model difficult. Moreover, while the parameter controlling the trade-off between the lasso and ridge penalties had a direct impact on the number of selected features, it had little impact on the predictive performance of the model, thereby making it difficult to optimize objectively. Finally, we empirically observed that it led to a partial and heterogeneous reconstruction of the genomic features obtained by the cluster-lasso: a significant fraction of the cluster members were not selected by the elastic-net, and the individual weights associated to the selected ones greatly varied, although their level of predictive power was comparable.

The group-lasso penalty leverages pre-defined groups of features, ensuring that all features of a given group are either active or inactive simultaneously [47]. This strategy was for instance considered in human GWAS, using groups of SNPs defined spatially to account for their LD [48]. Transposing this idea to bacterial genomes is challenging since no such prior information is available to define groups, as LD can be genome-wide [29]. A solution could be to identify clusters of correlated k-mers using agglomerating strategies [31], but is hard to carry out in practice from the high-dimensional datasets involving 10^5 – 10^6 features encountered in our application. Our approach can therefore be seen as a simple and efficient strategy to approximate such a group-lasso process in very high-dimensional settings. Instead of collapsing groups of correlated features into composite variables, a natural extension of our method would however be to rely on a group-lasso penalized regression defined at the cluster level. Each feature would then be granted its own weight, which could allow to better reflect their individual predictive power. We empirically observed that the weights variability within a cluster was very small, as shown in Supplementary Figure S14, which therefore indicated that keeping the features separated or averaging them is essentially equivalent. In practice, we find it easier to explicitly collapse each cluster to a single composite variable to interpret the model parameters.

On the practical side, our method involves two hyper-parameters, besides the regularization parameter, to identify active variables and to build the final model. Although these so-called screening and clustering thresholds did not have a strong influence in this study (Supplementary Section S2), they may be cumbersome to optimize in practice for other applications.

A natural extension to our method would be to consider resampling strategies in the clustering step, in order to identify stable clusters, whose constitution would be robust to the precise definition of the clustering threshold [49]. Alternatively, one could rely on tree-guided lasso penalization to leverage the entire dendrogram during the final learning step, which would then simultaneously identify clusters and learn the prediction model [50]. Regarding AMR prediction, our study performed on *K. pneumoniae* confirms several observations made recently, namely that k-mer-based approaches can learn sparse prediction rules without any prior information and that the level of predictive performance can vary by antibiotic [28, 26]. Importantly, our study involved a novel panel of 634 *K. pneumoniae* strains for the validation of the prediction models and suggested that the problem is more challenging than reported in Nguyen et al. [8]. The results they reported were indeed probably optimistic because the genome panel they considered did not account for the overall genomic diversity of *K. pneumoniae* as a species, as it involved two over-represented STs (ST307 and ST258) representing 60% of the isolates (Supplementary Figure S3). The 634 additional strains with genomes and phenotypes considered in this study will help learning more accurate and generalizable predictions models, as suggested by the preliminary experiments described in Supplementary Section S7. Another limitation of the current study lies in the fact that the phenotypic AST methods used to define the reference MICs differed between the training set (which involved the Phoenix technology (Becton Dickinson, Franklin Lakes, USA) only) and the test set (which was based on agar dilution, broth micro-dilution or VITEK 2). Indeed, AST is notoriously subject to a high level of technical variability [51], which intrinsically brings noise to the reference labels used to train and validate supervised ML models. A natural question therefore arises whether a ML model learned from data provided by a given AST method will generalize to data provided by a different AST method. A dedicated study described in Supplementary Section S11 suggests that this issue was not critical on this dataset, which therefore suggests that the lack of generalization observed on the test set is mainly driven by its genomic heterogeneity with respect to the training set.

Finally, the ML methods developed in this study are available in a generic R package that can be easily transposed to other bacterial species, as shown in Supplementary Section S12, and even other applications, not necessarily involving k-mers nor AMR phenotypes. On the challenging dataset considering in this study, involving more than a thousand strains for more than a million genomic features, the computational requirements remained limited and the signatures could be identified in a few hours on a standard workstation. Coupled with the enriched level of intepretability they offer, we believe our approach will help defining prediction models amenable to routine diagnostics.

Availability of source code and requirements

A generic R package implementing the proposed ML strategy is available at: https://gitlab.com/biomerieux-data-science/clustlasso. It is referenced as *RRID:SCR_018820* in the SciCrunch.org database and as *clustlasso* in the bio.tools database. A step-by-step procedure describing its integration with DBGWAS is available at: https://gitlab.com/biomerieux-data-science/clustlasso-dbgwas-integration.

Availability of supporting data and materials

All the genomes and associated phenotypes involved in this study are publicly available (data provided in additional file mentioned below).

Additional files

- pdf file with supplementary figures and analyses.
- csv file containing genome accessions and phenotypes.
- xls file describing the annotations of the signatures.

Declarations

Abbreviations

ACL: adaptive cluster lasso; AMR: antimicrobial resistance; AST: antibiotic susceptibility testing; AUC: area under the (ROC) curve; bACC: balanced accuracy; cDBG: compacted De-Bruijn graph; CLSI: clinical and laboratory standards institute; GWAS: genome-wide association study; LD: linkage disequilibrium; MAF: minor allele frequency; MIC: minimum inhibitory concentration; ML: machine learning; NGS: next-generation sequencing; NS: non-susceptible; ROC: receiver operating characteristic; S: susceptible; SNP: single nucleotide polymorphism.

Competing Interests

All authors are employees of bioMérieux, a company creating and developing infectious disease diagnostics. No further potential conflicts of interest relevant to this article are reported.

Funding

The work performed by MP was funded by the European Union's Horizon 2020 research and innovation program under the Marie Skłodowska-Curie Grant Agreement No 675412 (New Diagnostics for Infectious Diseases [ND4ID]).

Author's Contributions

PM and MD conceived and designed the experiments, performed the experiments, analyzed the data, authored or reviewed drafts of the paper, approved the final draft. MP collected and prepared the test dataset, carried out the resistome analysis, authored or reviewed drafts of the paper, approved the final draft. AvB authored or reviewed drafts of the paper, approved the final draft.

Acknowledgements

We thank Professor Herman Goossens for supporting MP.

References

1. Gordon NC, Price JR, Cole K, Everitt R, Morgan M, Finney F, et al. Prediction of *Staphylococcus aureus* Antimicrobial Resistance by Whole-Genome Sequencing. Journal of Clinical Microbiology 2014;52(4):1182–1191.
2. Walker TM, Kohl TA, Omar SV, Hedge J, Elias CDO, Bradley P, et al. Whole-genome sequencing for prediction of *Mycobacterium tuberculosis* drug susceptibility and resistance:

a retrospective cohort study. the Lancet Infections Diseases 2015;15:1193–1202.

3. Eyre DW, De Silva D, Cole K, Peters J, Cole MJ, Grad YH, et al. WGS to predict antibiotic MICs for *Neisseria gonorrhoeae*. The Journal of Antimicrobial Chemotherapy 2017;72(7):1937–1947.
4. Nguyen M, Long SW, McDermott PF, Olsen RJ, Olson R, Stevens RL, et al. Using Machine Learning To Predict Antimicrobial MICs and Associated Genomic Features for Nontyphoidal *Salmonella*. Journal of Clinical Microbiology 2019;57(2). https://jcm.asm.org/content/57/2/e01260-18.
5. Tyson GH, McDermott PF, Li C, Chen Y, Tadesse DA, Mukherjee S, et al. WGS accurately predicts antimicrobial resistance in *Escherichia coli*. Journal of Antimicrobial Chemotherapy 2015;70(10).
6. Moradigaravand D, Palm M, Farewell A, Mustonen V, Warringer J, Parts L. Prediction of antibiotic resistance in *Escherichia coli* from large-scale pan-genome data. PLOS Computational Biology 2018;14(12):1–17. https://doi.org/10.1371/journal.pcbi.1006258.
7. Deng X, Memari N, Teatero S, Athey T, Isabel M, Mazzulli T, et al. Whole-genome Sequencing for Surveillance of Invasive Pneumococcal Diseases in Ontario, Canada: Rapid Prediction of Genotype, Antibiotic Resistance and Characterization of Emerging Serotype 22F. Frontiers in Microbiology 2016;7:2099. https://www.frontiersin.org/article/10.3389/fmicb.2016.02099.
8. Nguyen M, Brettin T, Long SW, Musser JM, Olsen RJ, Olson R, et al. Developing an in silico minimum inhibitory concentration panel test for *Klebsiella pneumoniae*. Scientific reports 2018;8(1):421. http://www.ncbi.nlm.nih.gov/pubmed/29323230http://www.pubmedcentral.nih.gov/articlerender.fcgi?artid=PMC5765115.
9. Su M, Satola SW, Read TD. Genome-Based Prediction of Bacterial Antibiotic Resistance. Journal of Clinical Microbiology 2019;57(3). https://jcm.asm.org/content/57/3/e01405-18.
10. Yang Y, Niehaus KE, Walker TM, Iqbal Z, Walker AS, Wilson DJ, et al. Machine Learning for Classifying Tuberculosis Drug-Resistance from DNA Sequencing Data. Bioinformatics 2017;p. btx801. +http://dx.doi.org/10.1093/bioinformatics/btx801.
11. Coll F, McNerney R, Preston MD, Guerra-Assunção JA, Warry A, Hill-Cawthorne G, et al. Rapid determination of anti-tuberculosis drug resistance from whole-genome sequences. Genome Medicine 2015;7(1):51.
12. Bradley P, Gordon NC, Walker TM, Dunn L, Heys S, Huang B, et al. Rapid antibiotic-resistance predictions from genome sequence data for *Staphylococcus aureus* and *Mycobacterium tuberculosis*. Nature Communications 2015;6:10063.
13. Tanmoy AM, Westeel E, De Bruyne K, Goris J, Rajoharison A, Sajib MS, et al. *Salmonella enterica* Serovar Typhi in Bangladesh: exploration of genomic diversity and antimicrobial resistance. mBio 2018;9(6):e02112–18.
14. Miotto P, Tessema B, Tagliani E, Chindelevitch L, Starks AM, Emerson C, et al. A standardised method for interpreting the association between mutations and phenotypic drug resistance in *Mycobacterium tuberculosis*. European Respiratory Journal 2017;50(6). http://erj.ersjournals.com/content/50/6/1701354.
15. Mahé P, El Azami M, Barlas P, Tournoud M. A large scale evaluation of TBProfiler and Mykrobe for antibiotic resistance prediction in *Mycobacterium tuberculosis*. PeerJ 2019 May;7:e6857. https://doi.org/10.7717/peerj.6857.
16. Gygli SM, Borrell S, Trauner A, Gagneux S. Antimicrobial resistance in *Mycobacterium tuberculosis*: mechanistic and evolutionary perspectives. FEMS Microbiology Reviews 2017 03;41(3):354–373. https://doi.org/10.1093/femsre/fux011.
17. Chen ML, Doddi A, Royer J, Freschi L, Schito M, Ezewudo M, et al. Beyond multidrug resistance: Leveraging rare variants with machine and statistical learning models in *Mycobacterium tuberculosis* resistance prediction. EBioMedicine 2019;https://doi.org/10.1016/j.ebiom.2019.04.016.
18. Palomino JC, Martin A. Drug resistance mechanisms in *Mycobacterium tuberculosis*. Antibiotics 2014;3:317–340.
19. Palmer AC, Kishony R. Understanding, predicting and manipulating the genotypic evolution of antibiotic resistance. Nature Review Genetics 2013;14:243–248.
20. van Belkum A, Burnham CAD, Rossen JWA, Mallard F, Rochas O, Dunne Jr WM. Innovative and rapid antimicrobial susceptibility testing systems. Nature Reviews Microbiology 2020;in press.
21. Davis JJ, Boisvert S, Brettin T, Kenyon RW, Mao C, Olson R, et al. Antimicrobial Resistance Prediction in PATRIC and RAST. Scientific Reports 2016;6:27930.
22. Drouin A, Giguère S, Déraspe M, Marchand M, Tyers M, Loo VG, et al. Predictive computational phenotyping and biomarker discovery using reference-free genome comparisons. BMC genomics 2016;17(1):1–15.
23. Mahé P, Tournoud M. Predicting bacterial resistance from whole-genome sequences using k-mers and stability selection. BMC Bioinformatics 2018 Oct;19(1):383. https://doi.org/10.1186/s12859-018-2403-z.
24. Lees JA, Vehkala M, Välimäki N, Harris SR, Chewapreecha C, Croucher NJ, et al. Sequence element enrichment analysis to determine the genetic basis of bacterial phenotypes. Nature Communications 2016;7(12797).
25. Jaillard M, Lima L, Tournoud M, Mahé P, van Belkum A, Lacroix V, et al. A fast and agnostic method for bacterial genome-wide association studies: Bridging the gap between k-mers and genetic events. PLOS Genetics 2018 11;14(11):1–28. https://doi.org/10.1371/journal.pgen.1007758.
26. Drouin A, Letarte G, Raymond F, Marchand M, Corbeil J, Laviolette F. Interpretable genotype-to-phenotype classifiers with performance guarantees. Scientific Reports 2019 dec;9(1).
27. Farhat MR, Sultana R, Iartchouk O, Bozeman S, Galagan J, Sisk P, et al. Genetic determinants of drug resistance in *Mycobacterium tuberculosis* and their diagnostic value. Am J Respir Crit Care Med 2016 2016 Sep 1;194(5):621–30.
28. Hicks AL, Wheeler N, Sanchez-Buso L, Rakeman JL, Harris SR, Grad YH. Evaluation of parameters affecting performance and reliability of machine learning-based antibiotic susceptibility testing from whole genome sequencing data. PLOS Computational Biology 2019;15(9):e1007349. https://doi.org/10.1371/journal.pcbi.1007349.
29. Earle SG, Wu CH, Charlesworth J, Stoesser N, Gordon NC, Walker TM, et al. Identifying lineage effects when controlling for population structure improves power in bacterial association studies. Nature Microbiology 2016;1(16041).
30. Gauraha N, Parui SK. Efficient clustering of correlated variables and variable selection in high-dimensional linear models. arXiv preprint arXiv:160303724 2016;.
31. Bühlmann P, Rütimann P, van de Geer S, Zhang CH. Correlated variables in regression: Clustering and sparse estimation. Journal of Statistical Planning and Inference 2013;143:1835–1858.
32. Jaillard M, Tournoud M, Lima L, Lacroix V, Veyrieras JB, Jacob L. Representing genetic determinants in bacterial GWAS with compacted De Bruijn graphs. bioRxiv 2017;p. 113563.

33. Slatkin M. Linkage disequilibrium—understanding the evolutionary past and mapping the medical future. Nature reviews genetics 2008;9(6):477.
34. Friedman J, Hastie T, Tibshirani R. Regularization Paths for Generalized Linear Models via Coordinate Descent. Journal of Statistical Software 2010;33(1):1–22. http://www.jstatsoft.org/v33/i01/.
35. Novais A, Rodrigues C, Branquinho R, Antunes P, Grosso F, Boaventura L, et al. Spread of an OmpK36-modified ST15 *Klebsiella pneumoniae* variant during an outbreak involving multiple carbapenem-resistant *Enterobacteriaceae* species and clones. European journal of clinical microbiology & infectious diseases 2012;31(11):3057–3063.
36. David S, Cohen V, Reuter S, Sheppard AE, Giani T, Parkhill J, et al. Genomic analysis of carbapenemase-encoding plasmids from Klebsiella pneumoniae across Europe highlights three major patterns of dissemination. bioRxiv 2019;https://www.biorxiv.org/content/early/2019/12/19/2019.12.19.873935.
37. Cooper DN, Krawczak M, Polychronakos C, Tyler-Smith C, Kehrer-Sawatzki H. Where genotype is not predictive of phenotype: towards an understanding of the molecular basis of reduced penetrance in human inherited disease. Human genetics 2013;132(10):1077–1130.
38. Hocquet D, Nordmann P, El Garch F, Cabanne L, Plésiat P. Involvement of the MexXY-OprM efflux system in emergence of cefepime resistance in clinical strains of *Pseudomonas aeruginosa*. Antimicrobial agents and chemotherapy 2006;50(4):1347–1351.
39. Pages JM, Lavigne JP, Leflon-Guibout V, Marcon E, Bert F, Noussair L, et al. Efflux pump, the masked side of ß-lactam resistance in *Klebsiella pneumoniae* clinical isolates. PLoS One 2009;4(3):e4817.
40. Kitchel B, Rasheed JK, Endimiani A, Hujer AM, Anderson KF, Bonomo RA, et al. Genetic factors associated with elevated carbapenem resistance in KPC-producing *Klebsiella pneumoniae*. Antimicrobial agents and chemotherapy 2010;54(10):4201–4207.
41. Tibshirani R. Regression shrinkage and selection via the lasso. Journal of the Royal Statistical Society: Series B (Methodological) 1996;58(1):267–288.
42. Chen T, Guestrin C. Xgboost: A scalable tree boosting system. In: Proceedings of the 22nd acm sigkdd international conference on knowledge discovery and data mining ACM; 2016. p. 785–794.
43. Hastie T, Tibshirani R, Wainwright M. Statistical Learning with Sparsity: The Lasso and Generalizations. Chapman & Hall/CRC; 2015.
44. Macesic N, Bear Don't Walk OJ, Pe'er I, Tatonetti NP, Peleg AY, Uhlemann AC. Predicting Phenotypic Polymyxin Resistance in Klebsiella pneumoniae through Machine Learning Analysis of Genomic Data. mSystems 2020;5(3). https://msystems.asm.org/content/5/3/e00656-19.
45. Zou H, Hastie T. Regularization and variable selection via the elastic net. Journal of the royal statistical society: series B (statistical methodology) 2005;67(2):301–320.
46. Lees JA, Mai TT, Galardini M, Wheeler NE, Horsfield ST, Parkhill J, et al. Improved Prediction of Bacterial Genotype-Phenotype Associations Using Interpretable Pangenome-Spanning Regressions. mBio 2020;11(4). https://mbio.asm.org/content/11/4/e01344-20.
47. Yuan M, Lin Y. Model selection and estimation in regression with grouped variables. Journal of the Royal Statistical Society: Series B (Statistical Methodology) 2006;68(1):49–67.
48. Dehman A, Ambroise C, Neuvial P. Performance of a blockwise approach in variable selection using linkage disequilibrium information. BMC bioinformatics 2015;16(1):148.
49. Kimes PK, Liu Y, Hayes DN, Marron JS. Statistical significance for hierarchical clustering. Biometrics 2014;73(3):811–821.
50. Kim S, Xing EP. Tree-guided group lasso for multi-task regression with structured sparsity. In: International Conference on Machine Learning; 2010. p. 543–550.
51. Brennan-Krohn T, Smith KP, Kirby JE. The Poisoned Well: Enhancing the Predictive Value of Antimicrobial Susceptibility Testing in the Era of Multidrug Resistance. Journal of Clinical Microbiology 2017;55(8):2304–2308. https://jcm.asm.org/content/55/8/2304.

Click here to access/download;Figure;fig1.pdf

Figure 1

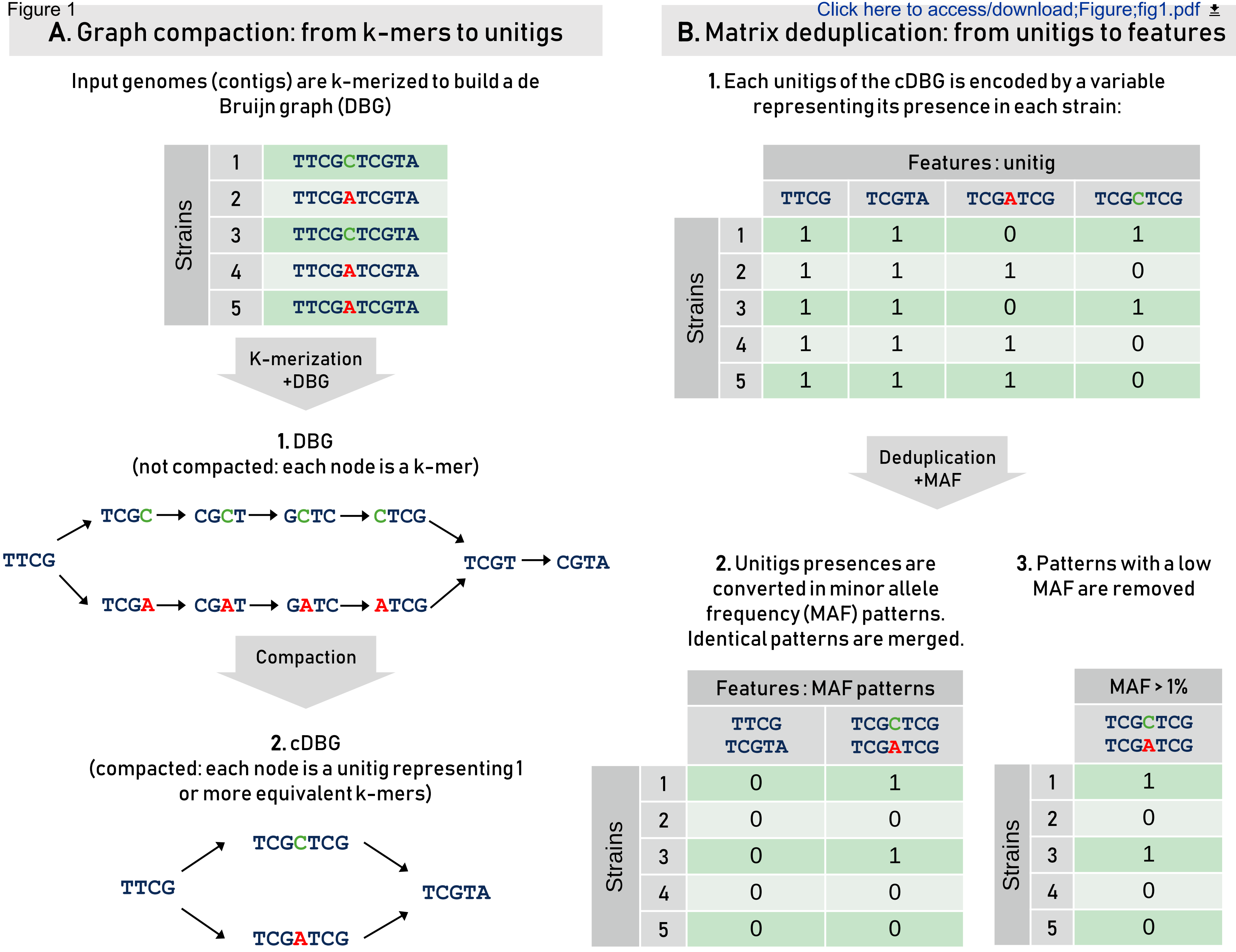

Figure 2

Click here to access/download;Figure;fig2.pdf

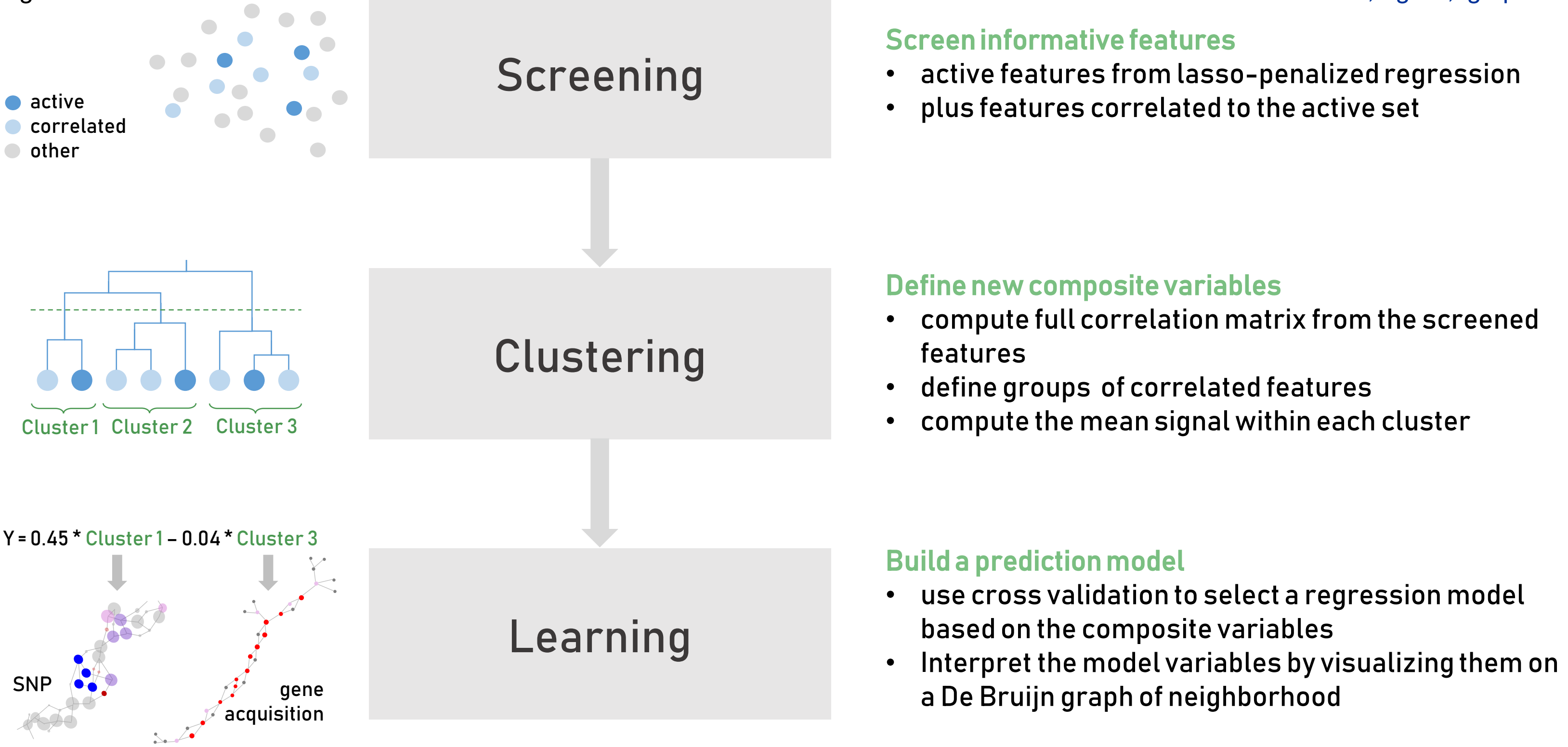

Figure 3

Click here to access/download;Figure;fig3.pdf

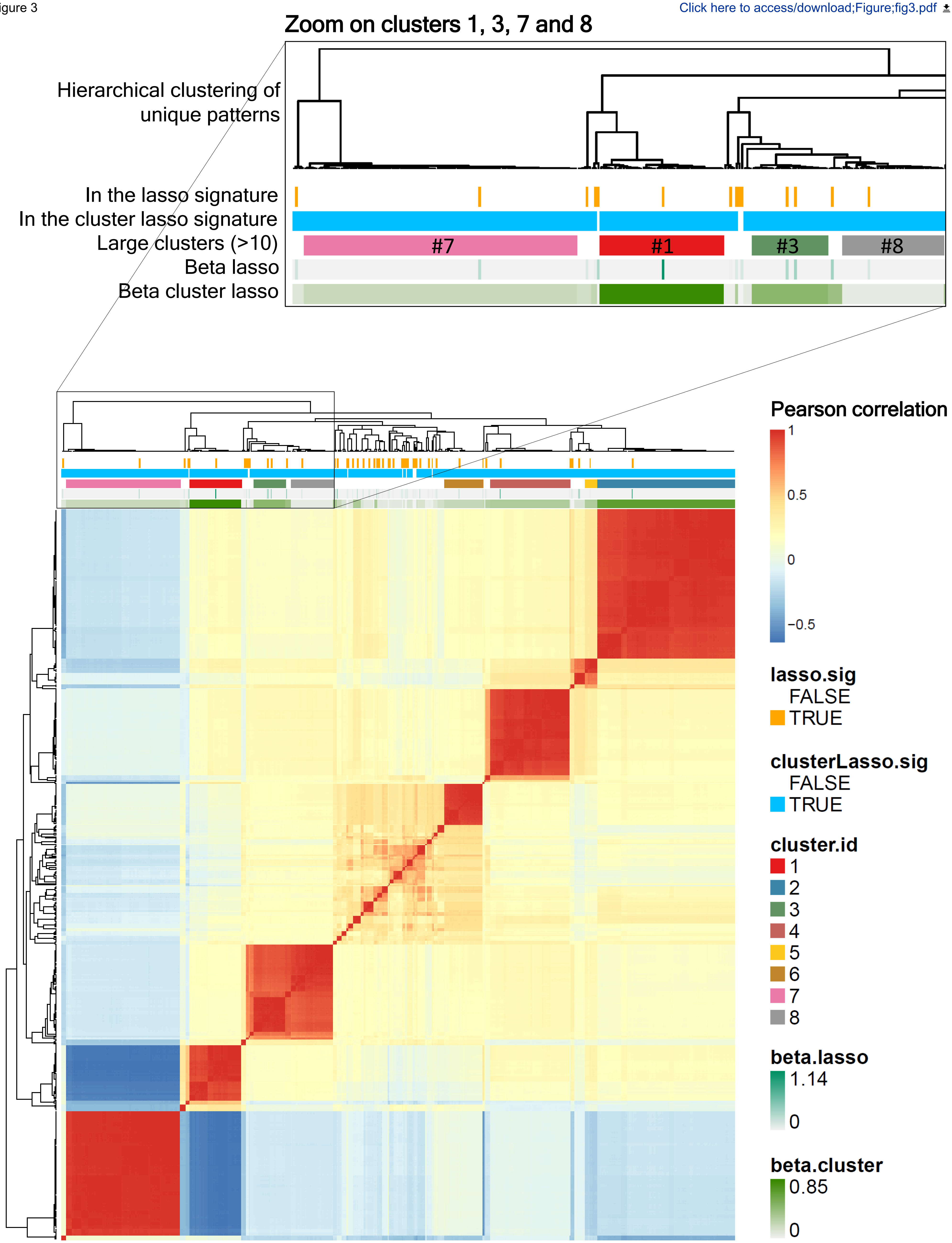

Figure 4

Click here to access/download;Figure;fig4.pdf

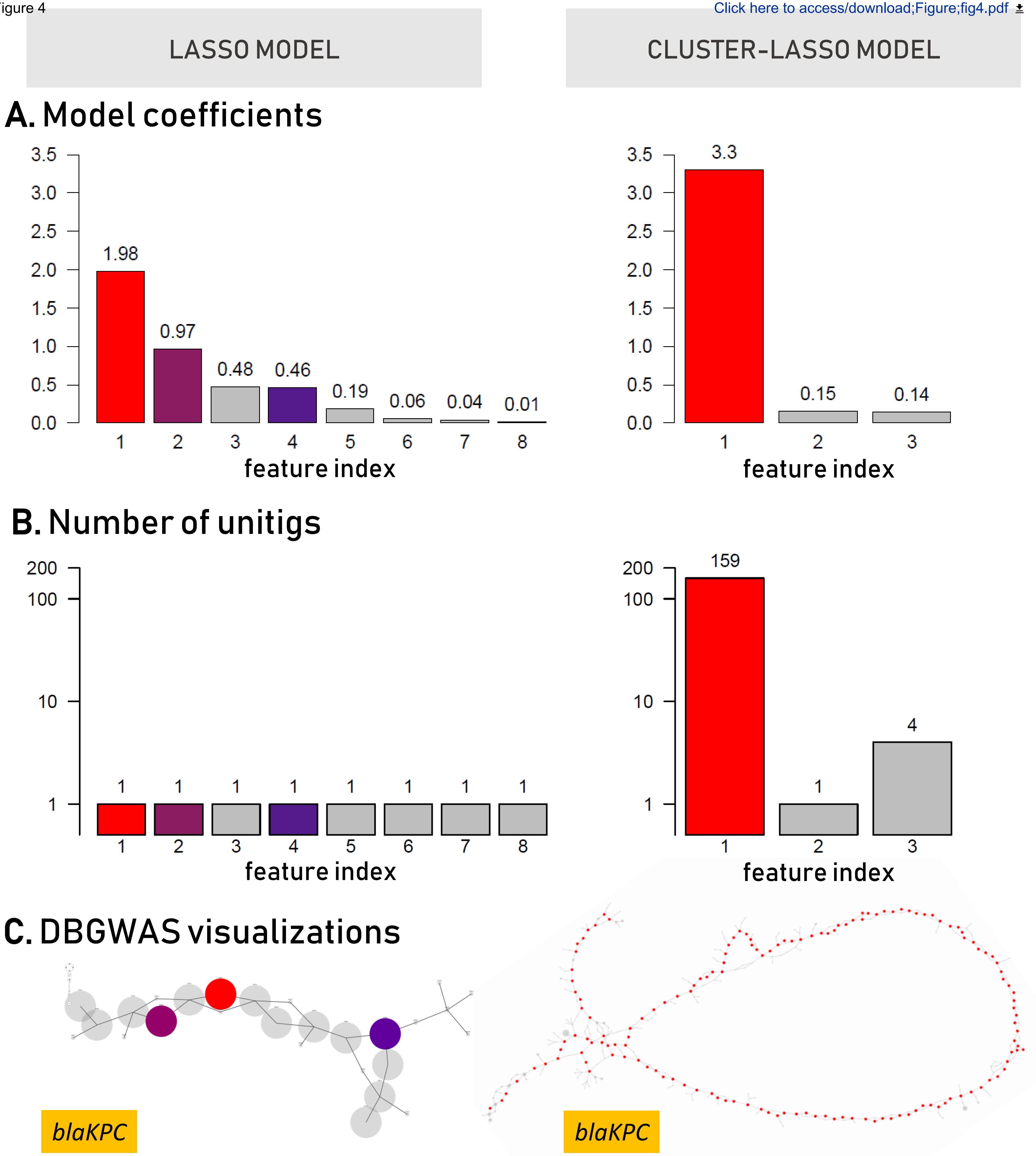

Figure 5

Click here to access/download;Figure;fig5.pdf

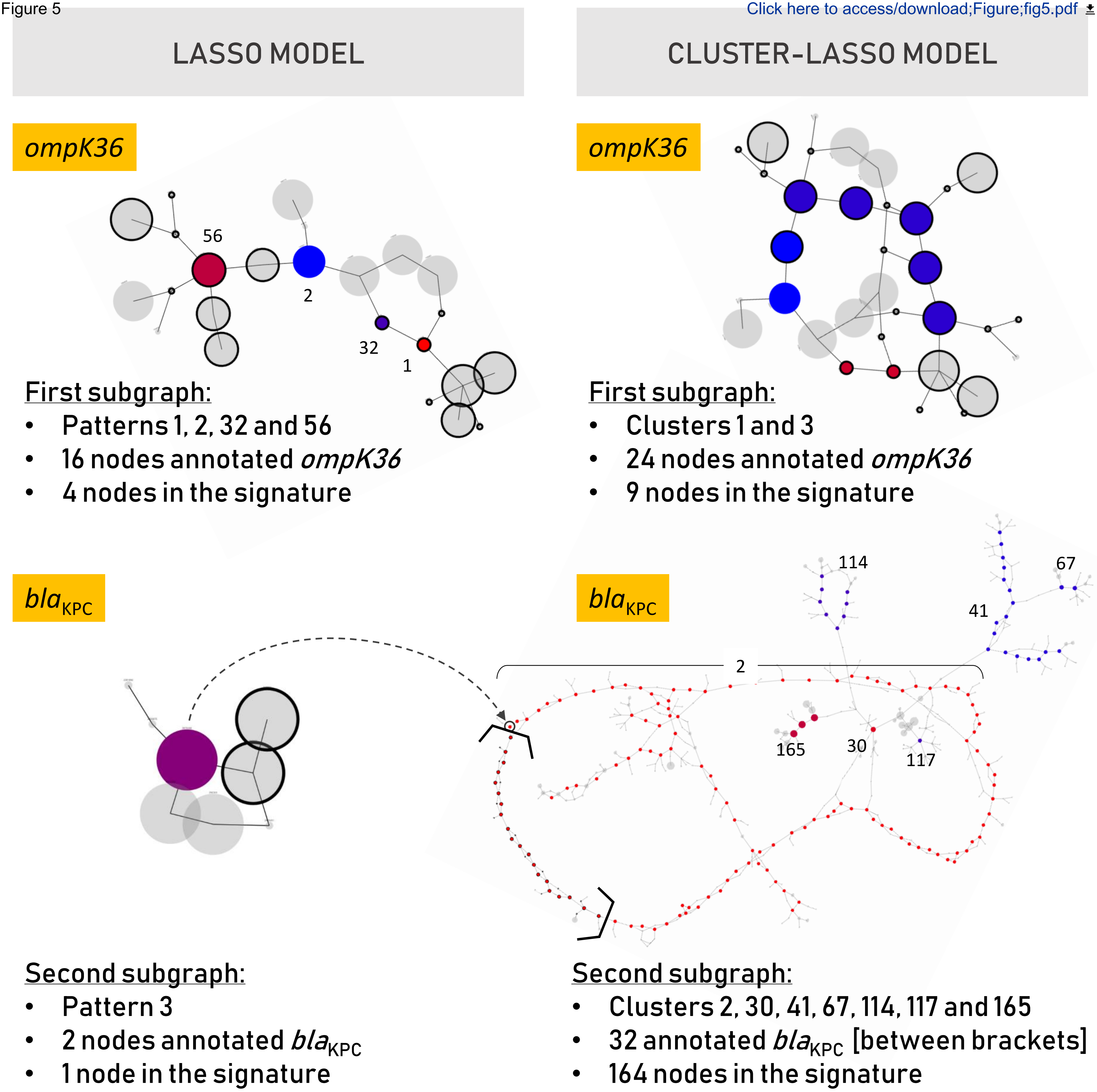

Figure 7

Click here to access/download;Figure;fig7.pdf

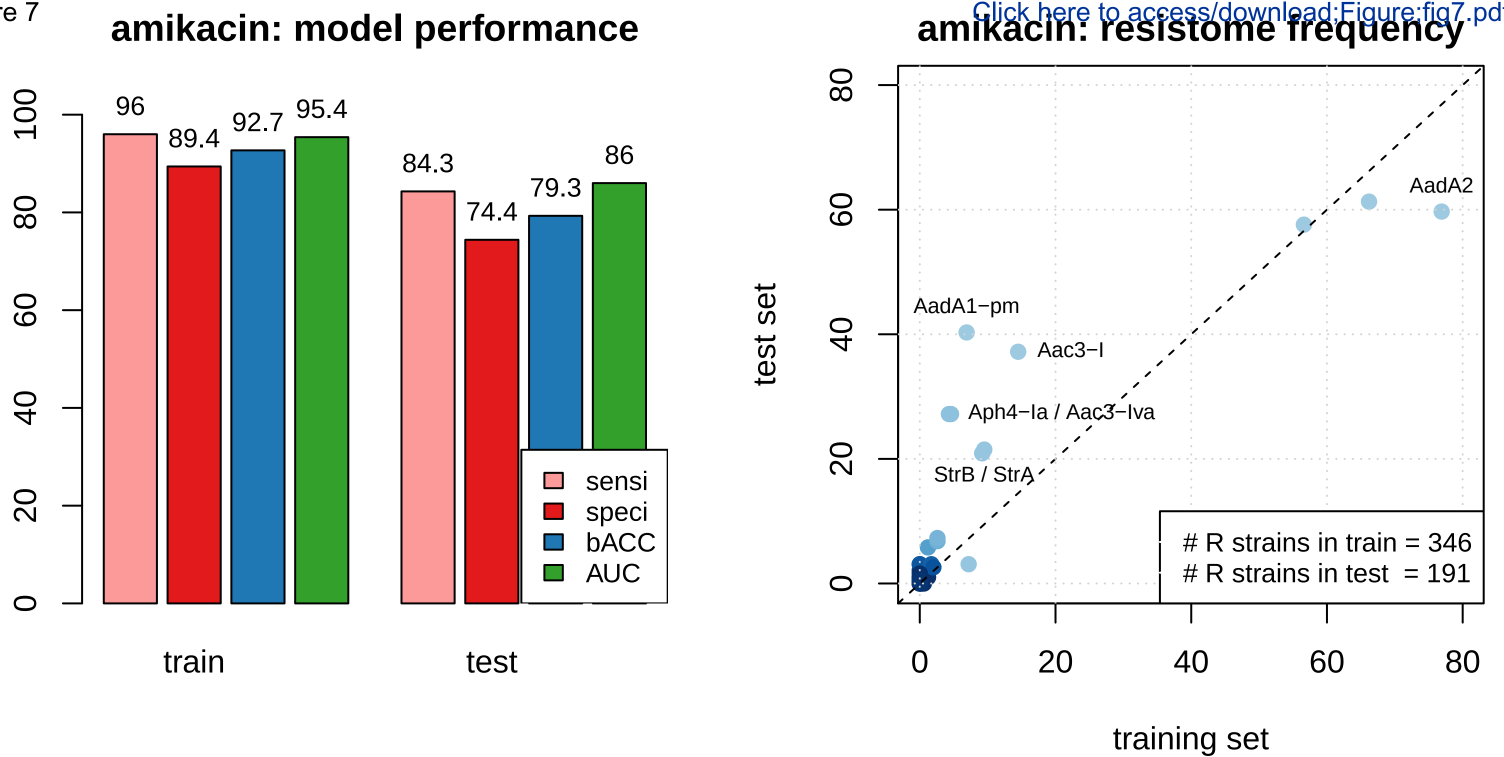

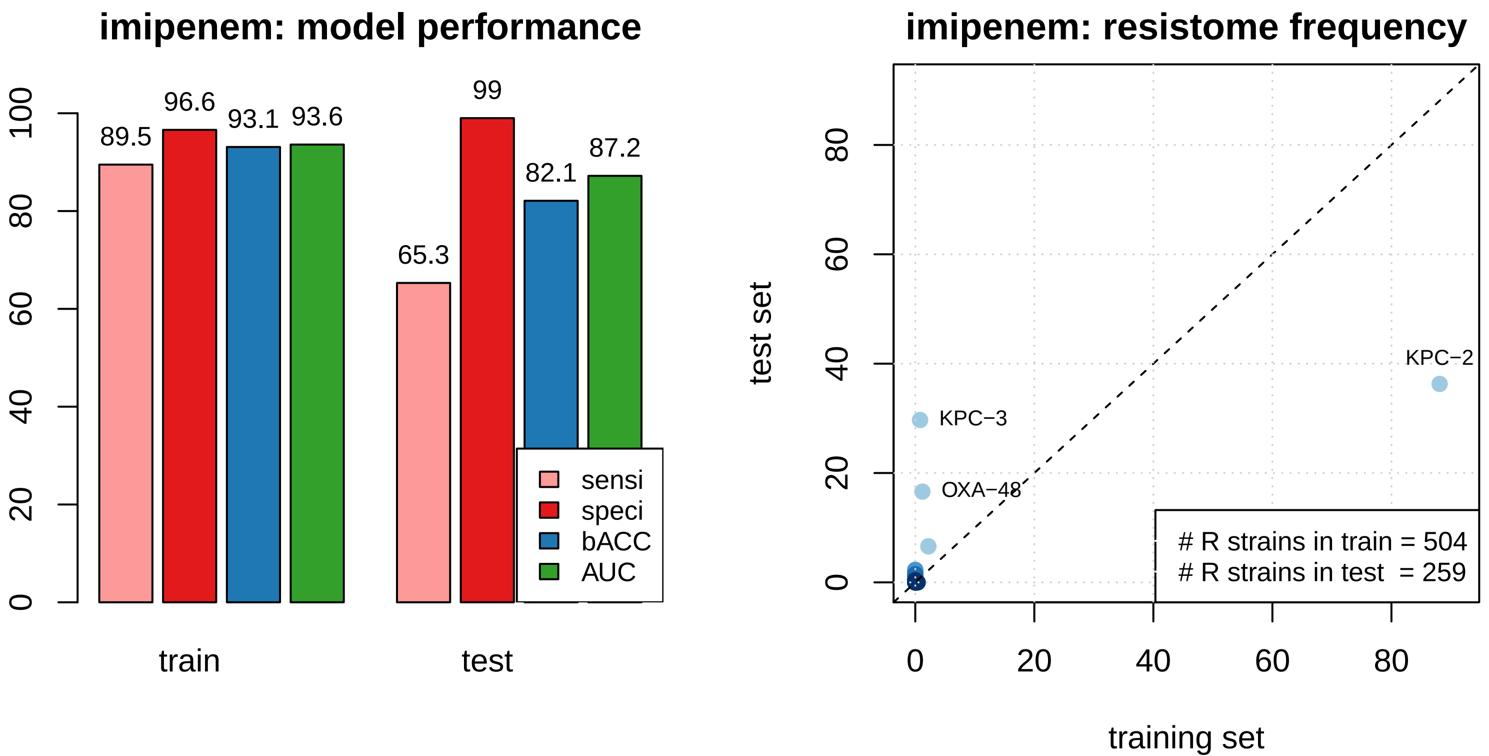

imipenem: model performance
89.5
96.6
93.1
93.6
65.3
99
82.1
87.2
0
20
40
60
80
100
train
test
sensi
speci
bACC
AUC
imipenem: resistome frequency
KPC−2
KPC−3
OXA−48
R strains in train = 504
R strains in test = 259
test set
training set
0
20
40
60
80

Figure 6

Click here to access/download;Figure;fig6.pdf

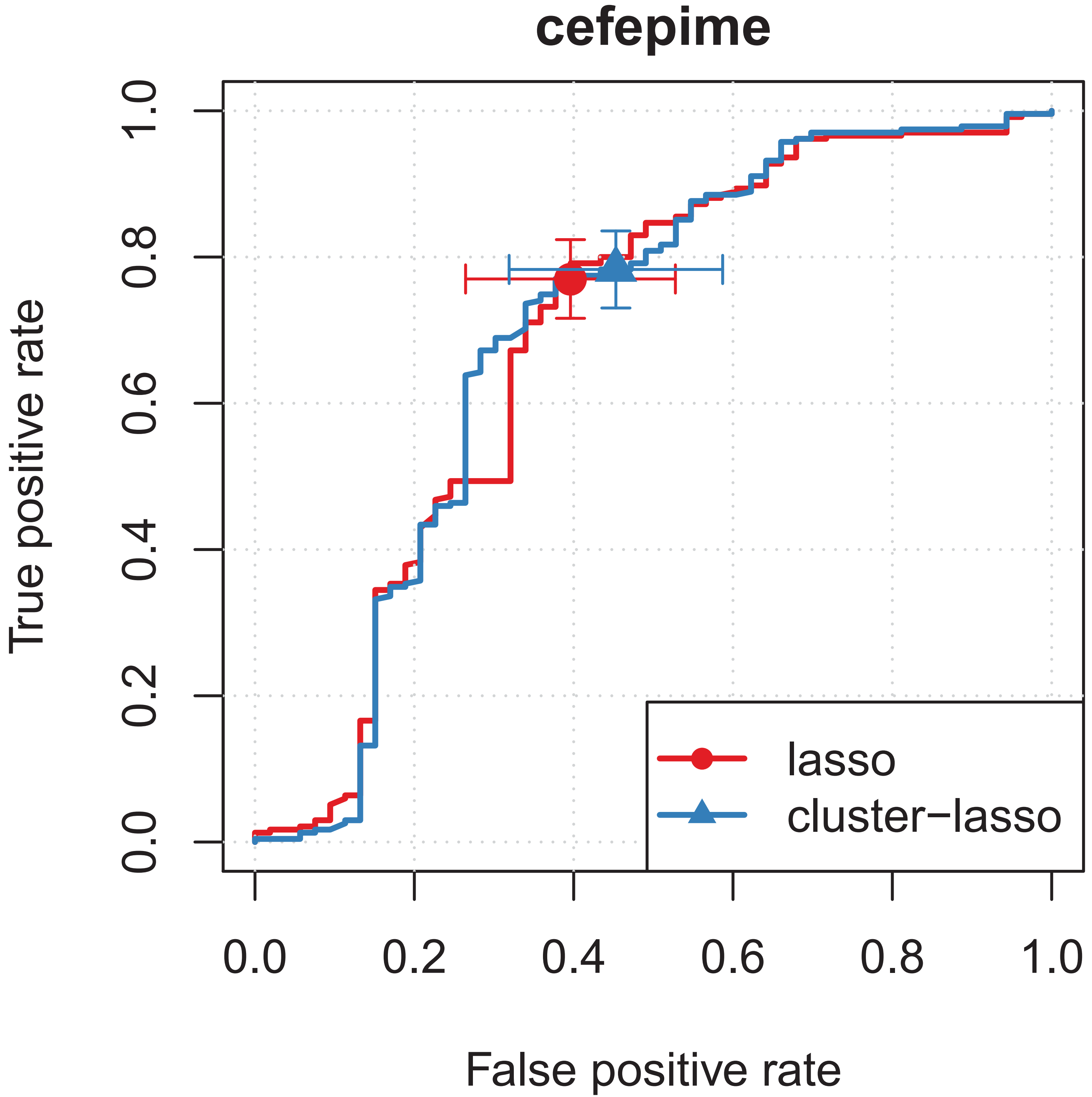

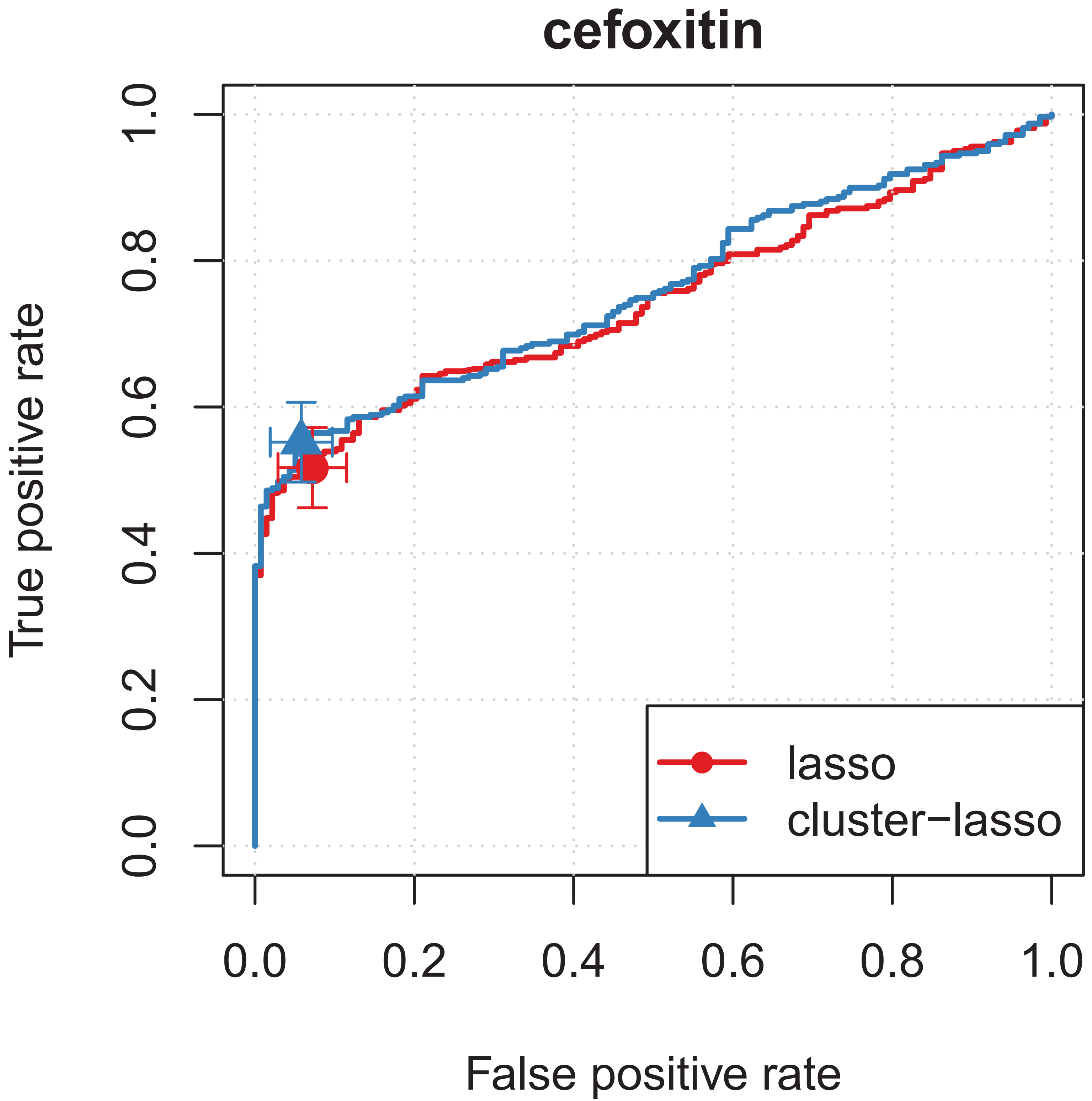

cefoxitin
True positive rate
False positive rate
0.0
0.2
0.4
0.6
0.8
1.0
lasso
cluster−lasso

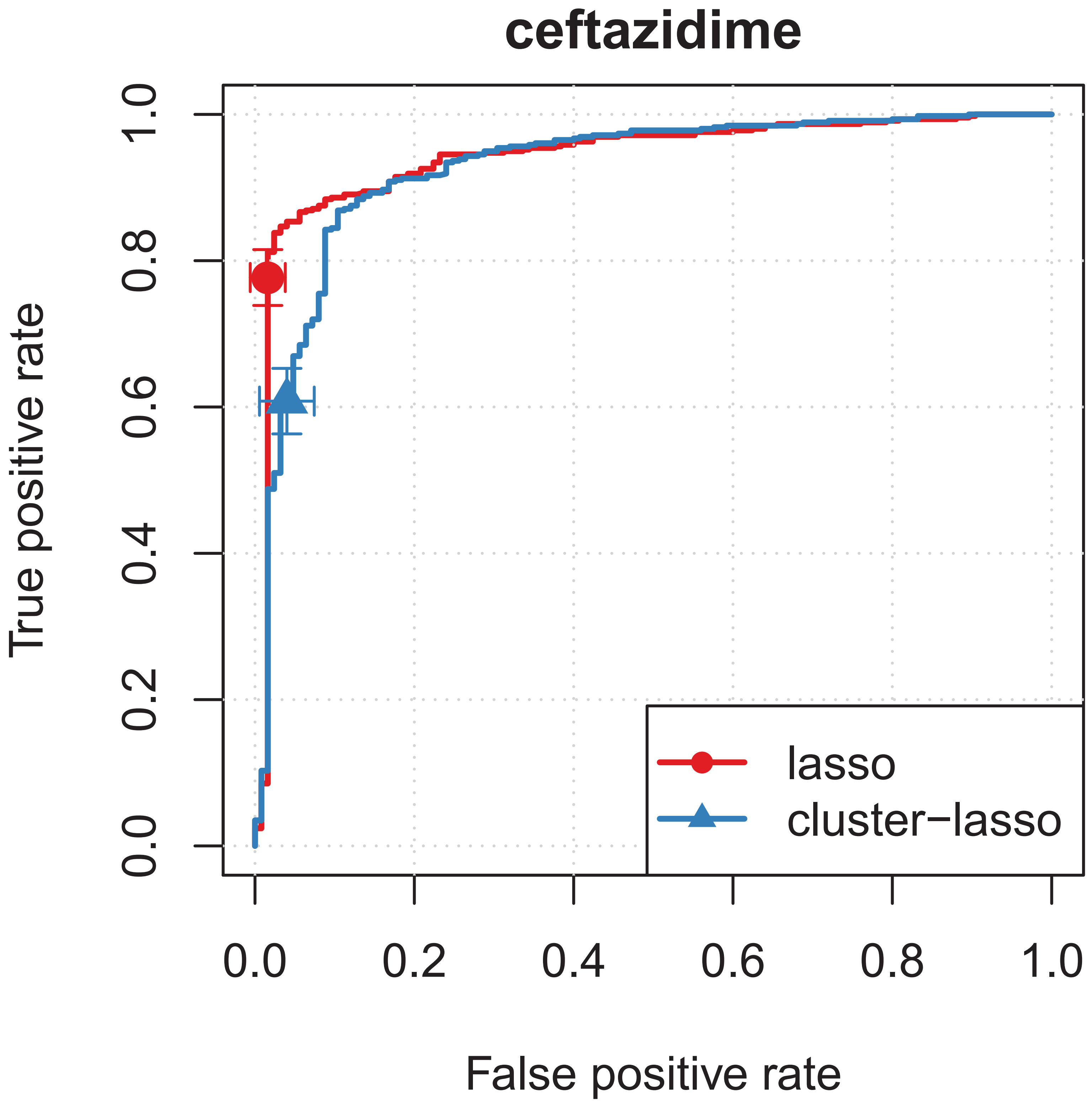
ceftazidime
True positive rate
False positive rate
0.0
0.2
0.4
0.6
0.8
1.0
lasso
cluster−lasso

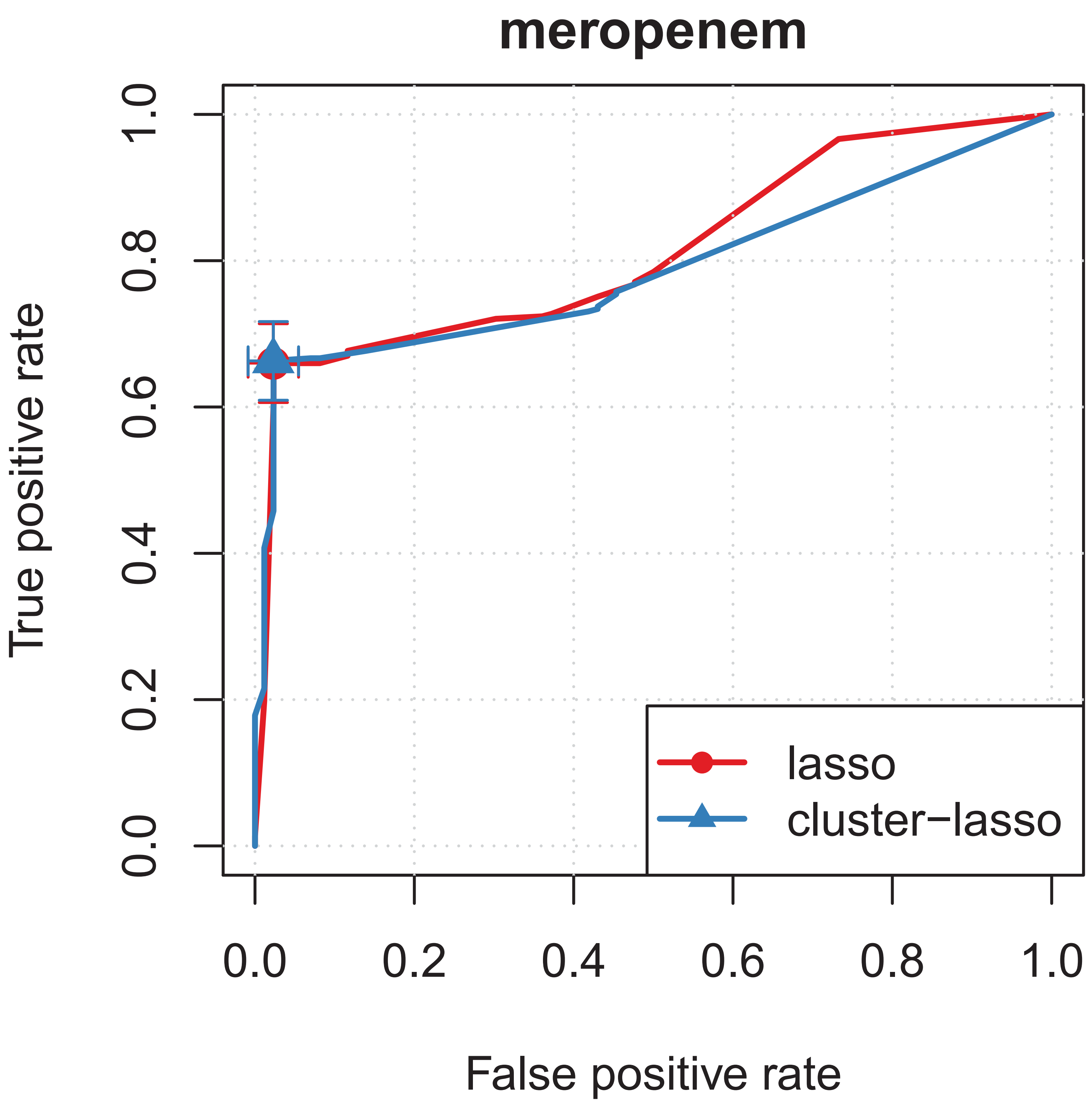
meropenem
True positive rate
0.0
0.2
0.4
0.6
0.8
1.0
False positive rate
0.0
0.2
0.4
0.6
0.8
1.0
lasso
cluster−lasso

Fig8
Click here to access/download;Figure;fig8.pdf

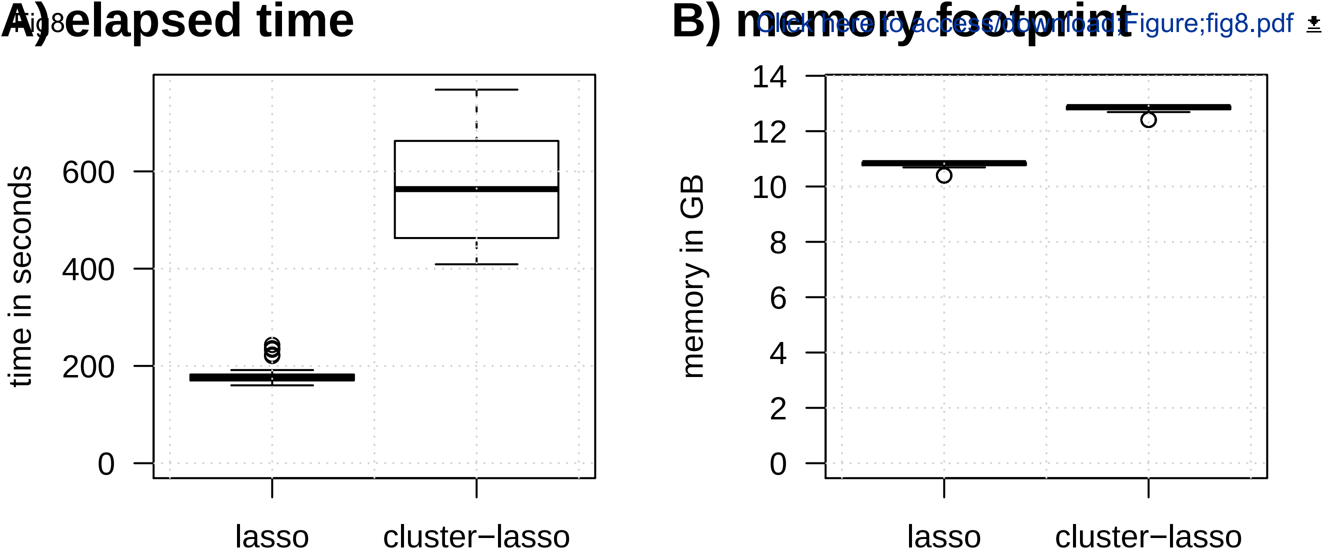

Answers to the reviewers

Click here to access/download
Supplementary Material
answers-to-reviewers.pdf

Main manuscript with red marks

Click here to access/download
Supplementary Material
main-with-red-marks.pdf

Click here to access/download
Supplementary Material
supplementary-data.pdf

Supplementary table : genome metadata

Click here to access/download
Supplementary Material
genomes_info.csv

Supplementary table : annotation results

Click here to access/download
Supplementary Material
KPN_SNS-cl_lasso-annot_signatures.xlsx

Supplementary table : annotation results (PDF format)

Click here to access/download

Supplementary Material

KPN_SNS-cl_lasso-annot_signatures.pdf
