# Supplementary material for: Interpreting k-mer–based signatures for antibiotic resistance prediction
Source: Gigascience. 2020 Oct 17;9(10):giaa110. doi: 10.1093/gigascience/giaa110 (PMC7568433; doi:10.1093/gigascience/giaa110)
Supplement: giaa110_Supplemental_Files [file giaa110_supplemental_files.zip › KPN_SNS-cl_lasso-annot_signatures.pdf]

Antibiotic	Support size	Feature id (ordered by model coefficient and grouped by DBGWAS subgraphs)	Model coefficient (absolute value)	Corresponding number of unitigs	Annotation (green = known resistant determinant, yellow = genetic element mobility, orange = ribosmal element)	Pathway/function (from annotation)	Type of variant (from DBGWAS visualisation)	Chr/plasmid (from annotation)	Nguyen *et al.*
amikacin	11	1	1.70	35	Tn3 family transposase	genetic element mobility	gene acquisition	plasmid	IncI1 plasmid conjugative transfer prepilin PilS
		2	0.63	2	cloacin	toxin, reduce competition from other bacteria		plasmid	
		3	0.34	1	diguanylate cyclase	involved in biofilm formation		plasmid	
		4	0.33	3	PqqF	zinc binding		chromosome	
		5	0.31	1	SDR family reductase	oxido-reduction		chromosome	
		6	0.28	36	mobile element protein	genetic element mobility	gene acquisition	plasmid	
		7	0.21	1	ANT(3'') aminoglycoside 3''-O-nucleotidyltransferase	aminiglycoside resistance gene	gene acquisition	plasmid	
aztreonam	28	1	0.56	8	N-actyltransferase	involved in the metabolism of xenobiotics, which can lead to both the inactivation of drugs and formation of toxic metabolites	local polymorphism	chromosome	Integron integrase IntI1
		2	0.48	3	recombinase	plasmid gene		plasmid	
		3,9,11,16,25	0.37	316	Tn3 (3), DUF3330 (9), CTX-M (11), CatB4 (16), mobile element protein (25)	plasmid with 2 resistance genes (CTX and CATB)	gene acquisition	plasmid	
		4	0.34	4	DNA-cytosine methyltransferase			plasmid	
		5	0.30	4	tRNA btw 16S and 23S	intergenic region, just after the 16S ribonomal RNA		chromosome	
		6	0.27	5	Tn3 family transposase	genetic element mobility		plasmid	
		7	0.25	2	SHV	Class A betalactamase		plasmid	
cefepime	34	1	0.69	84	CTX-M	Class A betalactamase	gene acquisition	chr/plasmid	Class A beta-lactamase (EC 3.5.2.6) => CTX-M family, extended spectrum
		2, 6	0.48	112	Tn3 (2), IS-like (6)	genetic element mobility	gene acquisition	chr/plasmid	
		3	0.46	1	Tn3 family transposase	genetic element mobility		plasmid	
		4	0.26	2	OmpK36	efflux pump - Mutant forms of the porin Omp36 result in reduced permeability to antibiotics.		chromosome	
		5	0.26	2	IS-like family transposase	genetic element mobility		chromosome	
		7	0.21	3	RNA-binding protein	Rop family plasmid primer RNA-binding protein		plasmid	
		8	0.21	11	hypothetical protein			plasmid	
		9	0.18	19	TEM	Class A betalactamase: cephalosporins	gene acquisition	plasmid	
cefoxitin	171	1, 3	1.55	9	OmpK36	efflux pump - Mutant forms of the porin Omp36 result in reduced permeability to antibiotics.	local polymorphism	chromosome	Class A beta-lactamase (EC 3.5.2.6) => KPC family, carbapenem-hydrolyzing
		2, 41, 67, 114, 117, 165	1.47	262	KPC (2), + plasmid sequences	Class A betalactamase: carbapenems, cephamycins	gene acquisition	plasmid	
		4, 12	0.53	10	MBL fold metallo-hydrolase	Protein family including class B beta-lactamases	local polymorphism in promoter	chromosome	
		5	0.48	1	intergenic region near IS element			chromosome	
		6	0.39	7	TranF	conjugative transfer		plasmid	
		7	0.38	2	hypothetical protein			chromosome	
		8	0.36	2	serine transporter	transporter		chromosome	
ceftazidime	43	1,2,8,9,16,22,24,37,43	0.85	540	CTX-M (1), Tn3 (2,8), IntI1 (9)	Class A betalactamase		plasmid	Integron integrase IntI1
		3,4,33	0.47	76	tRNA btw 16S and 23S	intergenic region, just after the 16S ribonomal RNA		chromosome	
		5	0.35	3	gpmB	phosphoglycerate mutase	local polymorphism	chromosome	
		6	0.33	6	glutamate decarboxylase			plasmid	
		7	0.32	2	cupin			chromosome	
		10,37	0.30	94	31-polyC // polyG regions	homopolymers - low complexity regions		no hit	
		11	0.29	6	TEM	Class A betalactamase: cephalosporins	gene acquisition	plasmid	
ciprofloxacin	35	1	3.02	5	gyrA	DNA gyrase, target of the antibiotic	local polymorphism	chromosome	Integron integrase IntI1
		1	3.02	3	parC	DNA gyrase, target of the antibiotic	local polymorphism	chromosome	
		2	0.67	12	IntI1	genetic element mobility	gene acquisition	plasmid	
		3, 8	0.64	264	QnrB (3), IS family transposase (8)	Quinolone resistance protein	gene acquisition	plasmid	
		4,5,6	0.52	54	Tn3 promoter (4), intergenic near integrase (5), AAC6'-Ib-cr5 (6)	Fluoroquinolone-acetylating aminoglycoside 6'-N-acetyltransferase	gene acquisition	plasmid	
		7	0.29	7	TraI	conjugative transfer relaxase/helicase		chr/plasmid	
		8.29	0.24	209	IS-like family transposase	genetic element mobility		chr/plasmid	
imipenem	7	1.5	4.27	196	KPC (1), + plasmid sequences	Class A betalactamase: carbapenems, cephamycins	gene acquisition	plasmid	Class A beta-lactamase (EC 3.5.2.6) => KPC family, carbapenem-hydrolyzing
		2	0.11	39	IS-like family transposase	genetic element mobility		plasmid	
		3	0.11	1	Tn3 family transposase	genetic element mobility		plasmid	
		4	0.03	4	transcriptional regulator	LuxR family transcriptional regulator		chromosome	
		6	0.02	1	hypothetical protein			plasmid	
		7	0.01	1	repB	plasmid replication initiator protein		plasmid	
meropenem	3	1	3.30	159	KPC, + plasmid sequences	Class A betalactamase: carbapenems, cephamycins		plasmid	Class A beta-lactamase (EC 3.5.2.6) => KPC family, carbapenem-hydrolyzing
		2	0.15	1	Tn3 family transposase	genetic element mobility		plasmid	
		3	0.14	4	HscA	molecular chaperone		chromosome	
piper.tazo	120	1,2,3,6,7,12,15,16,20,30,31 ,38,40,60,61,64,65,82,84,1 05,113	1.05	284	KPC (1), AAC3 (2), CatB4 (3), OXA (3), TEM (7)	plasmid of multi-resistance	gene acquisition	plasmid	plasmid stabilization system
		4.106	0.50	9	OmpK36	efflux pump - Mutant forms of the porin Omp36 result in reduced permeability to antibiotics.		chromosome	
		5	0.47	4	hypothetical protein			plasmid	
		8	0.43	3	intergenic region amyA	between alpha amylase and lipoprotein		chromosome	
		9	0.42	5	TrbI	conjugative transfer system protein		plasmid	
		10	0.40	3	MDR efflux	multidrug efflux RND transporter permease subunit OqxB		chromosome	
tetracycline	109	1,14,49,50,105	4.08	152	tetA (1), tetR (1)	Tetracycline resistance major facilitato superfamily MFS efflux pumps, Tetracycline transcriptional repressor	gene acquisition	plasmid	Tetracycline resistance regulatory protein TetR
		2	2.84	1	tetD	Tetracycline resistance major facilitator superfamily MFS efflux pumps		plasmid	
		3	0.40	8	tRNA btw 5S and 23S	intergenic region, just after the 23S ribonomal RNA		chromosome	
		4.33	0.39	9	TraK	conjugative transfer system protein		plasmid	
		5	0.34	2	BapA	BapA prefix-like domain-containing protein		chromosome	
		6	0.29	3	promoter of HokA	type I toxin-antitoxin system toxin HokA		chromosome	
		7	0.28	2	hypothetical protein			plasmid	
		8	0.26	3	OmpK36	efflux pump - Mutant forms of the porin Omp36 result in reduced permeability to antibiotics.		chromosome	
