# Supplementary material for: Interpreting k-mer–based signatures for antibiotic resistance prediction
Source: Gigascience. 2020 Oct 17;9(10):giaa110. doi: 10.1093/gigascience/giaa110 (PMC7568433; doi:10.1093/gigascience/giaa110)
Supplement: giaa110_Supplemental_Files [file giaa110_supplemental_files.zip › supplementary-data.pdf]

Interpreting kmer-based signatures for antibiotic resistance prediction - supplementary materials

Magali Jaillard, Mattia Palmieri, Alex van Belkum, Pierre Mahé

July 23, 2020

This document presents additional materials for the manuscript entitled *Interpreting kmer-based signatures for antibiotic resistance prediction*.

Contents

S1 Dataset constitution **2**

S2 Impact of screening and clustering thresholds **4**

S3 Cross-validation process of model selection **5**

S4 Detailed predictive performance **6**

S5 Interpretation of the models **7**

S6 ROC curves **11**

S7 Re-designing the dataset to evaluate the generalization ability of the models **12**

S8 Time and memory evaluation **13**

S9 Evaluation of an elastic-net stategy **14**

S10Evaluation of a cluster-level group-lasso stategy **17**

S11Impact of AST method on generalization **19**

S12Results obtained on other species **21**

S1 Dataset constitution

The training and test datasets considered in this study respectively involve 1665 genomes [Nguyen et al., 2018], and 634 genomes, including 114 strains from our bioMérieux collection (NCBI Bio-project PRJNA449293). Figure S1 represents the overall diversity of the strains according to their geographic origin, highlighting a strong bias towards American strains. Figure S2 further details the geographic origin as well as the antibiobic susceptibility method (AST) considered, according to the various sources considered: Nguyen et al. [2018] (used as training dataset), PATRIC and bioMérieux (used both as test dataset). We note that the AST methods differ between the training and test sets. Finally, Figure S3 shows the number of genomes available per sequence type (ST) in both the training and test databases, for the ST represented by at least 20 genomes. It reveals that the training dataset involves two main STs (ST307 and ST258), which have a much lesser prevalence in the test dataset. This is especially the case of ST307, which is represented by 594 strains in the training dastaset and only 4 in the test dataset.

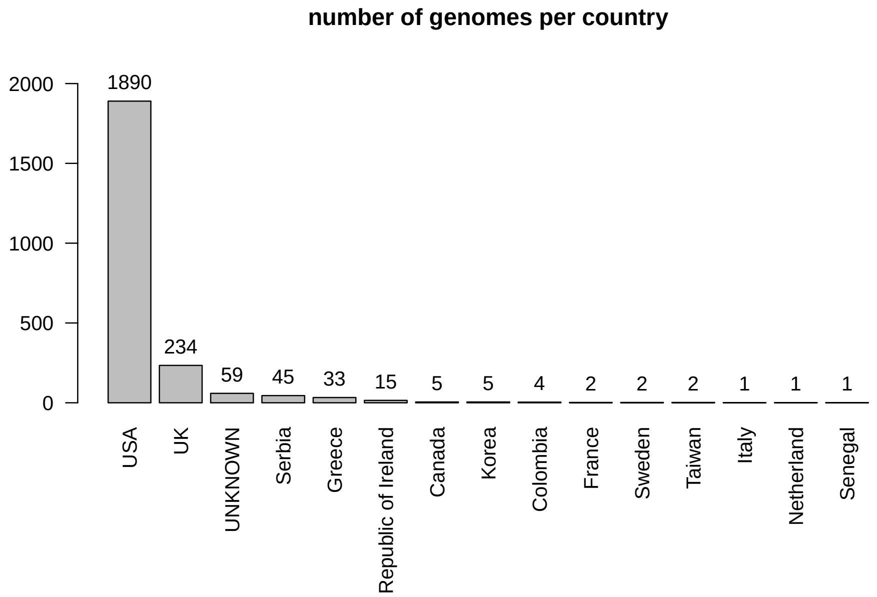

Figure S1: Number of genomes available per country

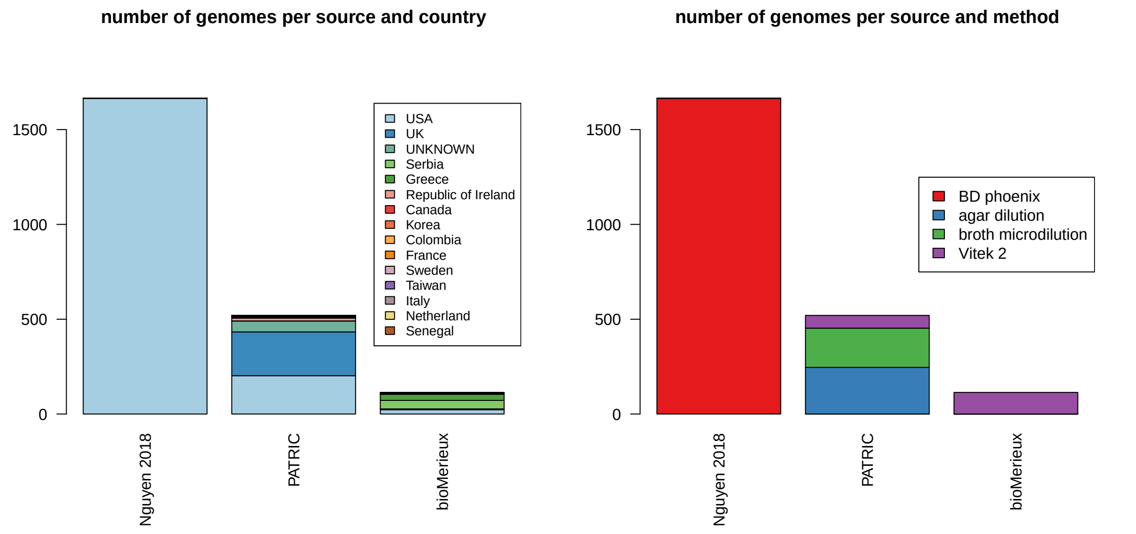

Figure S2: Number of genomes available per source, vs country (left) and antibiotic susceptibility method (right).

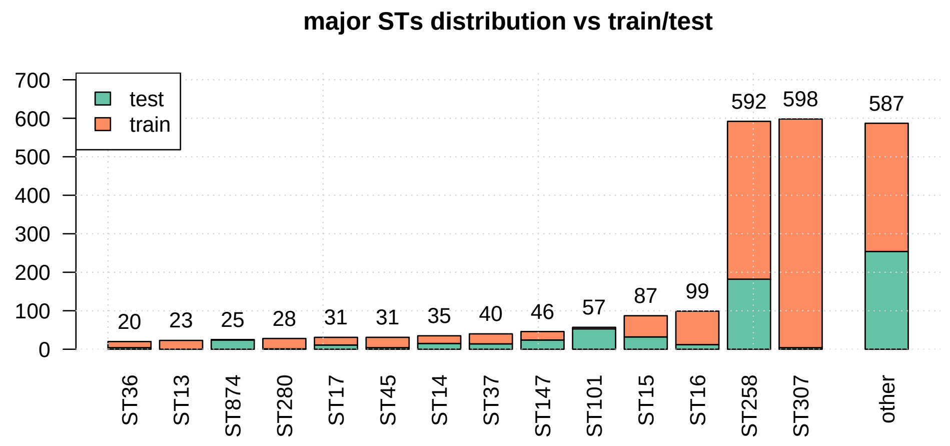

Figure S3: Number of genomes available per ST and train/test database, for the ST represented by at least 20 genomes.

S2 Impact of screening and clustering thresholds

Figure S4 illustrates the impact of the screening and clustering thresholds. A preliminary cross-validation study was set up to evaluate the impact of taking each of them in $\{0.9; 0.95\}$. As a limited impact was observed on both the accuracy and the support size of the models, these two thresholds were systematically set to 0.95 afterwards. Note that this cross-validation study was carried out in a slightly different setting, where strains of intermediate resistance were discarded from the dataset.

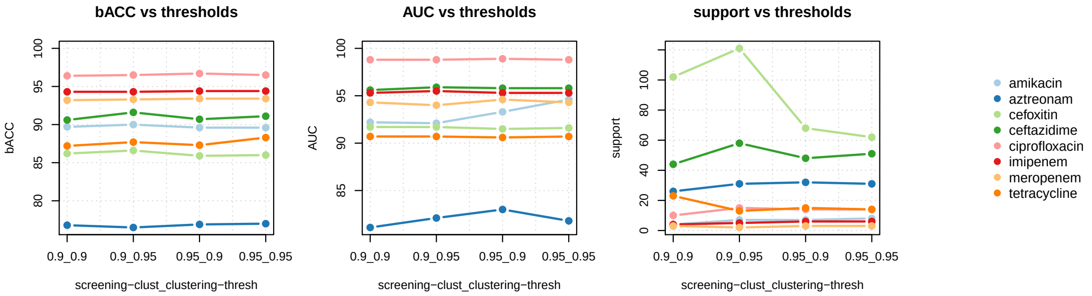

Figure S4: Cross-validation results - impact of considering different screening and clustering thresholds in terms of bACC (left), AUC (middle) and support size (right).

S3 Cross-validation process of model selection

Figure S5 illustrates the model selection process considered to optimize the regularization parameter involved in the final step of the cluster-lasso approach. The same process was applied to optimize the regularization of the standard lasso approach (omitting the screening and clustering steps).

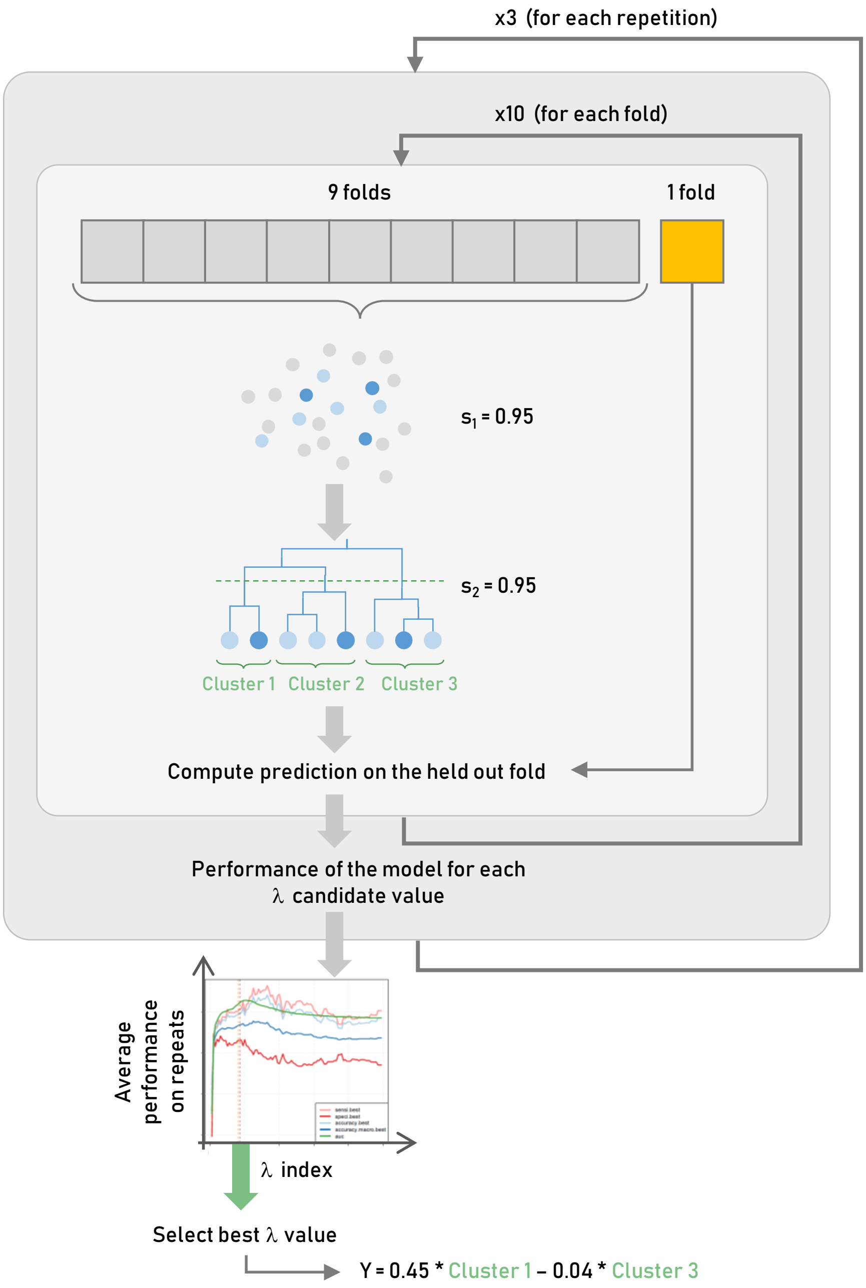

Figure S5: Illustration of the cross-validation process

S4 Detailed predictive performance

Table S1 provides further information about the predictive performance of the models estimated by cross-validation.

	lasso					cluster-lasso				
	sensi	speci	bACC	AUC	support	sensi	speci	bACC	AUC	support
amikacin	96	89.4	92.7	95.4	16	94.5	90.2	92.3	95.7	11
aztreonam	80.9	72.5	76.7	81.9	31	80.8	73.1	76.9	82.3	28
cefepime	77	71	74	80.4	53	75.6	71.6	73.6	79.8	34
cefoxitin	76.7	88.1	82.4	88.7	134	79	85.4	82.2	88.6	171
ceftazidime	91.3	91.9	91.6	95.8	51	89.5	91.9	90.7	95.3	43
ciprofloxacin	94.5	96.7	95.6	98.6	25	94.6	96.5	95.5	98.6	35
imipenem	89.5	96.6	93.1	93.6	10	89.9	95.5	92.7	93.4	7
meropenem	86.6	96.8	91.7	94	8	87	95.8	91.4	93.5	3
piper.tazo	80	83.1	81.6	89.6	127	77.3	85.7	81.5	89	120
tetracycline	77.9	88	83	88.5	181	75.9	89.8	82.9	87.7	109

Table S1: Cross-validation results - summary of performance.

S5 Interpretation of the models

Figure S6 (also available as an Excel file) gathers information allowing to interpret the main features involved in the cluster-lasso signatures.

This table is organized by antibiotic and highlights the 7 to 10 most important features involved in each signature, according to (the absolute value of) their model coefficients (4th column). The overall number of features involved in a given signature is reported in the 2nd column ("Support Size"). Each row of the table represents a `DBGWAS` subgraph built with a neighborhood of size 2 around the unitigs involved in the signatures, hence represents a particular genomic region of *K. pneumoniae* genomes [Jaillard et al., 2018]. We use the term "active unitigs" hereafter to refer to the unitigs involved in the signatures (as opposed to the other unitigs, which represent their genomic environment). When a subgraph gathers several features, their identifiers are separated by commas in the column "Feature id", and only the maximal value of their model coefficients is reported in the 4th column.

Visual inspection of the subgraphs allows to fill the column "Type of variant". When the topology does not allow a direct assignation between "gene acquisition" and "local polymorphism", the cell is left empty. The sequences of the active unitigs gathered in a subgraph are all used in a BLAST search for functional annotation (columns "Annotation" and "Pathway/function"). This search also allows to assess the origin of the variant (column "Chr/plasmid"), and we can observe that the type of variant "local polymorphism" is generally associated to chromosomic sequences while the "gene acquisition" are plasmidic. When the annotation points to a known antiobiotic resistance determinant (as defined by CARD, `https://card.mcmaster.ca/`), the cell is colored in green. When it refers to genetic element mobility (e.g., integron or transposon), the cell is yellow. Blue is used for annotations close to ribosomal RNA, and grey for hypothetical protein or low-complexity patterns.

For most antibiotics, expected resistance determinants are found with the highest model coefficient:

- The signatures built for both carbapenem drugs (imipenem and meropenem) are determined by the detection of the bla_{KPC} gene.
- The signatures of the 3rd and 4th generation cephalosporins (cefepime and ceftazidime) both include $bla_{\mathrm{CTX-M}}$ and bla_{TEM}. We note however that a higher model coefficient is attributed to $bla_{\mathrm{CTX-M}}$, which can be explained by the facts that $bla_{\mathrm{CTX-M}}$ has a higher prevalence than bla_{TEM} within the resistant strains of the training panel (64% vs 30%) and/or that $bla_{\mathrm{CTX-M}}$ leads to a higher MIC to cephalosporins than bla_{TEM} [Eliopoulos and Bush, 2001].
- The signature for cefoxitin (a cephamycin antibiotic) is mostly driven by the detection of a mutation of the *ompK36* efflux pump (see Figure S7) and by the detection of the bla_{KPC} gene: a resistant status is predicted if at least one of them is detected.
- The piperacillin-tazobactam drug, an association of a penicillin and an inhibitor, presents a signature where the detection of any of the resistance determinant listed in the column "Annotation" of Figure S6 will predict a resistant status. While the beta-lactamase (bla_{KPC}, bla_{OXA} and bla_{TEM}) and the mutation in *ompK36* are expected for antibiotics of the β-lactam family, the *Cat*B4 and AAC3 genes seem to have been selected because of their co-occurrence in the resistance plasmid carrying the β-lactamase genes.
- The signature found for aztreonam includes two plasmidic β-lactamase genes ($bla_{\mathrm{CTX-M}}$ and bla_{SHV}), which have however not been described to confer resistance to monobactams.

- Among the non-β-lactam drugs, the signature built for ciprofloxacin includes 4 different genuine markers (*gyrA*, *parC*, *QnrB* and AAC6'-Ib-cr5) with different associated coefficients: the detection of the highly correlated mutations in *gyrA* and *parC* have more importance in the signature than the presence of the plasmid-mediated *QnrB* and AAC6'-Ib-cr5 genes.
- The tetracycline resistance genes *tetA* and *tetD*, and the tetracycline transcriptional repressor *tetR* are the most important features in the tetracycline signature: the next features have far lower coefficient values.
- Finally, the amikacin signature presents one known marker of aminoglycoside resistance, the ANT(3") gene, however its detection alone is not sufficient to predict a resistant status: the gene needs be detected together with the transposase showing the highest coefficient, and other features selected in the model.

The level of biological interpretation we can reach thanks to the cluster-lasso strategy is far higher than the interpretation offered by other k-mer-based models. We report in the the last column "Nguyen et al." the annotations associated to Nguyen et al. [2018] signatures. This annotation was not obtained from the signature itself (the k-mers of size 8 were too short to be remapped), but from correlations computed between the phenotype (MIC values) and the presence or absence of antimicrobial resistance genes.

Figure S6: Cluster-lasso signatures: annotation and interpretation (Figure on next page).

Antibiotic	Support size	Feature id (ordered by model coefficient and grouped by DBGWAS subgraphs)	Model coefficient (absolute value)	Corresponding number of unitigs	Annotation (green = known resistant determinant, yellow = genetic element mobility, orange = ribosmal element)	Pathway/function (from annotation)	Type of variant (from DBGWAS visualisation)	Chr/plasmid (from annotation)	Nguyen *et al.*
amikacin	11	1	1.70	35	Tn3 family transposase	genetic element mobility	gene acquisition	plasmid	IncI1 plasmid conjugative transfer prepilin PilS
		2	0.63	2	cloacin	toxin, reduce competition from other bacteria		plasmid	
		3	0.34	1	diguanylate cyclase	involved in biofilm formation		plasmid	
		4	0.33	3	PqqF	zinc binding		chromosome	
		5	0.31	1	SDR family reductase	oxido-reduction		chromosome	
		6	0.28	36	mobile element protein	genetic element mobility	gene acquisition	plasmid	
		7	0.21	1	ANT(3'') aminoglycoside 3''-O-nucleotidyltransferase	aminiglycoside resistance gene	gene acquisition	plasmid	
aztreonam	28	1	0.56	8	N-actyltransferase	involved in the metabolism of xenobiotics, which can lead to both the inactivation of drugs and formation of toxic metabolites	local polymorphism	chromosome	Integron integrase IntI1
		2	0.48	3	recombinase	plasmid gene		plasmid	
		3,9,11,16,25	0.37	316	Tn3 (3), DUF3330 (9), CTX-M (11), CatB4 (16), mobile element protein (25)	plasmid with 2 resistance genes (CTX and CATB)	gene acquisition	plasmid	
		4	0.34	4	DNA-cytosine methyltransferase			plasmid	
		5	0.30	4	tRNA btw 16S and 23S	intergenic region, just after the 16S ribonomal RNA		chromosome	
		6	0.27	5	Tn3 family transposase	genetic element mobility		plasmid	
		7	0.25	2	SHV	Class A betalactamase		plasmid	
cefepime	34	1	0.69	84	CTX-M	Class A betalactamase	gene acquisition	chr/plasmid	Class A beta-lactamase (EC 3.5.2.6) => CTX-M family, extended spectrum
		2, 6	0.48	112	Tn3 (2), IS-like (6)	genetic element mobility	gene acquisition	chr/plasmid	
		3	0.46	1	Tn3 family transposase	genetic element mobility		plasmid	
		4	0.26	2	OmpK36	efflux pump - Mutant forms of the porin Omp36 result in reduced permeability to antibiotics.		chromosome	
		5	0.26	2	IS-like family transposase	genetic element mobility		chromosome	
		7	0.21	3	RNA-binding protein	Rop family plasmid primer RNA-binding protein		plasmid	
		8	0.21	11	hypothetical protein			plasmid	
		9	0.18	19	TEM	Class A betalactamase: cephalosporins	gene acquisition	plasmid	
cefoxitin	171	1, 3	1.55	9	OmpK36	efflux pump - Mutant forms of the porin Omp36 result in reduced permeability to antibiotics.	local polymorphism	chromosome	Class A beta-lactamase (EC 3.5.2.6) => KPC family, carbapenem-hydrolyzing
		2, 41, 67, 114, 117, 165	1.47	262	KPC (2), + plasmid sequences	Class A betalactamase: carbapenems, cephamycins	gene acquisition	plasmid	
		4, 12	0.53	10	MBL fold metallo-hydrolase	Protein family including class B beta-lactamases	local polymorphism in promoter	chromosome	
		5	0.48	1	intergenic region near IS element			chromosome	
		6	0.39	7	TranF	conjugative transfer		plasmid	
		7	0.38	2	hypothetical protein			chromosome	
		8	0.36	2	serine transporter	transporter		chromosome	
ceftazidime	43	1,2,8,9,16,22,24,37,43	0.85	540	CTX-M (1), Tn3 (2,8), IntI1 (9)	Class A betalactamase		plasmid	Integron integrase IntI1
		3,4,33	0.47	76	tRNA btw 16S and 23S	intergenic region, just after the 16S ribonomal RNA		chromosome	
		5	0.35	3	gpmB	phosphoglycerate mutase	local polymorphism	chromosome	
		6	0.33	6	glutamate decarboxylase			plasmid	
		7	0.32	2	cupin			chromosome	
		10,37	0.30	94	31-polyC // polyG regions	homopolymers - low complexity regions		no hit	
		11	0.29	6	TEM	Class A betalactamase: cephalosporins	gene acquisition	plasmid	
ciprofloxacin	35	1	3.02	5	gyrA	DNA gyrase, target of the antibiotic	local polymorphism	chromosome	Integron integrase IntI1
		1	3.02	3	parC	DNA gyrase, target of the antibiotic	local polymorphism	chromosome	
		2	0.67	12	IntI1	genetic element mobility	gene acquisition	plasmid	
		3, 8	0.64	264	QnrB (3), IS family transposase (8)	Quinolone resistance protein	gene acquisition	plasmid	
		4,5,6	0.52	54	Tn3 promoter (4), intergenic near integrase (5), AAC6'-Ib-cr5 (6)	Fluoroquinolone-acetylating aminoglycoside 6'-N-acetyltransferase	gene acquisition	plasmid	
		7	0.29	7	TraI	conjugative transfer relaxase/helicase		chr/plasmid	
		8.29	0.24	209	IS-like family transposase	genetic element mobility		chr/plasmid	
imipenem	7	1.5	4.27	196	KPC (1), + plasmid sequences	Class A betalactamase: carbapenems, cephamycins	gene acquisition	plasmid	Class A beta-lactamase (EC 3.5.2.6) => KPC family, carbapenem-hydrolyzing
		2	0.11	39	IS-like family transposase	genetic element mobility		plasmid	
		3	0.11	1	Tn3 family transposase	genetic element mobility		plasmid	
		4	0.03	4	transcriptional regulator	LuxR family transcriptional regulator		chromosome	
		6	0.02	1	hypothetical protein			plasmid	
		7	0.01	1	repB	plasmid replication initiator protein		plasmid	
meropenem	3	1	3.30	159	KPC, + plasmid sequences	Class A betalactamase: carbapenems, cephamycins		plasmid	Class A beta-lactamase (EC 3.5.2.6) => KPC family, carbapenem-hydrolyzing
		2	0.15	1	Tn3 family transposase	genetic element mobility		plasmid	
		3	0.14	4	HscA	molecular chaperone		chromosome	
piper.tazo	120	1,2,3,6,7,12,15,16,20,30,31,38,40,60,61,64,65,82,84,105,113	1.05	284	KPC (1), AAC3 (2), CatB4 (3), OXA (3), TEM (7)	plasmid of multi-resistance	gene acquisition	plasmid	plasmid stabilization system
		4.106	0.50	9	OmpK36	efflux pump - Mutant forms of the porin Omp36 result in reduced permeability to antibiotics.		chromosome	
		5	0.47	4	hypothetical protein			plasmid	
		8	0.43	3	intergenic region amyA	between alpha amylase and lipoprotein		chromosome	
		9	0.42	5	TrbI	conjugative transfer system protein		plasmid	
		10	0.40	3	MDR efflux	multidrug efflux RND transporter permease subunit OqxB		chromosome	
tetracycline	109	1,14,49,50,105	4.08	152	tetA (1), tetR (1)	Tetracycline resistance major facilitato superfamily MFS efflux pumps, Tetracycline transcriptional repressor	gene acquisition	plasmid	Tetracycline resistance regulatory protein TetR
		2	2.84	1	tetD	Tetracycline resistance major facilitator superfamily MFS efflux pumps		plasmid	
		3	0.40	8	tRNA btw 5S and 23S	intergenic region, just after the 23S ribonomal RNA		chromosome	
		4.33	0.39	9	TraK	conjugative transfer system protein		plasmid	
		5	0.34	2	BapA	BapA prefix-like domain-containing protein		chromosome	
		6	0.29	3	promoter of HokA	type I toxin-antitoxin system toxin HokA		chromosome	
		7	0.28	2	hypothetical protein			plasmid	
		8	0.26	3	OmpK36	efflux pump - Mutant forms of the porin Omp36 result in reduced permeability to antibiotics.		chromosome	

Cefoxitin signature: local polymorphism on *ompK36*

While DBGWAS allows easily to identify a local polymorphism with the *ompK36* gene as the first feature of the cefoxitin model, some further investigations including local multiple alignment are required to identify precisely the local mutation. Figure S7 present such an alignment, where two wild type sequences (JX310557 and JN128634.1) and two mutated sequences (JN128632.1 and MG577044.1) are aligned to the unitig sequences of the cluster-lasso and lasso signatures. Note that JN128632.1 and JN128634.1 are described in Novais et al. [2012]. In the lasso signature, features 2 and 56 map to the wild type and features 1 and 32 map to the sequences with the GGCGAC insertion. The minor-allele encoded patterns of features 1, 32 and 56 are correlated enough to be gather in a single cluster with the cluster-lasso, however individual sequence have opposite coefficient values. The lasso feature 2 is not as correlated because of a non-causal mutation happening 20 bps before the insertion, and is affected to a another cluster in the cluster-lasso signature.

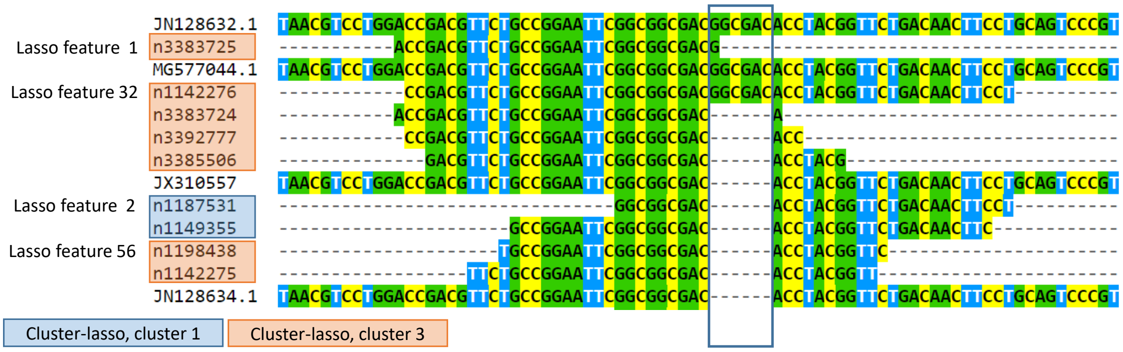

Figure S7: Multiple alignment of the unitigs annotated as *ompK36* in the cefoxitin signature.

S6 ROC curves

Figure S8 shows the ROC curves obtained on the test dataset for the drugs amikacin, aztreonam, ciprofloxacin, imipenem, piperacillin-tazobactam and tetracycline. The curves obtained for the four remaining drugs are shown in the main text. Note that the confidence intervals of the models sensitivities and specificities are defined for $\alpha = 0.05$ using the normal approximation of a binomial proportion.

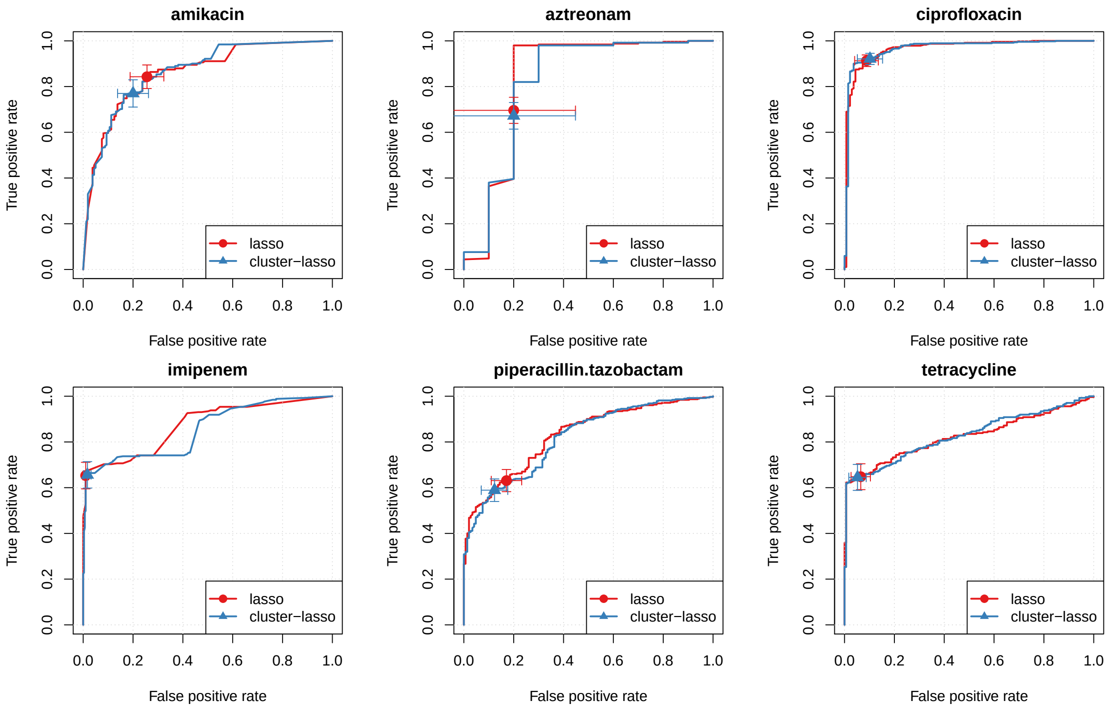

Figure S8: Test set results - ROC curves obtained for the drugs amikacin, aztreonam, ciprofloxacin, imipenem, piperacillin-tazobactam and tetracycline.

S7 Re-designing the dataset to evaluate the generalization ability of the models

To investigate the drop in the performance estimated by cross-validation and measured on the test set, we re-designed the dataset to obtain homogeneous training and test sets. For this purpose, we pooled the 1665 and 634 genomes that defined the original training and test sets, and randomly split the resulting 2299 genomes into three thirds, stratified by the strains sequence types (STs) determined by `kleborate`[1]. Two thirds were then used to define a training set of size 1519, and the remaining third was used as a test set of size 780. We then applied the exact same cross-validation based model selection process, and inferred the resistance phenotypes of the test strains.

Figure S9 compares the AUCs estimated by cross-validation and measured in the test datasets, using the original (left) and the re-designed (right) datasets. We note that the drop in performance observed with the original dataset is in general not observed anymore with the re-designed one. This is actually the case for all drugs but aztreonam, a drug for which the number of available S/NS phenotypes is strongly unbalanced (226 S vs 1676 NS, globally). The original training and test dataset respectively involved 216 and 10 susceptible strains for this drug. In the re-designed datasets, these numbers turned to 156 and 70. The drop in performance observed in the re-designed dataset may be due to an insufficient number of susceptible strains in the training data, and/or to an inaccurate performance estimation in the original test dataset, which was based on 10 strains only.

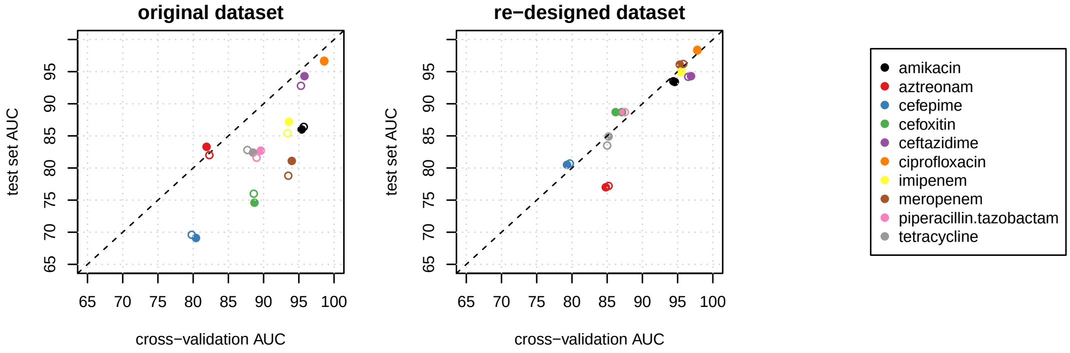

Figure S9: Test versus cross-validation performance using the original (left) and the re-designed (right) datasets. Each color corresponds to a drug and represents the AUC measured on the test set versus the AUC estimated by cross-validation. Solid and empty circles respectively correspond to lasso and cluster-lasso models.

[1] `https://github.com/katholt/Kleborate`

S8 Time and memory evaluation

To evaluate the computational requirements of the standard lasso and cluster-lasso procedures, we measured the time and memory required to compute a regularization path involving 100 values of the regularization parameter. For the standard lasso, this simply amounted to calling the `glmnet` function of the `glmnet` `R` package, using the variant matrix provided by `DBGWAS`. For the cluster-lasso procedure, this amounted to:

1. making the same call to `glmnet` to identify the set of active variables,
2. computing the $p_a \times p$ correlation matrix in order to identify the set of extended features,
3. building the clusters of correlated variables
4. making a second call to `glmnet`, using the variant matrix defined at the cluster-level.

This procedure was repeated five times for each drug, using a single Xeon E5-2690-V3 CPU. Figure S10 summarizes the results, illustrating the variability observed for each method and drug combination. We note that the while the time required by the lasso was relatively homogeneous across drugs, it was more variable for the cluster-lasso. This variability is essentially due to the fact that the lasso used in the first step of the procedure can identify a variable number of active variables, which directly impacts the time required to screen the remaining ones.

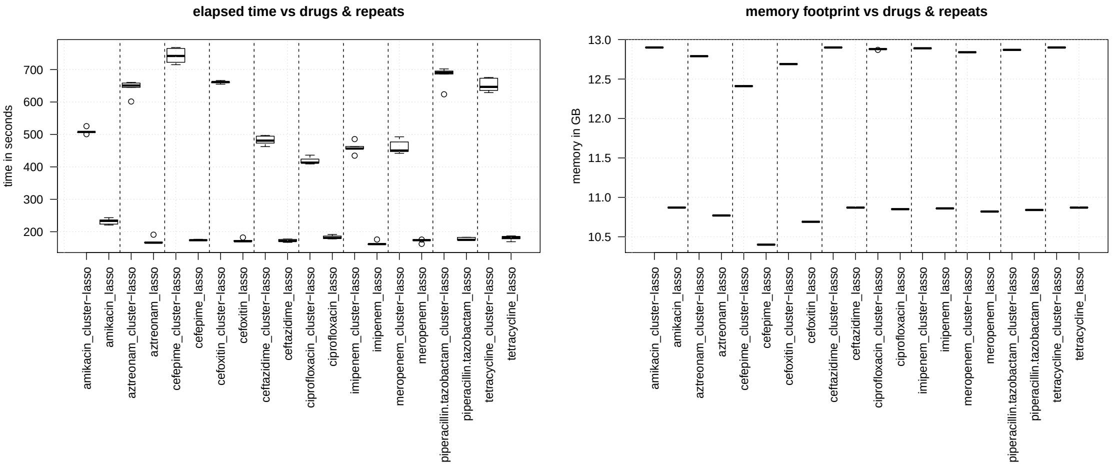

Figure S10: Time and memory requirements for the lasso and cluster-lasso procedures. Each boxplot corresponds to the values obtained for the 5 repetitions of the process described in Section S8.

S9 Evaluation of an elastic-net stategy

The elastic-net penalty is defined as $\Omega(\beta) = \frac{1-\alpha}{2}||\beta||_2^2 + \alpha||\beta||_1$. It therefore achieves a trade-off between the lasso and ridge penalties, respectively obtained for $\alpha = 1$ and $\alpha = 0$, which is known to stabilize the lasso solution and achieve a "grouping" of correlated variables [Zou and Hastie, 2005]. To evaluate how relying on the elastic-net penalty compares to the lasso and cluster-lasso strategies, we considered α values taken in $\{1; 0.99; 0.98; 0.9; 0.7; 0.5; 0.01\}$. We built a model for each value of α considered, relying on the cross-validation procedure described in the main text to optimize the regularization parameter. The then evaluated the predictive performance and analyzed the signatures obtained when α varied.

Figure S11 summarizes the cross-validation results obtained. It shows, for each drug, the evolution of the balanced accuracy, the AUC and the support size of the model as a function of α. While, as expected, the support size gradually increased as α decreased, we noted that the performance remained steady across the range of α values considered. This therefore means that this parameter cannot be optimized in terms of predictive performance only, making it intrinsically hard to optimize objectively.

We then aimed to compare the signatures obtained by the elastic-net, lasso and cluster-lasso strategies. For this purpose, we considered the meropenem and ceftazidime antibiotics and α values of 1, 0.9 and 0.7. We focused on these drugs since they were already studied in the main text, but similar observations were made for the other drugs. Results are shown in Figure S13, using the representation considered in Figure 3 of the main text, namely a correlation matrix restricted to the `DBGWAS` patterns involved in the various signatures. When $\alpha = 1$, the lasso and elastic-net signature coincided hence, as extensively discussed in the main text, led to a partial reconstruction of the underlying genomic determinants. As α was decreased to 0.9 and 0.7, we noted that an increased number of patterns was indeed selected among the major clusters identified by the cluster-lasso strategy. Even when $\alpha = 0.7$, however, these clusters were only partly reconstructed. In the case of meropenem for instance, roughly half of the patterns defining the major cluster identified by the cluster-lasso were involved in the elastic-net signature obtained for $\alpha = 0.7$. This fraction seemed to be even lesser for most of the clusters of the ceftazidime signatures (e.g., in the 2nd and 7th ones, shown in blue and pink respectively, that appear on the top-right and bottom-left of the correlation matrix). Importantly moreover, the weights associated to these highly correlated patterns were not homogeneous, as can be seen from the corresponding color bars in Figure S13, although they intrinsically have a similar predictive power, because of their strong correlation. This therefore means that while the elastic-net is indeed able to retrieve some patterns strongly correlated with that identified by the lasso, it fails to identify many of them and does not lead to homogeneous weights among strongly correlated features, hence does not reflect the fact that they bear the same predictive power. Together with the fact that an objective criterion is lacking to optimize the α parameter, we believe it is less powerful to identify interpretable signatures than the cluster-lasso, which leads to explicit groups and a common weight to reflect their predictive power.

Finally, Figure S12 shows the main genomic feature identified by `DBGWAS` for the various meropenem signatures, illustrating the partial reconstruction of the *blaKPC* gene and the variability of the weights associated to the unitigs.

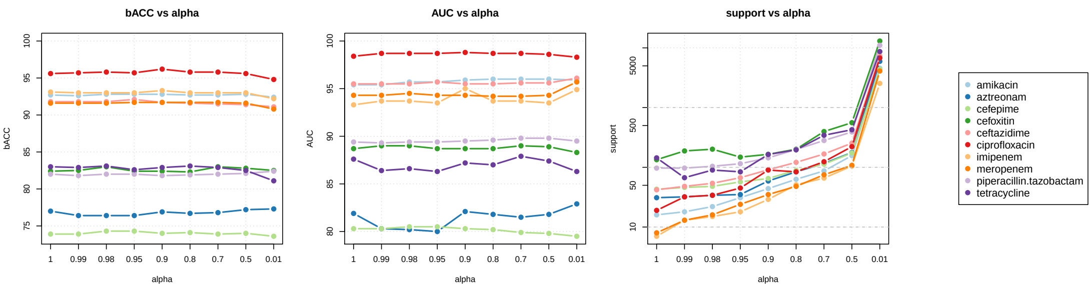

Figure S11: Cross-validation results based on the elastic-net penalty - impact of the α in terms of bACC (left), AUC (middle) and support size (right).

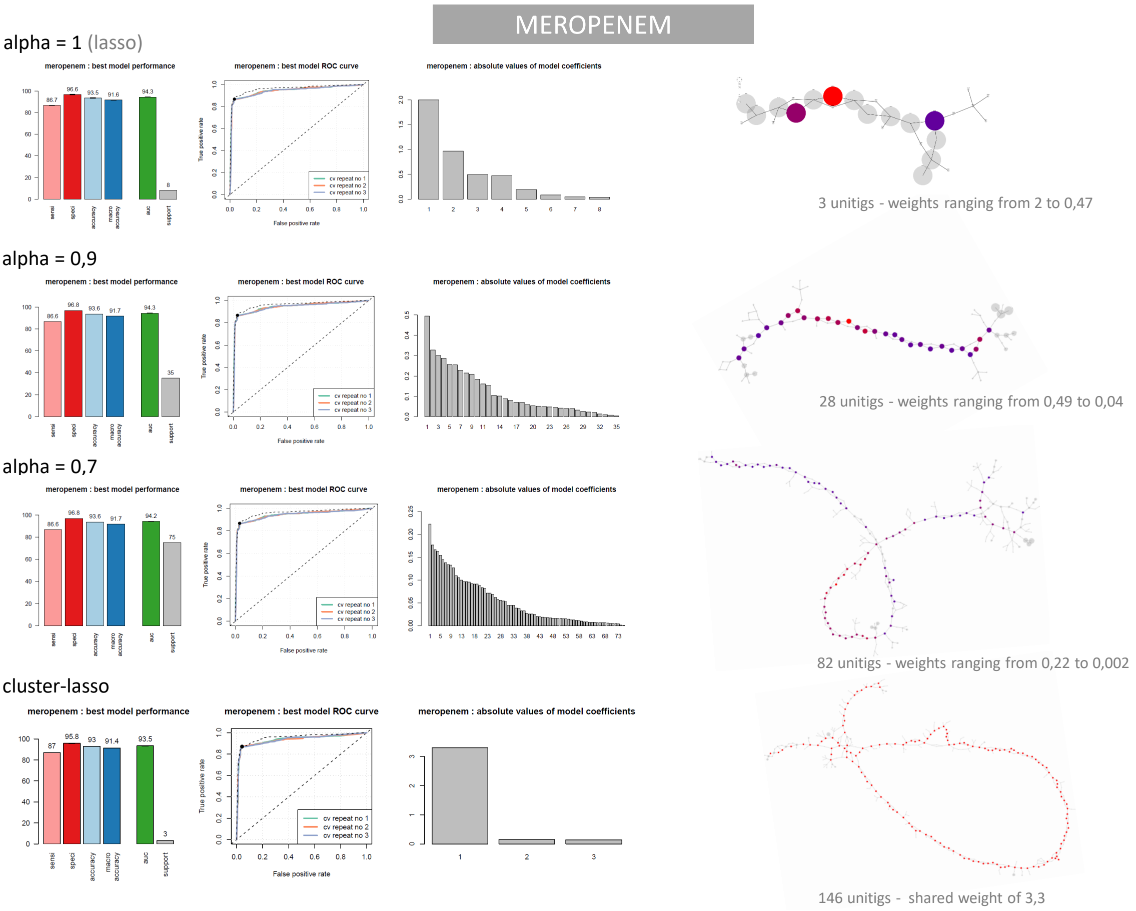

Figure S12: `dbgwas` visualization of the main genomic feature identified in the meropenem signatures obtained using the lasso (top), the elastic-net penalty for $\alpha = 0.9$ and $\alpha = 0.7$ (middle), and the cluster-lasso (bottom)

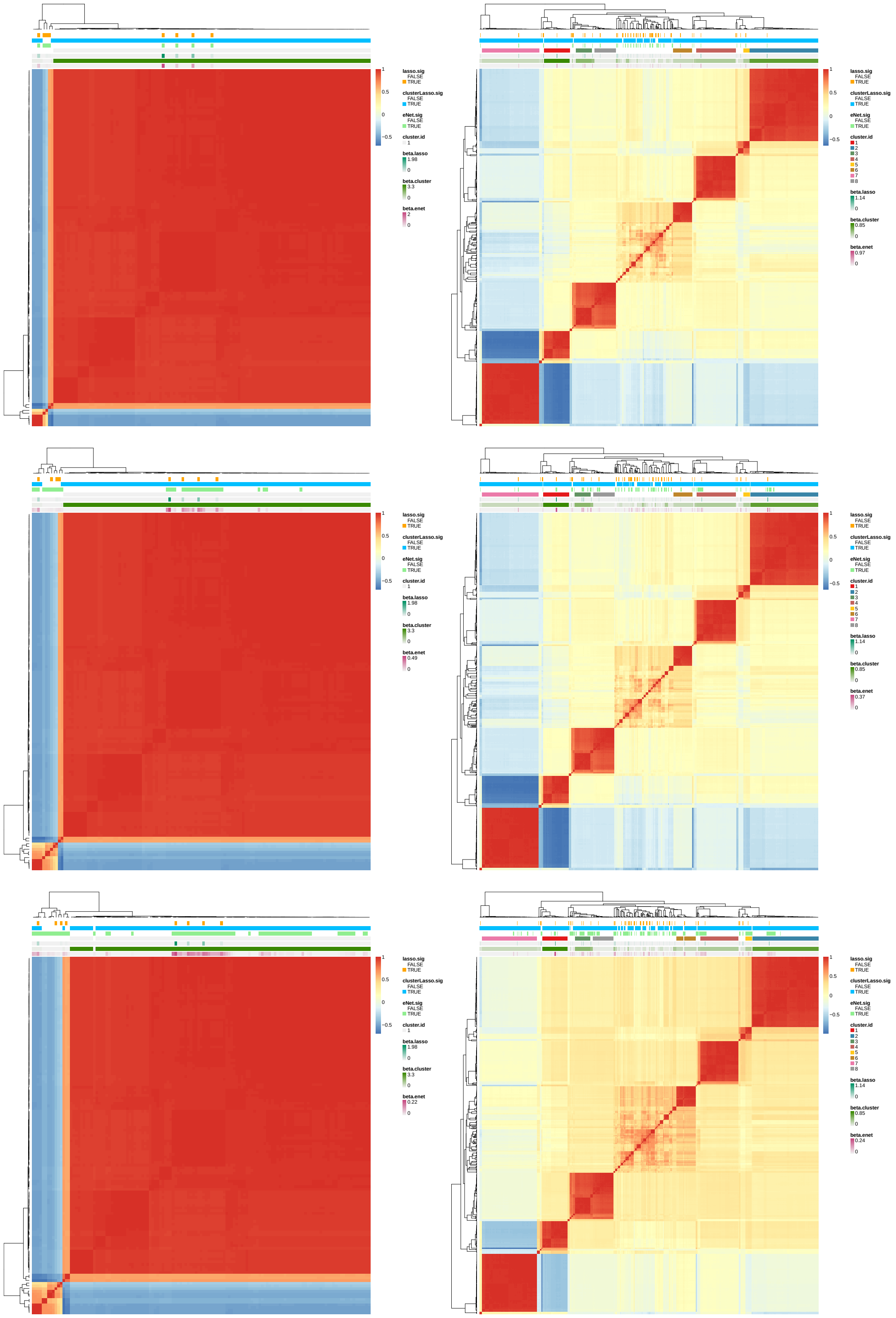

Figure S13: Illustration of the meropenem (left) and ceftazidime (right) signatures obtained using the elastic-net penalty for $\alpha = 1$ (top), $\alpha = 0.9$ (middle) and $\alpha = 0.7$ (bottom). The three color bars shown above the correlation matrix indicate which signatures involve the various patterns. The three color bars shown on the bottom represent the corresponding weights.

S10 Evaluation of a cluster-level group-lasso stategy

In order to evaluate the impact turning to a cluster-level group-lasso strategy instead of merging clusters of correlated variable into single composite variables, we considered the following procedure. For each drug of the panel, we applied the first two steps of our approach:

- screening the variables: identifying the "active" ones by the lasso, and retrieving the "extended" ones, according to a screening threshold $s_1 = 0.95$.
- defining clusters of correlated variables by bottom-up agglomerative clustering, cutting the resulting dendrogam according to a clustering threshold $s_2 = 0.95$.

Instead of subsequently summarizing each cluster to a single composite variable, however, we then resorted to a group-lasso approach, using the identified clusters as groups and the `gglasso` package[2] [Yang and Zou, 2015].

Figure S14 illustrates the models obtained for amikacin, cefepime, ceftazidime and meropenem. We focus on these drugs for the sake of readability because their supports are relatively limited (3 to 43 clusters for meropenem and ceftazidime, respectively), but similar observations were made for the other drugs. For each drug, we represent the group-lasso solution obtained when the number of active groups/clusters corresponded to the support obtained by our approach (i.e., the support reported in Table 2 of the main text), and consider the following indicators :

- First, the sum of the weights observed in each group/cluster, which represents the overall weight of the clusters. For meropenem for instance, we note that these weights are very similar to that shown in Figure 4 of the main text.
- Second, the number of active and inactive variables within each (active) group/cluster. This figure is essentially a sanity check, and allows to check that every member of a group is indeed active when the group itself is active.
- Finally, the distribution of the weights obtained within each group/cluster. Each boxplot corresponds to a given group and shows the distribution of the weights affected to its constituting variables. The red dot shown on top of each boxplot corresponds to the average weight within the cluster. It therefore corresponds to the weight that is implicitly applied to each member when we summarize each cluster by an average variable, as proposed in this work. We note that the variability of the weights within a given group/cluster is in general so small that each weight basically corresponds to the average weight observed within the cluster. This is almost systematically the case, except for a couple of features in the ceftazidime model, for which some level of variability is observed.

This analysis therefore reveals that the cluster-level group-lasso approach almost systematically affects to each member of a given cluster a weight that is very close to the average weight within the cluster. This therefore means that resorting to a cluster-level group lasso is essentially equivalent to summarizing each cluster to an average composite variable and relying on a standard lasso. We emphasize, however, that this is especially true because we enforce a high-level of correlation between the groups, by considering high-values of the clustering threshold s_2. Lowering this value would lead to a greater heterogeneity within the clusters/groups, hence a greater variability of the weights.

[2] `https://cran.r-project.org/web/packages/gglasso/index.html`

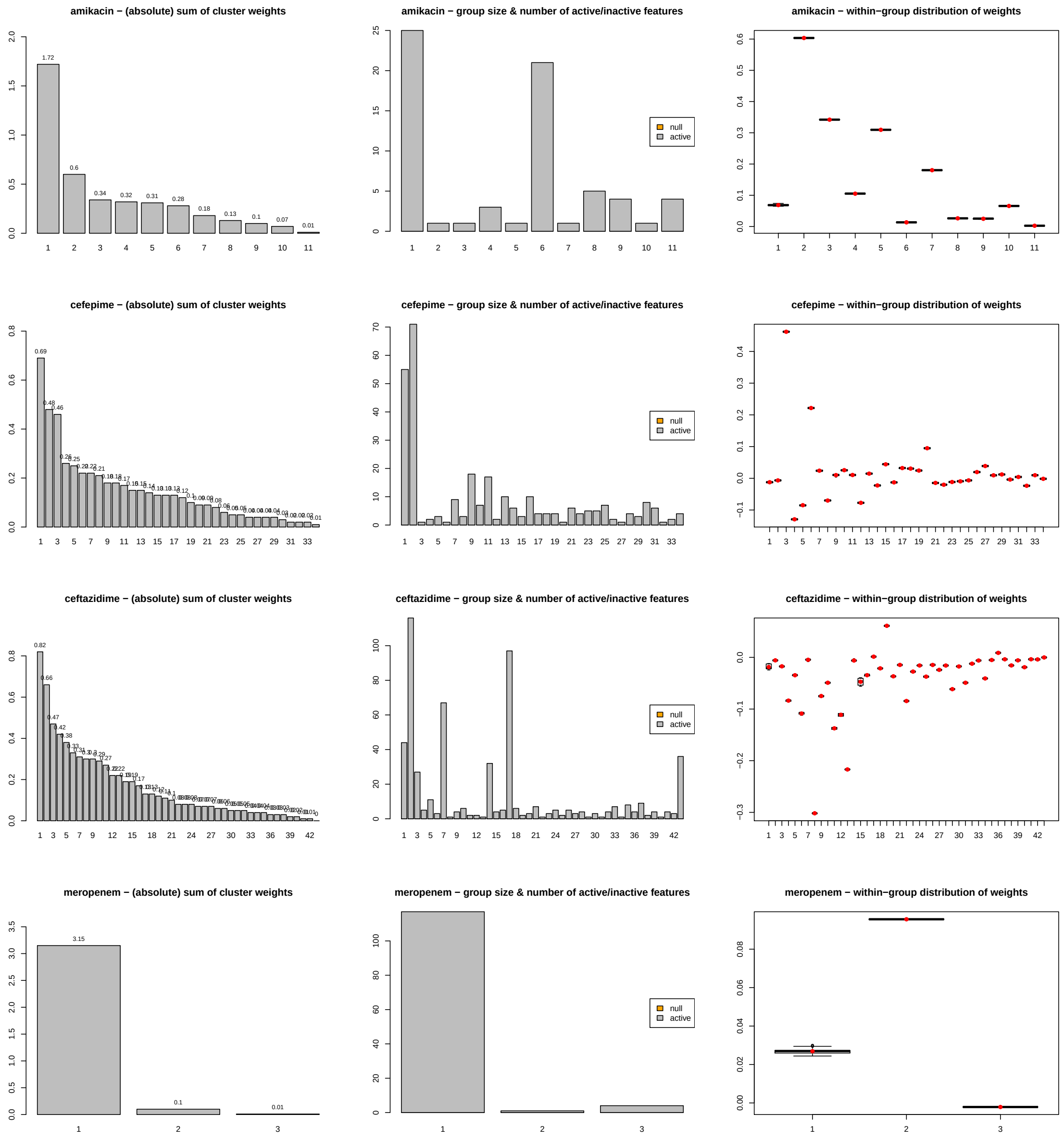

Figure S14: Cluster-level group-lasso models obtained for the drugs amikacin, cefepime, ceftazidime and meropenem, when the number of active groups corrresponds to the support obtained by the cluster-lasso. Left: sum of the weights observed in each group/cluster. Middle: group/cluster size versus number of active/inactive variables. Right: distribution of the weights obtained within each cluster. Refer to the text (Section S10 for more details.

S11 Impact of AST method on generalization

As shown in Figure S2, the phenotypic antibiotic susceptibility testing (AST) methods used to define the reference MICs differed between the training set (which involved the Phoenix technology (Becton Dickinson, Franklin Lakes, USA) only) and the test test (which was based on agar dilution, broth microdilution or VITEK 2). This constitutes a potential limitation of the current study, as AST is notoriously subject to a high level of technical variability [Brennan-Krohn et al., 2017], which intrinsically brings noise to the reference labels used to train and validate supervised ML models. In this section, we aimed to assess whether a ML model learned from data provided by a given AST method (in this case, BD Phoenix) will generalize to data provided by an alternative AST method. For this purpose, we proceeded as follows :

1. We first extracted from the original training set a subset of 200 genomes to include in the test set. This therefore led to a test set made of 834 genomes, within which around 200 genomes were characterized by each AST technology (BD Phoenix: $n = 200$, agar dilution: $n = 246$, broth microdilution: $n = 207$ and VITEK 2: $n = 181$). Of note, as the genomic diversity is smaller within the original training set than between the original training and test sets, we did not select these 200 genomes randomly, but aimed to achieve a similar distribution of genomic distance to the training set as the one of the original test set. We relied for this purpose on the mash distance [Ondov et al., 2016] to quantify the distance between two genomes, and selected the 200 genomes so that the mash distance to their closest genome in the training set follows the same distribution as that observed on the test set. This therefore allowed to evaluate the impact of the AST method on subsets of strains having a comparable genomic heterogeneity with respect to the training set, as illustrated in Figure S15.
2. When then fitted lasso-penalized logistic-regression models for the 10 drugs based on the $1665 - 200 = 1445$ remaining genomes, using the process described in the main text and illustrated in Figure S5.
3. We finally evaluated the predictive performance of these models on the test set according to the various AST methods, and tested whether significantly better performance were obtained on the fraction of genomes characterized by the BD Phoenix technology. We considered the AUC performance indicator, and tested whether the AUC difference between BD Phoenix and each alternative AST method was significantly different from 0, using a bootstrap procedure implemented in the `pROC` `R` package [Robin et al., 2011].

Table S2 shows the AUC measured on the test set for each AST method, as well as the p-values obtained by testing whether the AUC obtained on strains characterized by agar dilution, broth-microdilution or VITEK 2 are significantly different (worse or better) than the AUC obtained on strains characterized by BD-Phoenix. We also report the number of susceptible (S) and non-susceptible (NS) strains available for each drug and AST method. We can made the following observations:

- No significant difference in AUC are observed between BD-Phoenix and broth-microdilution.
- A significantly lesser performance is obtained on strains characterized by agar-dilution for cefoxitin and piperacillin-tazobactam, suggesting indeed that the models learned from Phoenix data fail to generalize for these drugs and this AST technology. Interestingly however, significantly better AUC are obtained for these two drugs on strains characterized by VITEK 2. Figure S16 (top) illustrates this phenomenon for cefoxitin.
- Significant differences are observed for 6 drugs out out 10 when strains are characterized by VITEK 2. We note however that better performance are actually obtained for 4 of these

6 drugs (cefoxitin, ciprofloxacin, piperacillin-tazobactam and tetracycline), and that lesser performance are only obtained for cefepime and meropenem, as illustrated in Figure S16 for meropenem.

In conclusion, while some effects were indeed observed, no systematic detrimental effect was observed when switching to a different AST technology. This therefore suggests that the lack of generalization observed on the original test set is mainly driven by its genomic heterogeneity with respect to the training set, and not to the fact that the AST methods used to define the reference MICs differed.

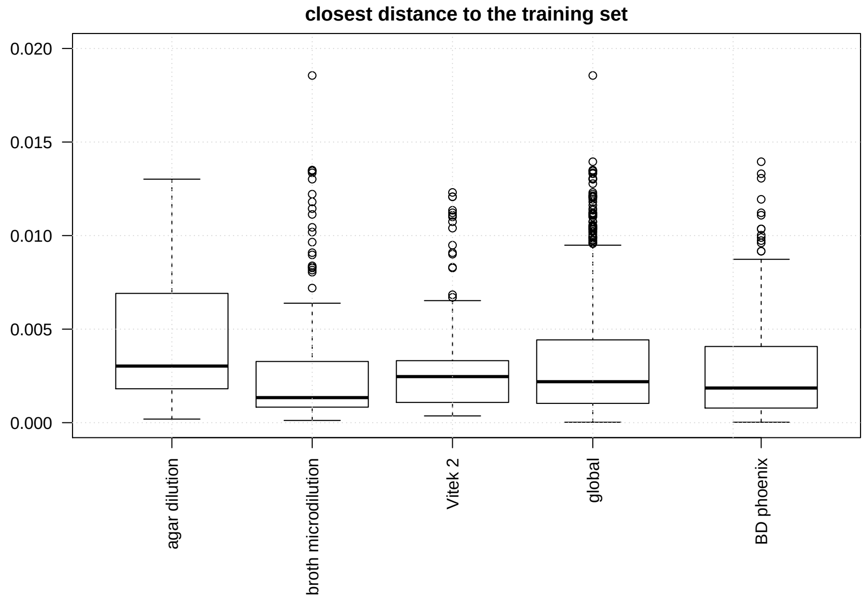

Figure S15: Distribution of closest distance to the training distance.

	BD Phoenix			agar dilution				broth microdilution				VITEK 2			
drug	S	NS	AUC	S	NS	AUC	p-value	S	NS	AUC	p-value	S	NS	AUC	p-value
amikacin	177	23	87.8	0	0	–	–	54	116	81	2.3e-01	106	75	84.2	5.3e-01
aztreonam	61	137	89.2	0	0	–	–	5	141	68.9	2.8e-01	5	109	90.6	8.4e-01
cefepime	83	109	83	0	0	–	–	28	144	81.1	7.1e-01	25	91	**63.2**	**3.2e-02**
cefoxitin	117	80	73.8	112	134	**59.1**	**4.9e-03**	8	62	80.1	4.0e-01	18	123	**94.4**	**2.0e-06**
ceftazidime	50	150	91.9	86	158	92.7	7.6e-01	25	171	93.4	7.4e-01	14	128	94.4	5.8e-01
ciprofloxacin	69	131	95.2	71	161	93.2	3.9e-01	30	166	95.5	9.4e-01	36	144	**99.1**	**8.5e-03**
imipenem	162	38	86.2	219	3	47	5.6e-02	24	137	82	5.3e-01	58	119	90.4	4.0e-01
meropenem	164	33	90.3	0	0	–	–	35	169	84.8	2.9e-01	51	128	**79.4**	**3.8e-02**
piper.tazo	87	111	86.8	130	116	**64.9**	**2.5e-07**	11	156	85.1	8.0e-01	5	110	**98.2**	**4.3e-05**
tetracycline	92	108	83.8	85	156	82.1	6.7e-01	29	43	78.3	3.6e-01	41	74	**92.6**	**1.7e-02**

Table S2: Impact of AST method on prediction performance - summary table. This table provides for each drug the number of susceptible (S) and non-susceptible (NS) strains of the test set characterized by each AST technology, and the corresponding AUCs, obtained by lasso-penalized logistic regression models. It also provides the p-values obtained by testing whether the AUCs measured on strains characterized by agar dilution, broth-microdilution and VITEK 2 are significantly worse or better than the AUC measured on strains characterized by BD-Phoenix. Significant difference at the 0.05 level are shown in bold.

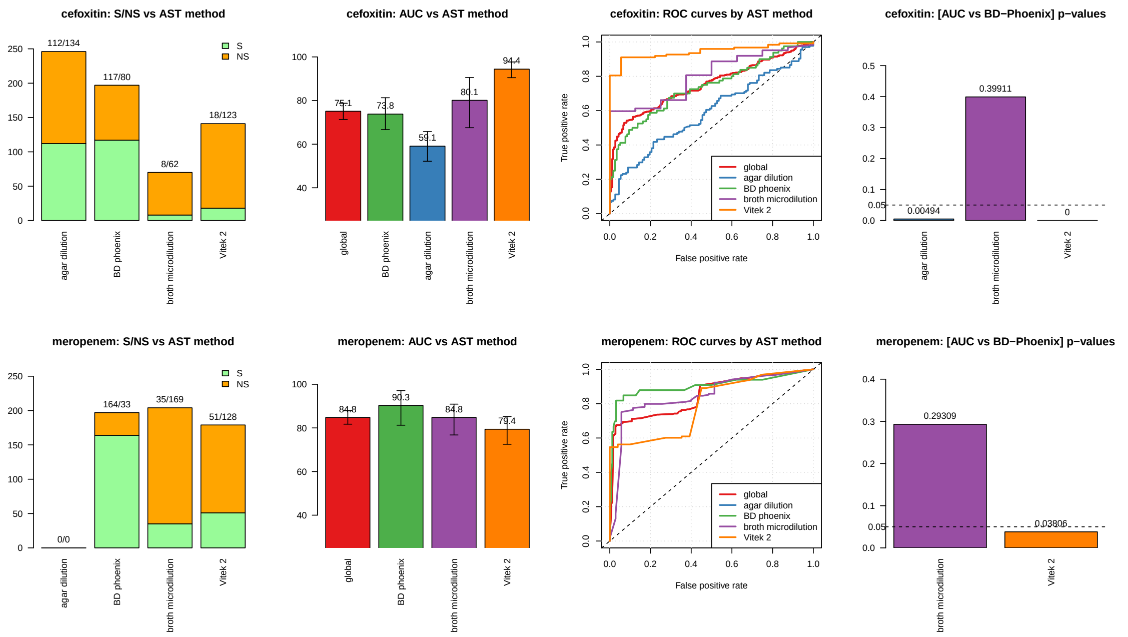

Figure S16: Impact of AST method on prediction performance - detailed results for cefoxitin (top) and meropenem (bottom) . From left to right: (i) number of S/NS strains of the test set characterized by each AST technology ; (ii) AUC obtained by lasso-penalized logistic regression models on each fraction of the test set, and corresponding bootstrap confidence intervals ; (iii) corresponding ROC curves and (iv) p-values obtained by testing whether the AUC obtained from strains characterized by BD-Phoenix is significantly better of worse than that obtained by each alternative AST technology.

S12 Results obtained on other species

Table S3 provides cross-validation results obtained using the process described in the main text and illustrated in Figure S5, on other bacterial species :

- *Staphylococcus aureus*, using data from Gordon et al. [2014] (training panel),
- *Neisseria gonorrhoeae*, using data from Eyre et al. [2017],
- nontyphoidal *Salmonella*, using data from Nguyen et al. [2019],
- and *Mycobacterium tuberculosis*, using data from Davis et al. [2016].

Overall, similar observations were made, namely that the cluster-lasso strategy reaches the same level of performance than the lasso, while providing better interpretability after analyzing the visualizations provided by DBGWAS (data not shown).

species	drug	lasso bACC	lasso AUC	lasso support	lasso unitigs	cluster-lasso bACC	cluster-lasso AUC	cluster-lasso support	cluster-lasso unitigs
S. aureus	ciprofloxacin	96.7	95.7	1	1(1)	96.2	96.8	2	259(251)
	erythromycin	98.7	99.6	14	14(1)	97.9	99.6	16	647(432)
	fusidic acid	91.7	97.4	30	44(9)	92.3	97.6	28	315(131)
	methicillin	99.5	99.5	3	5(3)	99.4	99.7	1	303(303)
	penicillin	98.8	99	1	4(4)	99.4	99.6	1	31(31)
	tetracycline	96.9	96.7	12	15(3)	98.9	99.6	10	47(28)
N. gonorrhoeae	azithromycin	84.2	92.2	97	108(3)	84.4	92.5	67	221(13)
	cefixime	91.7	97.2	45	47(2)	92.1	97.5	40	189(56)
	ciprofloxacin	95.1	96.1	19	24(2)	95.3	94.2	1	8(8)
	penicillin	84.2	91.2	49	58(3)	85	91.3	42	106(7)
	tetracycline	85.7	90.4	36	38(2)	85.2	89.4	32	88(9)
Salmonella	ampicillin	97	98.3	29	31(3)	97	98.3	16	409(139)
	amoxicillin - clavulanic acid	93.9	98.1	73	85(3)	93.7	98	45	670(139)
	ceftriaxone	97.3	96.9	1	3(3)	97.6	97.2	1	139(139)
	chloramphenicol	86.2	92.9	135	162(10)	86	92.5	103	532(65)
	cefoxitin	96.8	96.2	1	3(3)	97.1	96.5	1	139(139)
	gentamicin	96.8	98.2	48	57(7)	96.5	98.2	34	607(363)
	kanamycin	95.7	98.3	25	42(10)	96.1	98.6	23	128(37)
	tetracycline	97.4	98.2	29	44(6)	97.1	98.1	15	755(303)
M. tuberculosis	ethambutol	85.7	91.8	41	49(3)	85.8	91.6	22	114(34)
	ethionamide	78.7	84.5	8	8(1)	79.3	81.6	5	11(4)
	fluoroquinolones	93.4	91.5	1	1(1)	93.4	92.3	5	15(7)
	isoniazid	93.9	94.7	2	2(1)	93.5	94.5	2	8(6)
	kanamycin	91.2	92.4	7	7(1)	91.3	92.1	7	17(5)
	streptomycin	87.3	92.7	49	60(3)	86.5	92.2	26	133(16)
	rifampicin	94.3	96.6	8	8(1)	94.5	97	7	25(11)

Table S3: **Cross-validation results.** This table summarizes the cross-validation results obtained by the lasso and cluster-lasso strategies on other bacterial species, in terms of balanced accuracy (bACC), AUC, support size, overall number of unitigs involved and maximal number of unitigs associated to a single pattern or cluster (between brackets).

References

Thea Brennan-Krohn, Kenneth P. Smith, and James E. Kirby. The poisoned well: Enhancing the predictive value of antimicrobial susceptibility testing in the era of multidrug resistance. *Journal of Clinical Microbiology*, 55(8):2304–2308, 2017. ISSN 0095-1137. doi: 10.1128/JCM.00511-17. URL `https://jcm.asm.org/content/55/8/2304`.

James J. Davis, Sébastien Boisvert, Thomas Brettin, Ronald W. Kenyon, Chunhong Mao, Robert Olson, Ross Overbeek, John Santerre, Maulik Shukla, Alice R. Wattam, Rebecca Will, Fangfang Xia, and Rick Stevens. Antimicrobial resistance prediction in PATRIC and RAST. *Scientific Reports*, 6:27930, 2016.

George M Eliopoulos and Karen Bush. New β-lactamases in gram-negative bacteria: diversity and impact on the selection of antimicrobial therapy. *Clinical Infectious Diseases*, 32(7):1085–1089, 2001.

David W Eyre, Dilrini De Silva, Kevin Cole, Joanna Peters, Michelle J Cole, Yonatan H Grad, Walter Demczuk, Irene Martin, Michael R Mulvey, Derrick W Crook, et al. WGS to predict antibiotic MICs for *Neisseria gonorrhoeae*. *The Journal of Antimicrobial Chemotherapy*, 72 (7):1937–1947, 2017.

N. C. Gordon, J. R. Price, K. Cole, R. Everitt, M. Morgan, Finney Finney, A. M. Kearns, B. Pichon, B. Young, D. J. Wilson, M. J. Llewelyn, J. Paul, T. E. A. Peto, D. W. Crook, A. S. Walker, and T. Golubchika. Prediction of *Staphylococcus aureus* Antimicrobial Resistance by Whole-Genome Sequencing. *Journal of Clinical Microbiology*, 52(4):1182–1191, 2014.

Magali Jaillard, Leandro Lima, Maud Tournoud, Pierre Mahé, Alex van Belkum, Vincent Lacroix, and Laurent Jacob. A fast and agnostic method for bacterial genome-wide association studies: Bridging the gap between k-mers and genetic events. *PLOS Genetics*, 14(11): 1–28, 11 2018. doi: 10.1371/journal.pgen.1007758. URL `https://doi.org/10.1371/journal.pgen.1007758`.

Marcus Nguyen, Thomas Brettin, S Wesley Long, James M Musser, Randall J Olsen, Robert Olson, Maulik Shukla, Rick L Stevens, Fangfang Xia, Hyunseung Yoo, and James J Davis. Developing an in silico minimum inhibitory concentration panel test for *Klebsiella pneumoniae*. *Scientific reports*, 8(1):421, 2018. ISSN 2045-2322. doi: 10.1038/s41598-017-18972-w. URL `http://www.ncbi.nlm.nih.gov/pubmed/29323230http://www.pubmedcentral.nih.gov/articlerender.fcgi?artid=PMC5765115`.

Marcus Nguyen, S. Wesley Long, Patrick F. McDermott, Randall J. Olsen, Robert Olson, Rick L. Stevens, Gregory H. Tyson, Shaohua Zhao, and James J. Davis. Using machine learning to predict antimicrobial mics and associated genomic features for nontyphoidal *salmonella*. *Journal of Clinical Microbiology*, 57(2), 2019. ISSN 0095-1137. doi: 10.1128/JCM.01260-18. URL `https://jcm.asm.org/content/57/2/e01260-18`.

A Novais, C Rodrigues, R Branquinho, P Antunes, F Grosso, L Boaventura, G Ribeiro, and L Peixe. Spread of an ompk36-modified st15 *klebsiella pneumoniae* variant during an outbreak involving multiple carbapenem-resistant *enterobacteriaceae* species and clones. *European journal of clinical microbiology & infectious diseases*, 31(11):3057–3063, 2012.

Brian D. Ondov, Todd J. Treangen, Páll Melsted, Adam B. Mallonee, Nicholas H. Bergman, Sergey Koren, and Adam M. Phillippy. Mash: fast genome and metagenome distance estimation using minhash. *Genome Biology*, 17(1), 2016.

Xavier Robin, Natacha Turck, Alexandre Hainard, Natalia Tiberti, Frédérique Lisacek, Jean-Charles Sanchez, and Markus Müller. proc: an open-source package for r and s+ to analyze and compare roc curves. *BMC Bioinformatics*, 12(1):77, Mar 2011. ISSN 1471-2105. doi: 10.1186/1471-2105-12-77. URL `https://doi.org/10.1186/1471-2105-12-77`.

Yi Yang and Hui Zou. A fast unified algorithm for solving group-lasso penalize learning problems. *Statistics and Computing*, 25(6):1129–1141, November 2015. ISSN 0960-3174. doi: 10.1007/s11222-014-9498-5. URL `http://dx.doi.org/10.1007/s11222-014-9498-5`.

Hui Zou and Trevor Hastie. Regularization and variable selection via the elastic net. *Journal of the royal statistical society: series B (statistical methodology)*, 67(2):301–320, 2005.
