# Supplementary material for: Interpreting k-mer–based signatures for antibiotic resistance prediction
Source: Gigascience. 2020 Oct 17;9(10):giaa110. doi: 10.1093/gigascience/giaa110 (PMC7568433; doi:10.1093/gigascience/giaa110)
Supplement: giaa110_Response_to_Reviewer_Comments_Original_Submission [file giaa110_response_to_reviewer_comments_original_submission.pdf]

Author's Response To Reviewer Comments

Close

We thank the reviewers for their thoughtful and positive comments, and for their suggestions regarding our manuscript entitled “Interpreting k-mer-based signatures for antibiotic resistance prediction”.
You will find attached a file called "answers-to-reviewers.pdf" providing a point-by-point response to these comments, which we believe greatly improve the quality of the manuscript.

The most important modifications made to the manuscript are the following ones :

1. The accompanying R package is now hosted on a dedicated GitLab repository, and we have included additional code to illustrate how to interface this package with DBGWAS, as suggested by Reviewer 1.
2. We have removed the comparison with the models of Nguyen et al. (2018). Further discussing this issue with Reviewer 2 led us to realize that the comparison we made was not as fair as we intended, hence we followed the suggestion of the reviewer to discard it.
3. We have added a dedicated study in the supplementary materials (Supplementary Section S11) to assess the impact of the phenotypic AST method on the performance of the model, a point that was raised by both reviewers.

We also attached as supplementary material a second version of the modified manuscript for the reviewers in which the modifications applied to the original manuscript are highlighted in red.
We hope that based on these changes our manuscript will now be acceptable for publication in GigaScience.

Magali Jaillard, on behalf of co-authors

Close
