# Supplementary material for: Interpreting k-mer–based signatures for antibiotic resistance prediction
Source: Gigascience. 2020 Oct 17;9(10):giaa110. doi: 10.1093/gigascience/giaa110 (PMC7568433; doi:10.1093/gigascience/giaa110)
Supplement: giaa110_Response_to_Reviewer_Comments_Revision_1 [file giaa110_response_to_reviewer_comments_revision_1.pdf]

Author's Response To Reviewer Comments

Close

Dear Editor and Reviewers,

we attached as Supplementary Material a letter addressing all your remarks.

Best regards,
Magali Jaillard, on behalf of co-authors

Close
