# Supplementary material for: Interpreting k-mer–based signatures for antibiotic resistance prediction
Source: Gigascience. 2020 Oct 17;9(10):giaa110. doi: 10.1093/gigascience/giaa110 (PMC7568433; doi:10.1093/gigascience/giaa110)
Supplement: giaa110_Reviewer_1_Report_Original_Submission — Nicole Wheeler -- 4/14/2020 Reviewed [file giaa110_reviewer_1_report_original_submission.pdf]

Reviewer Report

Title: Interpreting k-mer-based signatures for antibiotic resistance prediction

Version: Original Submission **Date:** 4/14/2020

Reviewer name: Nicole Wheeler

Reviewer Comments to Author:

Jaillard and colleagues present an extension to their previous DBGWAS approach to producing efficient representations of pangenome variation, by collapsing k-mers where possible into longer units (unitigs). Here they then cluster these units into groups of unitigs with similar distributions across the population, to produce a richer sequence space to interpret associations between genotype and phenotype.
Firstly, I would like to thank the authors for their clearly reasoned and explained paper, it was a pleasure to read. I am also grateful for the clear figures illustrating the workflow. I only have minor comments/suggestions.
Minor comments:
Please supply a link to the R package in the paper's abstract.
It's not very clear what format the input data need to be in to run the pipeline. Please supply example code for producing input for this algorithm from DBGWAS output, and for visualising the graphs for top predictors using output from the R package.
"We gathered the assembled genomes, provided as contigs, of 1665 strains to develop MIC prediction models for K. pneumoniae"
As the authors discover later in the paper, this is not a good dataset to work from. A critical observation left out of this paper is why - most of the isolates are from only 2 STs. This is a major reason why a lot of known resistance determinants are not represented in the collection.
In the vignette provided with the R package, I see that samples were evenly split by collection site (or by ST in the analysis presented in the manuscript) into training and testing, which removes an opportunity to test the generalisability of the model to new sequence types or locations, and makes it more likely that cv errors will be lower. Block cross-validation where each category in the pop.structure column is entirely placed into training or testing would be a better approach. Of course, with the training dataset selected for this study, this would create very unevenly sized training and testing folds, but this in itself is useful when considering the robustness of the model. Also, why not split the data in the vignette by sequence type, to make the analysis more comparable with the one presented in the paper?
The authors make the argument: "it is hazardous to predict susceptibility when no marker is detected, since the resistance marker may be novel and databases incomplete" in their problem statements before introducing their approach. I don't see how ML, or the approach detailed in this manuscript, deals with this problem.
Pg 6: blaKPC is known to jump frequently between plasmids (1). Is the diversity of the training data sufficient to recognise the relevant mobile unit carrying KPC, and not a specific plasmid? In Figures 4 and 5 it looks like a plasmid may have been assembled and used in its entirety for prediction, potentially indicating a weakness of the cluster-lasso approach relative to the lasso when working from a dataset

that isn't very diverse.
Figure 8B is misleading - I would prefer the minimum y axis to be 0.
I would point out that some of your MIC prediction inaccuracies could be due to the different phenotyping methods used across the sample collection.
Supp Table S4 - please choose either periods or commas to represent decimals.
Supp S5 - CTX-M is not only more common, but also confers a higher MIC than TEM.
"We empirically observed that the weights variability within a cluster was very small, as shown in Supplementary Figure S13, which therefore indicated that keeping the features separated or averaging them is..."
I suspect this may be partly due to the lack of diversity in the training data. Did you check this for the re-partitioned data too?
References
1) David, S., Cohen, V., Reuter, S., Sheppard, A. E., Giani, T., Parkhill, J., the European Survey of Carbapenemase-Producing Enterobacteriaceae (EuSCAPE) Working Group, the ESCMID Study Group for Epidemiological Markers (ESGEM), Rossolini, G. M., Feil, E. J., Grundmann, H., & Aanensen, D. M. (2019). Genomic analysis of carbapenemase-encoding plasmids from Klebsiella pneumoniae across Europe highlights three major patterns of dissemination. In bioRxiv (p. 2019.12.19.873935). https://doi.org/10.1101/2019.12.19.873935

Level of Interest

Please indicate how interesting you found the manuscript: Choose an item.

Quality of Written English

Please indicate the quality of language in the manuscript: Choose an item.

Declaration of Competing Interests

Please complete a declaration of competing interests, considering the following questions:

- Have you in the past five years received reimbursements, fees, funding, or salary from an organisation that may in any way gain or lose financially from the publication of this manuscript, either now or in the future?
- Do you hold any stocks or shares in an organisation that may in any way gain or lose financially from the publication of this manuscript, either now or in the future?
- Do you hold or are you currently applying for any patents relating to the content of the manuscript?
- Have you received reimbursements, fees, funding, or salary from an organization that holds or has applied for patents relating to the content of the manuscript?
- Do you have any other financial competing interests?

- Do you have any non-financial competing interests in relation to this paper?

If you can answer no to all of the above, write 'I declare that I have no competing interests' below. If your reply is yes to any, please give details below.

None to disclose

I agree to the open peer review policy of the journal. I understand that my name will be included on my report to the authors and, if the manuscript is accepted for publication, my named report including any attachments I upload will be posted on the website along with the authors' responses. I agree for my report to be made available under an Open Access Creative Commons CC-BY license (http://creativecommons.org/licenses/by/4.0/). I understand that any comments which I do not wish to be included in my named report can be included as confidential comments to the editors, which will not be published.

Choose an item.

To further support our reviewers, we have joined with Publons, where you can gain additional credit to further highlight your hard work (see: https://publons.com/journal/530/gigascience). On publication of this paper, your review will be automatically added to Publons, you can then choose whether or not to claim your Publons credit. I understand this statement.

Yes Choose an item.
