# Supplementary material for: Interpreting k-mer–based signatures for antibiotic resistance prediction
Source: Gigascience. 2020 Oct 17;9(10):giaa110. doi: 10.1093/gigascience/giaa110 (PMC7568433; doi:10.1093/gigascience/giaa110)
Supplement: giaa110_Reviewer_1_Report_Revision_1 — Nicole Wheeler -- 8/3/2020 Reviewed [file giaa110_reviewer_1_report_revision_1.pdf]

Reviewer Report

Title: Interpreting k-mer-based signatures for antibiotic resistance prediction

Version: Revision 1 **Date:** 8/3/2020

Reviewer name: Nicole Wheeler

Reviewer Comments to Author:

I love the additional content the authors have added to the text - it adds useful info for people wanting to build these models for the first time, and for those thinking about applying pre-built models to their own data.
I would push back on Reviewer 2's assertion that the model comparison originally presented in this manuscript was not helpful for the reader. In the original paper, Reviewer 2's algorithm is presented as an approach that could be used in a clinical laboratory, finishing their discussion by saying "sequence-based MIC prediction appears to be feasible as a diagnostic strategy", and providing an implementation so readers can use the model to make MIC predictions on their own data. Constraining the accurate use of this algorithm to samples collected in the past from a single city is a limitation that should be clearly identified, and at the time of publication, the generalisability of this approach outside the training data was not known. The subsequent realisation of the consequences of the lack of diversity in this collection is important in terms of data collection strategies to train these algorithms (as demonstrated by the authors of this paper choosing to train an algorithm with the same dataset and only realising the consequences during testing on a dataset collected in another setting), and also for people who consider using the Nguyen algorithm for their own research. The findings also highlight the limitations of only using cross-validation to assess accuracy in an ML study (a practice which is still quite common due to the lack of good, independent datasets for training and testing these models). Fortunately, most of these issues have been retained in the text, even though an explicit performance comparison is has been removed, so my only request here is that no further information be removed that would benefit the reader.
It strikes me that, as a single final improvement to the manuscript, "generalisation" in this context needs to be defined. What is the algorithm supposed to be learning, and under what circumstances is it expected to perform? In my thinking, generalisation can be defined by the ability to correctly identify resistance due to a previously seen mechanism in a new genetic background, hence the importance of partitioning STs into different train/test folds/partitions, and the importance of testing previously published algorithms on new datasets. But I think a different definition is being used here, where generalisation means an algorithm having widely sampled the genetic diversity of the pathogen being considered during training, and correctly identifying resistant strains within familiar genetic backgrounds upon deployment. Clarifying the meaning of this word in the text would be a good step toward the responsible development and use of ML algorithms in this area.

Level of Interest

Please indicate how interesting you found the manuscript: Choose an item.

Quality of Written English

Please indicate the quality of language in the manuscript: Choose an item.

Declaration of Competing Interests

Please complete a declaration of competing interests, considering the following questions:

- Have you in the past five years received reimbursements, fees, funding, or salary from an organisation that may in any way gain or lose financially from the publication of this manuscript, either now or in the future?
- Do you hold any stocks or shares in an organisation that may in any way gain or lose financially from the publication of this manuscript, either now or in the future?
- Do you hold or are you currently applying for any patents relating to the content of the manuscript?
- Have you received reimbursements, fees, funding, or salary from an organization that holds or has applied for patents relating to the content of the manuscript?
- Do you have any other financial competing interests?
- Do you have any non-financial competing interests in relation to this paper?

If you can answer no to all of the above, write 'I declare that I have no competing interests' below. If your reply is yes to any, please give details below.

I declare that I have no competing interests

I agree to the open peer review policy of the journal. I understand that my name will be included on my report to the authors and, if the manuscript is accepted for publication, my named report including any attachments I upload will be posted on the website along with the authors' responses. I agree for my report to be made available under an Open Access Creative Commons CC-BY license (http://creativecommons.org/licenses/by/4.0/). I understand that any comments which I do not wish to be included in my named report can be included as confidential comments to the editors, which will not be published.

Choose an item.

To further support our reviewers, we have joined with Publons, where you can gain additional credit to further highlight your hard work (see: https://publons.com/journal/530/gigascience). On publication of this paper, your review will be automatically added to Publons, you can then choose whether or not to claim your Publons credit. I understand this statement.

Yes Choose an item.
