# Supplementary material for: Interpreting k-mer–based signatures for antibiotic resistance prediction
Source: Gigascience. 2020 Oct 17;9(10):giaa110. doi: 10.1093/gigascience/giaa110 (PMC7568433; doi:10.1093/gigascience/giaa110)
Supplement: giaa110_Reviewer_2_Report_Original_Submission — James J. Davis -- 5/2/2020 Reviewed [file giaa110_reviewer_2_report_original_submission.pdf]

Reviewer Report

Title: Interpreting k-mer-based signatures for antibiotic resistance prediction

Version: Original Submission **Date:** 5/2/2020

Reviewer name: James J. Davis

Reviewer Comments to Author:

The prediction of AMR phenotypes from genome sequence data is an active machine learning research problem. Several papers have shown that using k-mers as input features can provide fairly accurate models for predicting phenotypes without the need for characterized AMR genes. However, one caveat of this approach is that most prior studies have used small nucleotide k-mers ranging in size from 10-31 nucleotides. This is a cumbersome approach, requiring large memory machines, and in certain cases, these models can be difficult to interpret. The manuscript by BioMerieux group attempts to address these problems.

First for good things. I think the paper is well written and referenced and that the approach is incisive and would be a welcomed contribution to the field, and so I do not wish to be disparaging to the underlying work on the problem. However, I do not think the manuscript is publishable in its current form.

A major issue with the work is that the comparisons that are made to Nguyen et al. are not scientifically sound. After going through the paper and considering the comparisons that were made to Nguyen et al., I don't think that any of these comparisons were made using matched training and testing data. Because of this, I don't think that any of the comparisons are helpful to the authors or the reader.

1. Before going into what corrective measures could be taken, I would like to say that in my opinion the paper is probably publishable without any comparisons to Nguyen study. The manuscript in hand is well written, unique, solves a major problem, and provides accuracies that are competitive with the best models published to date.
2. If the authors wish to salvage the comparison to Nguyen here are some recommendations: They could train a S vs. NS classifier using the k-mer based Nguyen approach on the same set of genomes and then compare the results to the results from their model. If the authors can't do this because of the high computational overhead, the next best thing might be to build one of their models on the Nguyen subset of genomes and then do the comparison. If the primary goal of the paper is to argue for generalizability, then the authors should consider training their models on the Nguyen subset of Klebsiella strains and then compute an error rate on the held out European set of genomes to see if their method actually performs better. Similar matched comparisons are also necessary in Table S3 and Figure S5. Also, I would recommend adding the details of how models were compared to the methods section, that isn't currently there.
3. Nguyen et al. were reporting error rates within +/- 1 two-fold dilution step. I think an argument could be made in favor of, or against this, but it might be worth thinking about.
4. Also, there is a great deal of variability between laboratory testing methods. Our group has built models like this in various ways and we have found that training a model on data generated with one

method (say with BDphoenix) will produce bad results on held out data that is generated based on Vitek2 or E-test, even if the genomes are sourced from the same location and have similar underlying distributions of AMR genes. Again, circling back to the need for having matched training and testing data for making comparisons. If the authors could demonstrate better generalizability across AST methods, this would probably be an achievement.
The authors should explicitly state why Logistic Regression was the method of choice. What happens if another ML method is plugged in at this step? Was any evaluation of other methods performed?
I did not come away with a firm understanding of the size of a matrix built from "unitigs" vs. one built from kmers vs one built from alignments like they did in the Palsson papers.
Why did the authors choose 31mers? Is there data supporting this decision, does it lead to better "unitigs"? Is there a size that is too small? Does k-mer size impact compute times?
In the current data set, to my knowledge, there is only one SNP-based AMR mechanism, which is cipro. The rest is horizontal gene transfer mechanisms. How does the method cope with SNP-based methods? How well does it cope with more diverse data sets or different species? For instance, the Nguyen Klebsiella strains are almost entirely ESBL producers that mainly come from 2 MLST types, and were sourced from one city. This probably resulted in optimistic accuracies for their study, and could also be skewing the current study, since those genomes represent nearly 70% of the data set.
Finally, over the years, many students have been drifting from R to python. Is a python implementation feasible, or do the dependencies prohibit this?

Level of Interest

Please indicate how interesting you found the manuscript: Choose an item.

Quality of Written English

Please indicate the quality of language in the manuscript: Choose an item.

Declaration of Competing Interests

Please complete a declaration of competing interests, considering the following questions:

- Have you in the past five years received reimbursements, fees, funding, or salary from an organisation that may in any way gain or lose financially from the publication of this manuscript, either now or in the future?
- Do you hold any stocks or shares in an organisation that may in any way gain or lose financially from the publication of this manuscript, either now or in the future?
- Do you hold or are you currently applying for any patents relating to the content of the manuscript?
- Have you received reimbursements, fees, funding, or salary from an organization that holds or has applied for patents relating to the content of the manuscript?

- Do you have any other financial competing interests?
- Do you have any non-financial competing interests in relation to this paper?

If you can answer no to all of the above, write 'I declare that I have no competing interests' below. If your reply is yes to any, please give details below.

none

I agree to the open peer review policy of the journal. I understand that my name will be included on my report to the authors and, if the manuscript is accepted for publication, my named report including any attachments I upload will be posted on the website along with the authors' responses. I agree for my report to be made available under an Open Access Creative Commons CC-BY license (http://creativecommons.org/licenses/by/4.0/). I understand that any comments which I do not wish to be included in my named report can be included as confidential comments to the editors, which will not be published.

Choose an item.

To further support our reviewers, we have joined with Publons, where you can gain additional credit to further highlight your hard work (see: https://publons.com/journal/530/gigascience). On publication of this paper, your review will be automatically added to Publons, you can then choose whether or not to claim your Publons credit. I understand this statement.

Yes Choose an item.
